# Range-Wide Genetic Analysis of Little Brown Bat (*Myotis lucifugus*) Populations: Estimating the Risk of Spread of White-Nose Syndrome

**DOI:** 10.1371/journal.pone.0128713

**Published:** 2015-07-08

**Authors:** Maarten J. Vonhof, Amy L. Russell, Cassandra M. Miller-Butterworth

**Affiliations:** 1 Department of Biological Sciences, Western Michigan University, Kalamazoo, Michigan, United States of America; 2 Environmental and Sustainability Studies Program, Western Michigan University, Kalamazoo, Michigan, United States of America; 3 Department of Biology, Grand Valley State University, Allendale, Michigan, United States of America; 4 Penn State Beaver, Monaca, Pennsylvania, United States of America; Aristotle University of Thessaloniki, GREECE

## Abstract

The little brown bat (*Myotis lucifugus*) is one of the most widespread bat species in North America and is experiencing severe population declines because of an emerging fungal disease, white-nose syndrome (WNS). To manage and conserve this species effectively it is important to understand patterns of gene flow and population connectivity to identify possible barriers to disease transmission. However, little is known about the population genetic structure of little brown bats, and to date, no studies have investigated population structure across their entire range. We examined mitochondrial DNA and nuclear microsatellites in 637 little brown bats (including all currently recognized subspecific lineages) from 29 locations across North America, to assess levels of genetic variation and population differentiation across the range of the species, including areas affected by WNS and those currently unaffected. We identified considerable spatial variation in patterns of female dispersal and significant genetic variation between populations in eastern versus western portions of the range. Overall levels of nuclear genetic differentiation were low, and there is no evidence for any major barriers to gene flow across their range. However, patterns of mtDNA differentiation are highly variable, with high Φ_ST_ values between most sample pairs (including between all western samples, between western and eastern samples, and between some eastern samples), while low mitochondrial differentiation was observed within two groups of samples found in central and eastern regions of North America. Furthermore, the Alaskan population was highly differentiated from all others, and western populations were characterized by isolation by distance while eastern populations were not. These data raise the possibility that the current patterns of spread of WNS observed in eastern North America may not apply to the entire range and that there may be broad-scale spatial variation in the risk of WNS transmission and occurrence if the disease continues to spread west.

## Introduction

Understanding how host movement patterns influence the transmission of pathogens is critical to the development of effective prevention and control strategies, and to the conservation and management of host populations during and after disease outbreaks. However, for many host species, data on individual movements and contact rates are difficult or impossible to collect because of cryptic behavior, the geographic scale of movements, or methodological considerations that limit our ability to follow individuals through time and space. Evidence from empirical studies employing population and landscape genetic approaches has demonstrated that landscape features, such as mountains and rivers that limit host gene flow, often represent barriers to disease transmission [1–6], although alternative mechanisms of pathogen dispersal, including humans and other highly mobile intermediate hosts, may override the influence of primary host population genetic structure [1]. Nevertheless, where they exist, such barriers to host gene flow can have a dramatic impact on initial disease establishment, the rate and direction of disease spread, spatial patterns of host resistance, and dynamics and genetic structure of pathogen populations [1–6]. Assuming that rates of contact among individuals leading to gene flow are indicative of contacts that could result in disease transmission, genetic methods provide a useful alternative to traditional demographic approaches as a means of examining host movements and their impact on disease transmission [1].

White-nose syndrome (WNS) is an emerging fungal disease causing high levels of mortality in hibernating North American bats [7–9]. The causative agent, *Pseudogymnoascus destructans* (hereafter *Pd*), is a cold-loving fungus that affects bats during hibernation and subsequent arousal. *Pd* causes characteristic cup-like erosions of the epidermis of the wings and muzzle, and may invade sebaceous glands and hair follicles [10]. Mortality of bats likely occurs through the loss of physiological homeostasis [11], possibly associated with dehydration and electrolyte depletion [12,13], leading to more frequent arousal behavior and premature loss of fat reserves [14,15]. Since it was first discovered in New York State during the winter of 2006–07, WNS has since spread to 27 additional states and five Canadian provinces, and is known to affect at least seven species of hibernating bats [16]. Mortality rates vary considerably among species but can be very high (>90% for little brown bats, *Myotis lucifugus*, and northern long-eared bats, *M*. *septentrionalis* [9]), and cumulative mortality of all affected bat species has been estimated at 5.7 to 6.7 million individuals as of January 2012 [17]. The rapid emergence, and the geographic and taxonomic spread of the disease have raised serious concerns about the long-term survival of hibernating bat species in eastern North America, and have highlighted our lack of knowledge of the factors that may influence WNS transmission and spread to currently unaffected regions.

Little brown bats were among the first species to be diagnosed with WNS [7], and population models indicate that if mortality rates stay constant, this species could be extirpated from the northeastern United States within 16 years [18]. Hibernating populations of all sizes have been affected by WNS, but the probability of infection increases with increasing colony size [19,20], although mortality within populations is density-independent and characterized by frequency-dependent transmission [21]. Thus, there is a high probability that little brown bat populations in currently affected regions will be highly reduced and possibly be extirpated in coming decades. Additionally, WNS may pose a threat to the entire species if the disease continues to spread across the species’ range. Further, because little brown bats are one of two affected bat species whose geographic ranges span temperate North America, they may drive transmission of *Pd* to a novel suite of western North American hibernating bat species that might otherwise remain geographically isolated from the disease. Therefore, it is crucial that we understand the probability of *Pd* transmission across the range of little brown bats, and whether there are barriers to gene flow that could restrict the geographic spread of WNS.

**Fig 1:**
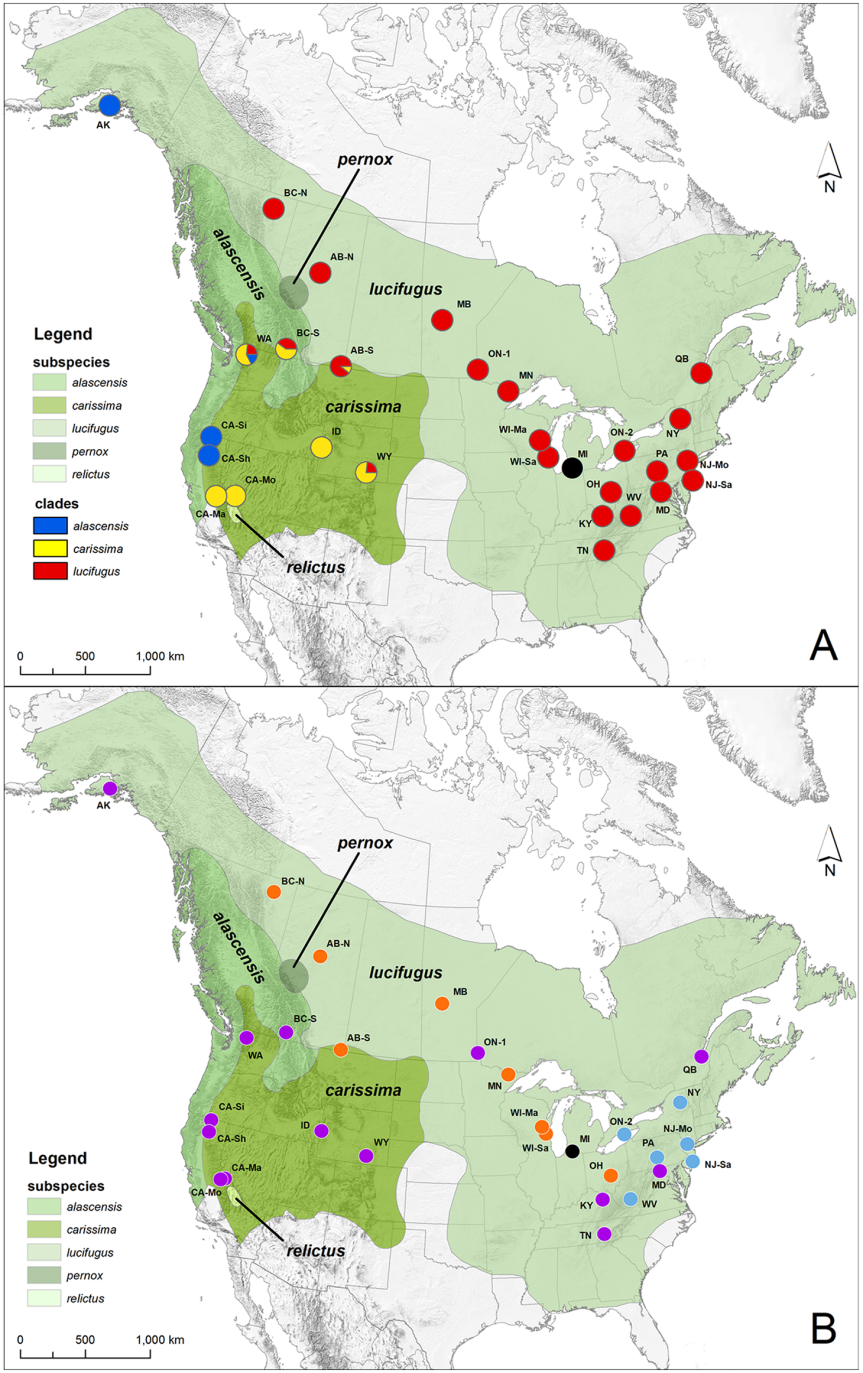
Map of Canada and the United States showing the distribution of described *Myotis lucifugus* subspecies (modified from [17]) and sampling locations. a) shows sampling locations with pie charts indicating frequencies of mtDNA subspecific clades (subspecific designations are indicated in the legend and colors follow those used in Fig 2) in each population, while b) shows groupings of populations (orange and blue dots) within which pairwise Φ_ST_ values based on mtDNA haplotype frequencies were low versus populations that were significantly differentiated from all other populations (purple dots; high pairwise Φ_ST_ values with all other sampled populations). One sampled population in Michigan (shown with a black dot in a) was not included in mtDNA analyses. Population abbreviations are detailed in Table 1, and colors in pie charts in a) correspond to clades shown in Fig 2. Data sources for the map include: nationalatlas.gov, iucnredlist.org, and ESRI Data & Maps 2006 through ArcGIS (S1 File).

Here we apply genetic approaches to understand levels of gene flow and population connectivity in the little brown bat. This small (6–10 g) insectivorous bat species is among the most widespread (Fig 1) and well-studied in North America [22,23]. During the summer, reproductive females form maternity colonies in buildings, trees, or crevices where parturition and post-natal care take place, while males and non-reproductive females typically roost solitarily [22]. In winter, both sexes congregate in hibernacula, and mating takes place during the pre-hibernation swarming period, or during hibernation itself [24,25]. The size of hibernating populations may vary considerably, on the order of 10’s to 100,000’s, but most of the larger known hibernacula occur in karst regions of eastern North America, and very little is known about the distribution or size of hibernacula in western North America. Because individuals from many breeding groups come together at swarming or hibernation sites with males that may or may not have originated from the same breeding group [26,27], these sites have been suggested to represent ‘hot spots’ of gene flow for temperate bats [28–30]. Thus, patterns of gene flow will represent the interplay of movements of individuals between summer and/or winter populations, and levels and spatial patterns of connectivity among summer and winter populations that determine the composition of mating aggregations.

There are currently five recognized subspecies of little brown bats (*M*. *l*. *alascensis*, *M*. *l*. *carissima*, *M*. *l*. *lucifugus*, *M*. *l*. *pernox*, and *M*. *l*. *relictus* [22,31]; see Fig 1) based on morphology, but the extent to which these subspecies diverge genetically is unclear. Coalescent analyses of nuclear DNA (nucDNA) and mitochondrial DNA (mtDNA) suggest that some subspecies may represent independent evolutionary lineages, but that *M*. *lucifugus* may be paraphyletic with respect to the western long-eared bat, *M*. *evotis* [32]. In addition, two distinct mtDNA lineages (corresponding to *M*. *l*. *carissima* and *M*. *l*. *lucifugus*) co-occur in southern Alberta and north-central Montana, but these two groups of bats are not differentiated based on nuclear microsatellite DNA or morphology, suggesting that the subspecies in question may interbreed and exchange genes [33]. A single mitochondrial lineage corresponding to *M*. *l*. *lucifugus* was observed in the Minnesota populations, and there was a strong signal of population expansion dating to 18 kya [34]. Environmental niche modeling based on conditions during the Last Glacial Maximum (LGM) indicated the presence of a single large refugium extending across the southeastern and south-central United States, and more fragmented refugia in the southern portion of the mountainous western United States [34], suggesting a possible mechanism for lineage differentiation within this species where separation into disjunct glacial refugia was followed by subsequent post-glacial range expansion and secondary contact.

Few studies have examined genetic variation in little brown bats, and there has been no comprehensive range-wide population genetic analysis of this species. Fine-scale genetic studies in Minnesota described high levels of mtDNA structure and weak but significant nucDNA structure among maternity colonies [35], a pattern consistent with many other temperate bat species characterized by female philopatry to summer breeding habitat and extensive gene flow via mating at swarming sites during the autumn [36–41]. In southestern Canada, high levels of genetic connectivity were identified among swarming sites, however minimal structuring at both mtDNA and nucDNA markers suggested dispersal may occur in both sexes, although it may be male-biased [42]. In Pennsylvania, no significant nuclear structure was identified among hibernating populations, but these populations were structured matrilineally. This mtDNA structure was correlated with local topography, which may have delayed the spread of WNS to western parts of the state [6].

The rapid spread of WNS through eastern North American populations of little brown bats (and other affected species) suggests that few barriers to transmission exist within the current range of the disease. Here we utilize mtDNA sequence and nucDNA microsatellite variation from a large sample of little brown bats collected across the range of the species to address the following objectives: 1) assess levels of genetic variation in little brown bat populations, including areas affected by WNS and those currently unaffected; 2) quantify genetic differentiation among populations sampled across the range of the species, including populations in eastern North America within the current range of WNS, as well as additional populations situated both east and west of the transition between the Great Plains and Rocky Mountains; and 3) assess the current geographic distribution of and levels of genetic differentiation among currently-recognized subspecific lineages.

There are few physiographic barriers that would limit movement of highly vagile organisms east of the Rocky Mountains. Phylogeographic studies of widespread bats and birds in North America typically report little differentiation among populations within eastern and central portions of North America, significant differentiation among eastern and western populations, and higher levels of differentiation among populations within the mountainous west [41,43–46]. We predict that the Rocky Mountains will represent a barrier to gene flow, and that we will therefore observe genetic differentiation between sample sites east versus west of the Great Plains-Rocky Mountains transition. Further, because of higher topographic variability, we predict higher levels of genetic differentiation among sample sites in the mountainous west than in eastern North America. If subspecific lineages represent reproductively-isolated units that arose during the LGM, then we predict that patterns of differentiation at both nucDNA and mtDNA markers will match the described geographic distribution of subspecies. Our study provides valuable data on population connectivity and hence opportunities for WNS transmission across the range of little brown bats that may be used to inform the management and conservation of affected species.

## Methods

### Sample collection

Tissue samples were obtained during the summer (between May and August) from 637 individuals at 29 locations across the range of little brown bats (Table 1, S1 Table, and Fig 1). Two 3 mm biopsy punches, one from each wing, were taken from each bat and stored in 5 M NaCl with 20% DMSO [47]. The bats were released after sampling. The majority of population samples were collected at maternity colonies (N = 16) or single or several closely-spaced (<10 km) netting sites (N = 12). However, the Idaho sample constituted bats collected in 8 different counties in the southeastern portion of the state. When samples came from more than one capture location, centroids were calculated and used as approximate sample locations.

**Table 1:** Sampled little brown bat populations and diversity statistics for mitochondrial COI sequences (*N*_SEQ_, number of individuals sequenced; *N*_HAP_, number of haplotypes; *N*_HAP-UN_, number of haplotypes unique to a site; *h*, haplotype diversity; *π*, nucleotide diversity), and microsatellite genotypes (*N*_GEN_, number of individuals genotyped; *H*_O_, observed heterozygosity; *H*_E_, expected heterozygosity; *N*_A_, mean number of alleles per locus; *AR*, allelic richness; *AR-P*, private allelic richness; *F*_IS_, inbreeding coefficient).

Province or State	County	Abbrev.	WNS-Status	*N*_SEQ_	*N*_HAP_	*N*_HAP-UN_	*h*	*π*	*N*_GEN_	*H*_O_	*H*_E_	*N*_A_	*AR*	*AR-P*	*F*_IS_
Alaska	Kenai Peninsula	AK	Neg	18	5	5	0.65	0.0017	18	0.864	0.814	8.0	7.67	0.01	-0.061
California	Mariposa	CA-Ma	Neg	10	2	1	0.20	0.0016	0						
California	Mono	CA-Mo	Neg	15	2	1	0.48	0.0008	0						
California	Siskiyou	CA-Si	Neg	20	7	6	0.84	0.0026	20	0.922	0.920	13.2	12.20	0.12	-0.003
California	Shasta	CA-Sh	Neg	11	2	1	0.18	0.0014	0						
Washington	Skagit	WA	Neg	17	8	6	0.64	0.0175	17	0.869	0.915	12.2	11.80	0.17	0.050
British Columbia-South		BC-S	Neg	30	7	6	0.61	0.0157	30	0.889	0.903	14.6	11.80	0.12	0.015
Idaho	Multiple	ID	Neg	26	6	4	0.52	0.0038	23	0.860	0.904	13.6	11.89	0.26	0.049
Wyoming	Carbon	WY	Neg	30	9	7	0.79	0.0148	30	0.870	0.904	15.0	12.09	0.02	0.037
Alberta-South		AB-S	Neg	28	9	6	0.66	0.0103	29	0.920	0.918	16.1	12.92	0.12	-0.002
Alberta-North		AB-N	Neg	28	9	5	0.79	0.0045	29	0.900	0.916	15.9	12.76	0.26	0.017
British Columbia-North		BC-N	Neg	15	8	5	0.70	0.0040	15	0.919	0.915	13.3	13.33	0.14	-0.003
Manitoba		MB	Neg	11	4	1	0.75	0.0044	0						
Ontario		ON-1	Neg	15	7	7	0.89	0.0066	0						
Minnesota	St Louis	MN	Neg	20	7	3	0.73	0.0046	20	0.917	0.894	13.8	12.29	0.29	-0.025
Wisconsin	Marquette	WI-Ma	Neg	20	9	4	0.87	0.0057	20	0.889	0.895	13.6	12.04	0.39	0.007
Wisconsin	Sauk	WI-Sa	Neg	20	7	2	0.80	0.0045	16	0.868	0.905	12.0	11.70	0.43	0.041
Michigan	Cass	MI	Neg	0					20	0.828	0.881	13.0	11.64	0.65	0.061
Kentucky	Rowan	KY	Pos	29	13	11	0.84	0.0043	33	0.886	0.900	15.7	12.19	0.33	0.016
Ohio	Fairfield	OH	Pos	31	11	5	0.88	0.0059	30	0.863	0.881	14.3	11.27	0.26	0.021
Tennessee	Blount	TN	Pos	32	4	1	0.46	0.0008	30	0.852	0.905	14.8	11.91	0.23	0.059
West Virginia	Raleigh	WV	Pos	20	7	3	0.74	0.0033	0						
Ontario		ON-2	Pos	30	12	4	0.80	0.0031	0						
Pennsyl-vania	Blair	PA	Pos	21	12	4	0.88	0.0035	21	0.828	0.894	13.6	11.90	0.16	0.018
Maryland	Washington	MD	Pos	34	12	10	0.91	0.0041	30	0.886	0.896	16.0	12.15	0.22	0.004
New York	Jefferson	NY	Pos	20	13	8	0.93	0.0036	18	0.863	0.900	13.0	12.08	0.18	0.026
New Jersey	Morris	NJ-Mo	Pos	30	12	4	0.90	0.0041	31	0.852	0.894	15.0	11.93	0.29	0.045
New Jersey	Salem	NJ-Sa	Pos	20	7	2	0.78	0.0024	0						
Quebec		QB	Pos	16	7	4	0.78	0.0019	30	0.848	0.890	14.3	11.43	0.1	0.047
Overall				617	7.8	4.5	0.72	0.0051	510	0.876	0.897	13.9	11.90	0.23	0.020

Populations are ordered west to east. WNS-Status denotes sampling localities within states or provinces that were positive or negative for WNS as of 2012.

### Ethics statement

One author (MJV) collected samples for this study from one population in Michigan. The samples were collected under permission granted by the State of Michigan Department of Natural Resources (Permit SC 1257), and the methods were approved by the Western Michigan University Institutional Animal Care and Use Committee Protocol Number 08-05-03. All other samples were collected by university and government researchers performing other research who were required to have appropriate permits and other necessary permission to undertake their work.

### Mitochondrial DNA sequencing and analysis

Total genomic DNA was isolated using DNeasy Tissue Kits (Qiagen, Valencia CA). We amplified and sequenced a 636 bp fragment of the mitochondrial cytochrome c oxidase subunit I (COI) gene using primers HCO2198 and LCO1490 [48] or primers VF1 and VR1 [49]. Bats from all sample sites except Michigan were sequenced, for a total of 617 individuals. PCRs were conducted in 25 μl volumes containing 0.4 μM of each primer and 20–50 ng of DNA template, using Illustra PuReTaq Ready-To-Go PCR beads (GE Healthcare Life Sciences, Pittsburgh PA). When reconstituted to 25 μl with water, these beads contained 2.5 units PuReTaq DNA polymerase, 200 μM each dNTP in 10 mM Tris-HCl (pH 9), 50 mM KCl, 1.5 mM MgCl_2_, and an unspecified concentration of bovine serum albumin (BSA). No other additives were added to the solution. Cycling conditions consisted of one cycle of 5 min at 94°C, 30 cycles of 30 sec at 94°C, 45 sec at 68°C and 1 min at 72°C, and a final cycle of 2 min at 72°C. PCR products were purified by digestion with exonuclease I and shrimp alkaline phosphatase (EXOSAP), and were sequenced in both directions, using the amplification primers, at the University of Arizona Genetics Core Facility. Sequences were edited using CodonCode Aligner 3.0 (Gene Codes Corp.) and aligned using the default settings in MAFFT [50].

### Microsatellite genotyping and analysis

We genotyped individuals at eleven highly variable microsatellite loci using primers previously developed for other vespertilionid bats (IBat CA5, CA11, CA43, CA47, and M23 [51]; MS3D02 and MS3F05 [52]; E24 and G9 [53]; Cora_F11_C04 [54]; Coto_G02F_H10R [55]). We did not genotype population samples with ≤15 individuals (CA-Ma, CA-Mo, CA-Sh, MB-1, MB-2; Table 1), with the exception of BC-N that had been genotyped for another study. Based on preliminary results of low levels of differentiation in eastern North America and to reduce costs, we did not genotype individuals from three additional sites east of the Rocky Mountains (ON-2, WV, and NJ-Sa). The total number of individuals genotyped was 510. Amplifications were carried out in four multiplex reactions and two single-locus amplifications (see S2 Table), and were subsequently pooled into three different loads for fragment analysis on an ABI 3130 sequencer. The basic cycling conditions consisted of 1 min at 94°C, three cycles of 30 sec at 94°C, 20 sec at T_a_ (54 or 60°C), and 5 sec at 72°C, 33 cycles of 15 sec at 94°C, 20 sec at T_a_, and 10 sec at 72°C, followed by a final extension at 72°C for 30 min. Some amplifications required additional cycles or the removal of the final extension step (S2 Table). Fragments were analyzed and scored using GeneMarker software (SoftGenetics LLC, State College, PA).

### Mitochondrial DNA analysis

To describe overall levels of mtDNA diversity within populations, we calculated haplotype (*h*) and nucleotide (π) diversities in DNASP v.5.10.1 [56], and determined the number of private haplotypes in each site after collapsing sequences from the entire dataset to unique haplotypes using FABOX [57]. We used several population genetic approaches to establish whether current patterns of variation are indicative of the presence of distinct genetic clusters. We calculated pairwise Φ_ST_ values between sites and tested for significance with 10,000 permutations in Arlequin v.3.11 [58] to identify pairs of sites that were genetically distinct. We also performed an analysis of molecular variance (AMOVA [59]) to describe the relative amount of genetic variation within and among sites. Based on initial pairwise Φ_ST_ results, we then performed nested AMOVAs to identify natural groups of sites. Sites were initially grouped together if they had low pairwise Φ_ST_ values, and the analysis was rerun. Any ambiguous sites (sites that had low Φ_ST_ values with sites in more than one group) were sequentially moved between groups and the analysis was rerun. All logical combinations were tested to identify the grouping that minimized among-site/within-group variation and maximized between-group variation.

To test the significance of defined subspecific lineages within *M*. *lucifugus* using our nationwide dataset, we used a maximum likelihood phylogenetic approach implemented in PhyML v.3.0 [60]. We sequenced COI for other North American *Myotis* spp. (*M*. *californicus*, *M*. *ciliolabrum*, *M*. *evotis*, *M*. *keenii*, *M*. *leibii*, *M*. *sodalis*, *M*. *thysanodes*, and *M*. *volans*; cf [61]), and a member of the Neotropical *Myotis* clade (*M*. *austroriparius*), which was used as the outgroup (see S3 Table for list of specimens). We used the best fit model of sequence evolution (HKY+G) as determined using Mega v.5.0 [62], with the gamma distribution of variability of rates among sites calculated empirically from the data, SPR moves to explore tree space, and SH-Like procedure to assess branch supports [60]. The proportion of each sampled population falling within each subspecific clade was then calculated and plotted on a map produced in ARC-GIS v.10.1 to visualize the geographic distribution of the clades.

### Microsatellite DNA analysis

Deviations from Hardy-Weinberg equilibrium (HWE) were estimated for each locus, and loci were confirmed to be in linkage equilibrium using FSTAT v.2.9.3 [63]. To test for differences in levels of genetic diversity among sites and regions, several indices of nuclear genetic diversity were estimated, including number of alleles per locus, allelic richness, and the inbreeding coefficient (*F*_*IS*_) using FSTAT, private allelic richness using HP-RARE v.1.0 [64], and observed and expected heterozygosity using GENODIVE [65]. We then tested for differences among sites (or groups of sites) in allelic richness and *F*_IS_ in FSTAT, and expected heterozygosity in GENODIVE, using 10,000 permutations. Tests were performed among clusters of sites identified using clustering techniques (see below), and among sites falling within states or provinces that were WNS-positive (KY, OH, TN, WV, ON-2, PA, MD, NY, NJ-Mo, NJ-Sa, QB; Table 1) or WNS-negative (all other sites) as of 2012, although it should be noted that tissues were collected prior to the emergence of WNS at these localities in all cases.

We applied three different approaches to determine the most likely number of distinct genetic clusters independent of original sampling locations, as different clustering algorithms can produce different solutions and concordance among multiple techniques is suggestive of the presence of a strong genetic signal [66]. First, we utilized the model-based Bayesian clustering approach in STRUCTURE v.2.3.3 [67,68] with population membership as a prior [69]. To determine the optimal number of clusters (*K*), we ran 10 runs per *K*, for *K* = 1–10, with a 100,000 MCMC iteration burn-in followed by 400,000 iterations using the admixture model with correlated allele frequencies. The most likely number of clusters was determined using the Evanno et al. [70] method implemented in the program STRUCTURE HARVESTER [71]. The Evanno et al. [70] method is not informative for the highest and lowest *K*; therefore, if the highest log likelihood value was observed for *K* = 1 or 10 across all replicates, we accepted that as the *K* with the highest probability. For the best value of *K* we used CLUMPP [72] to harmonize individual assignments to clusters.

Second, we applied the *K* means clustering approach in GENODIVE v.2.0 as outlined by Meirmans [65]. This approach is based on an AMOVA framework and uses a simulated annealing algorithm to minimize the among-populations/within-groups sum of squares through maximization of *F*_CT_, the variance among clusters relative to the total variance. We determined the most likely number of clusters using the Pseudo-*F* summary statistic, which performs better than the alternative Bayesian Information Criterion (BIC) when migration rates are high and mating is random [65].

The third approach was that of Duchesne and Turgeon [73] implemented in the software FLOCK. Samples are randomly partitioned into *K* clusters (≥2), allele frequencies are estimated for each of the *K* clusters, and each genotype is then reallocated to the cluster with the highest likelihood score. Repeated reallocation based on likelihood scores (20 iterations per run) resulted in genetically homogeneous clusters within a run. Fifty runs were carried out for each *K*, and at the end of each run the software calculated the log likelihood difference (LLOD) score for each genotype (the difference between the log likelihood of the most likely cluster for the genotype and that of its second most likely cluster) and the mean LLOD over all genotypes. Strong consistency among runs (resulting in ‘plateaus’ of identical mean LLOD scores) is used to indicate the most likely number of clusters [73]. Based on this analysis, we re-reran the iterative reallocation procedure for the most likely *K* and plotted the mean LLOD score against geographically ordered sites as a means of identifying admixture levels between genetic clusters.

For all three methods of genetic cluster identification, we tested whether cluster assignment was valid by quantifying the number of individuals within each sample that were allocated to each cluster, and then building an *r* × *c* contingency table where *r* is the number of genetic clusters and *c* is the number of sample sites. We then tested for random allocation to the genetic clusters across empirical samples using a likelihood-ratio test with Williams’ correction with the null hypothesis that cluster assignments were random across sampled sites. A rejection of the null hypothesis indicated that that cluster composition was unlikely to be random across the samples, and that cluster assignments were therefore valid [74]. In addition, given that most clustering techniques assume that genotypic proportions within each cluster are in HWE and at linkage equilibrium, we tested identified clusters for compliance with these assumptions as suggested by Guillot et al. [66]. To test whether cluster assignment was independent of subspecific mtDNA clade membership we could not simply test for an association, as cluster assignment was confounded by spatial variation in the distribution of mtDNA clades. Therefore, we compiled cluster and clade membership for individuals in each of the four sites that contained members of more than one mtDNA clade (see Results, Fig 1a), and performed a likelihood-ratio test to determine whether cluster assignments were independent of subspecific clade membership within heterogeneous populations.

The level of genetic differentiation among pre-defined sites and an alternative grouping based on subspecific clade membership, where individuals were classified as belonging to the *M*. *l*. *alascensis*, *M*. *l*. *carissima*, or *M*. *l*. *lucifugus* clades based on the mtDNA phylogenetic analysis, was determined by calculating pairwise distance measures, including *F*_ST_ [75], and two measures independent of the amount of within-population diversity: Jost’s D [76], and *G*”_ST_ [77]. Differences in the magnitude of pairwise distance measures among groups of sites was tested using 10,000 permutations in GENODIVE (*G”*_ST_ and Jost’s D) and FSTAT (*F*_ST_). As with mtDNA, we performed an AMOVA on microsatellite genotypes using ARLEQUIN, and subsequently performed nested AMOVAs by grouping sites with low *F*_ST_ values to identify the grouping that maximized among-group variation and minimized among-site/within-group variation.

### Isolation by distance

There is considerable evidence to suggest that, regardless of the algorithm employed, clustering methods are confounded by the presence of isolation by distance (IBD), such that consistent clinal genetic variation may be misinterpreted by clustering algorithms as the presence of distinct clusters even though there is no true barrier to gene flow [66,78–80]. We therefore tested for IBD in mitochondrial and nuclear DNA both globally (including all sampled locations) and within identified clusters (for microsatellite data only). We conducted a Mantel test comparing standardized genetic distance [*F*_ST_/(1-*F*_ST_)] and the natural log of geographic distance [81] using the IBD Web Service [82]. To calculate between-site geographic distances, polylines were constructed from X,Y coordinates in ArcGIS 10.1. The geodesic distance of these polylines was calculated using the “Shape.length@meters” command. For the microsatellites, we followed the recommendations of Guillot et al. [66]: we plotted genetic distance (*F*_ST_) against geographic distance while differentiating between data points for site pairs that belonged to the same genetic cluster and data points for site pairs belonging to different clusters. If clusters are real, then for any given geographic distance, genetic distance between site pairs in different clusters should consistently be greater than distance between site pairs falling within the same cluster (e.g. [83,84]). In addition, as suggested by Meirmans [80], we performed a partial Mantel test to investigate the association between the matrix of genetic distances (*F*_ST_ and *G”*_ST_) and a matrix of cluster membership for the microsatellite data, with the matrix of geographical distances as a covariate. If there is not a relationship between genetic distance and cluster membership after controlling for geographic distance, then cluster membership is likely confounded by IBD. Given two main clusters (see Results), we coded cluster membership in two ways: a) both members of a sampling site pair belonging to the same cluster (1) or not (2); or b) both members of a sampling site pair belonging to a western cluster (1), both belonging to an eastern cluster (2), or sample sites belong in different clusters (3). Partial Mantel tests were carried out in PASSaGE software and significance was assessed with 10,000 permutations [85].

## Results

### Genetic diversity

We observed 148 unique haplotypes characterized by 104 segregating sites among the 617 individuals sequenced. The number of haplotypes per site ranged from 2–13 (mean: 7.8), and the number of haplotypes unique to a site ranged from 1–11 (mean: 4.5; Table 1). There was no significant difference in nucleotide diversity (*π*) between sites east versus west of the Great Plains-Rocky Mountains transition (Mann-Whitney U-Test; Mean East: 0.00, West: 0.01, *P* = 0.829), or between sites in states that were positive or negative for WNS as of the 2012–2013 winter season (Mann-Whitney U-Test; WNS-Neg: 0.01, WNS-Pos: 0.00, *P* = 0.132). However, sites west of the Great Plains-Rocky Mountains transition had significantly lower haplotype diversity than those east of the boundary (Mann-Whitney U-Test; East: 0.80, West: 0.56, *P* = 0.002), and WNS-free sites as of 2012 also had significantly lower haplotype diversity than WNS-affected sites (Mann-Whitney U-Test; WNS-Neg: 0.65, WNS-Pos: 0.81, *P* = 0.015), although this latter result is likely confounded by the high proportion of western sites in the WNS-Neg group.

Although we originally typed 11 microsatellite loci, two loci (E24 and COTO_G02_H10) had high null allele frequencies and were dropped from further analyses. The remaining nine loci all met HWE expectations and were unlinked. Mean observed and expected heterozygosities were high (0.876 and 0.897, respectively), as was the mean number of alleles per locus (13.9) and allelic richness (11.9), although private allelic richness was low (0.23; Table 1; see S4 Table for diversity statistics for each locus). Comparing sample-level measures of genetic diversity among identified clusters and among samples in WNS-positive and WNS-negative states or provinces revealed no significant differences in observed or expected heterozygosity, allelic richness, or *F*_IS_ (*P* > 0.05 in all cases based on permutation tests).

### Differentiation of *M*. *lucifugus* subspecies

**Fig 2:**
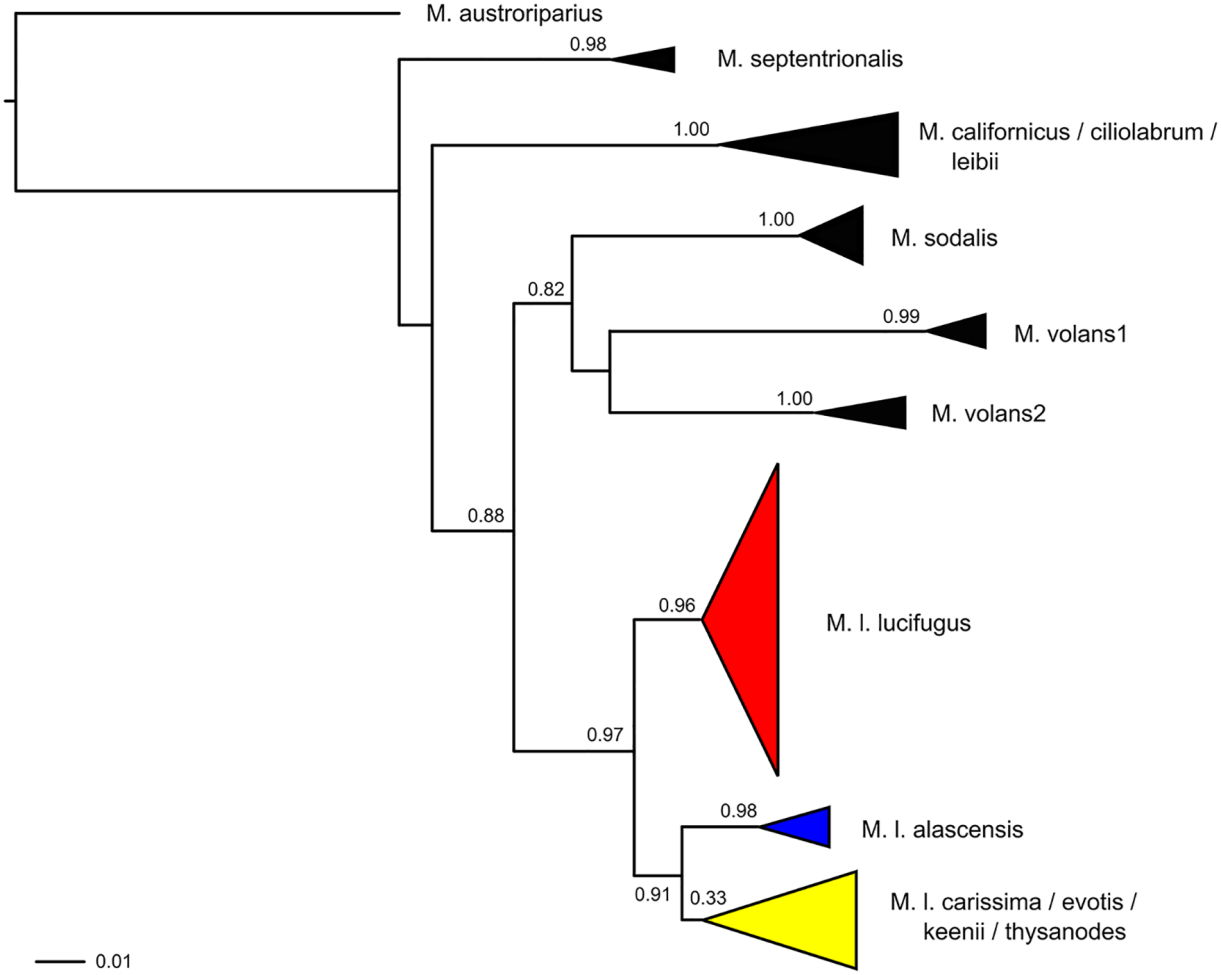
Phylogenetic tree showing relationships between Nearctic *Myotis* (sensu [58]), and the presence of three distinct clades within *M*. *lucifugus*, based on maximum likelihood analysis of partial COI sequences in PhyML 3.0. A member of the Neotropical *Myotis* clade (*M*. *austroriparius*) was included as the outgroup. Leaves are collapsed to highlight well-supported clades, and the vertical dimension of the triangles is proportional to the number of samples included. SH-like branch support values are provided for all major clades. Clades containing *M*. *lucifugus* are designated by the specific abbreviation followed by the subspecies name (e.g., *M*. *l*. *lucifugus* refers to the nominal subspecies). Note that one clade (including *M*. *l*. *carissima*) also contains members of other species (including *M*. *evotis*, *M*. *keenii*, and *M*. *thysanodes*) as previously described [32].

Phylogenetic analysis of the mtDNA COI sequences confirmed the existence of three clades within little brown bats roughly corresponding to previously defined subspecies (Fig 2). Following Carstens and Dewey [32], we refer to these clades as *M*. *l*. *lucifugus*, *M*. *l*. *carissima*, and *M*. *l*. *alascensis* (hereafter lucifugus, carissima, and alascensis, respectively). The carissima clade had three other species (*M*. *evotis*, *M*. *keenii*, *M*. *thysanodes*) nested within it, as previously described [32]. We have focused on *M*. *lucifugus sensu stricto* here. Addressing the taxonomic relationships among *M*. *lucifugus*, *M*. *evotis*, *M*. *keenii*, and *M*. *thysanodes* is beyond the scope of this paper; therefore we ignored the presence of these additional taxa in the rest of our analyses. For the most part, clades were geographically restricted and followed previously defined geographic ranges (Fig 1a). Almost all of the sampled sites east of the Great Plains-Rocky Mountains transition (with the exception of the Alberta-South site) were composed entirely of individuals classified as lucifugus. The carissima clade was restricted to mountainous but not coastal regions of the west and a portion of the western plains (in southern Alberta). The alascensis clade was found along the Pacific Coast and coastal mountain ranges (Fig 1a). However, individuals in the lucifugus clade were also sampled at locations west of the Great Plains-Rocky Mountains transition, including the British Columbia-South (BC-S), Wyoming (WY), and Washington (WA) samples. The Alberta-South (AB-S) sample east of the Great Plains-Rocky Mountains transition also contained both lucifugus and carissima haplotypes (see also [33]). No alascensis haplotypes were found at the BC-S site, although range maps place this site in that subspecies’ range; furthermore, the Alaska (AK) sample clustered with the alascensis clade, even though it fell within the described range of the lucifugus clade.

### Spatial patterns of population genetic structure

#### Mitochondrial DNA

AMOVA analysis considering all samples as a single group revealed high levels of differentiation (Φ_ST_ = 0.721). We iteratively grouped sites with low pairwise Φ_ST_ values to determine the best arrangements of sites that maximized ‘among-group’ and minimized ‘among-site/within-group’ variation in the AMOVA framework. Most samples were highly divergent from all others (76% of pairwise comparisons had Φ_ST_ > 0.2, and 62% were > 0.5; S5 Table and Fig 1b), but we identified two groups of sites (one in the central United States and Canada east of the Rocky Mountains, and one in eastern North America) within which divergence was low (Φ_ST_ = -0.019–0.130; Fig 1b). After grouping these sites together in an AMOVA analysis, among-group variation (Φ_CT_) accounted for 73.5% of variation in haplotype frequencies, and among-site/within-group variation accounted for 1.4%.

#### Microsatellite DNA

**Fig 3:**
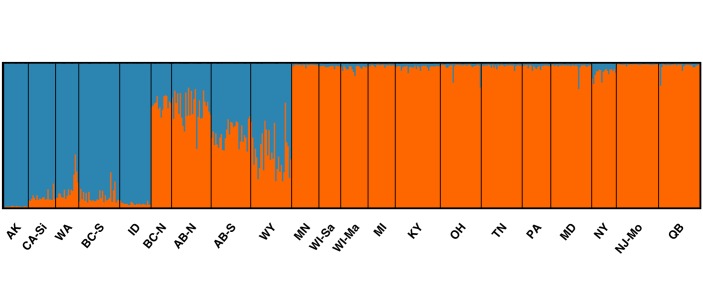
Proportional membership (Q) of *M*. *lucifugus* to genetic clusters for *K* = 2 estimated using STRUCTURE with sampling location as prior information. Each bar is a single individual, sampled populations are delineated by black lines and are ordered by geographical sampling location from west to east. Colors distinguish genetic clusters (blue for proportional membership in the western cluster, orange for proportional membership in the eastern cluster).

**Fig 4:**
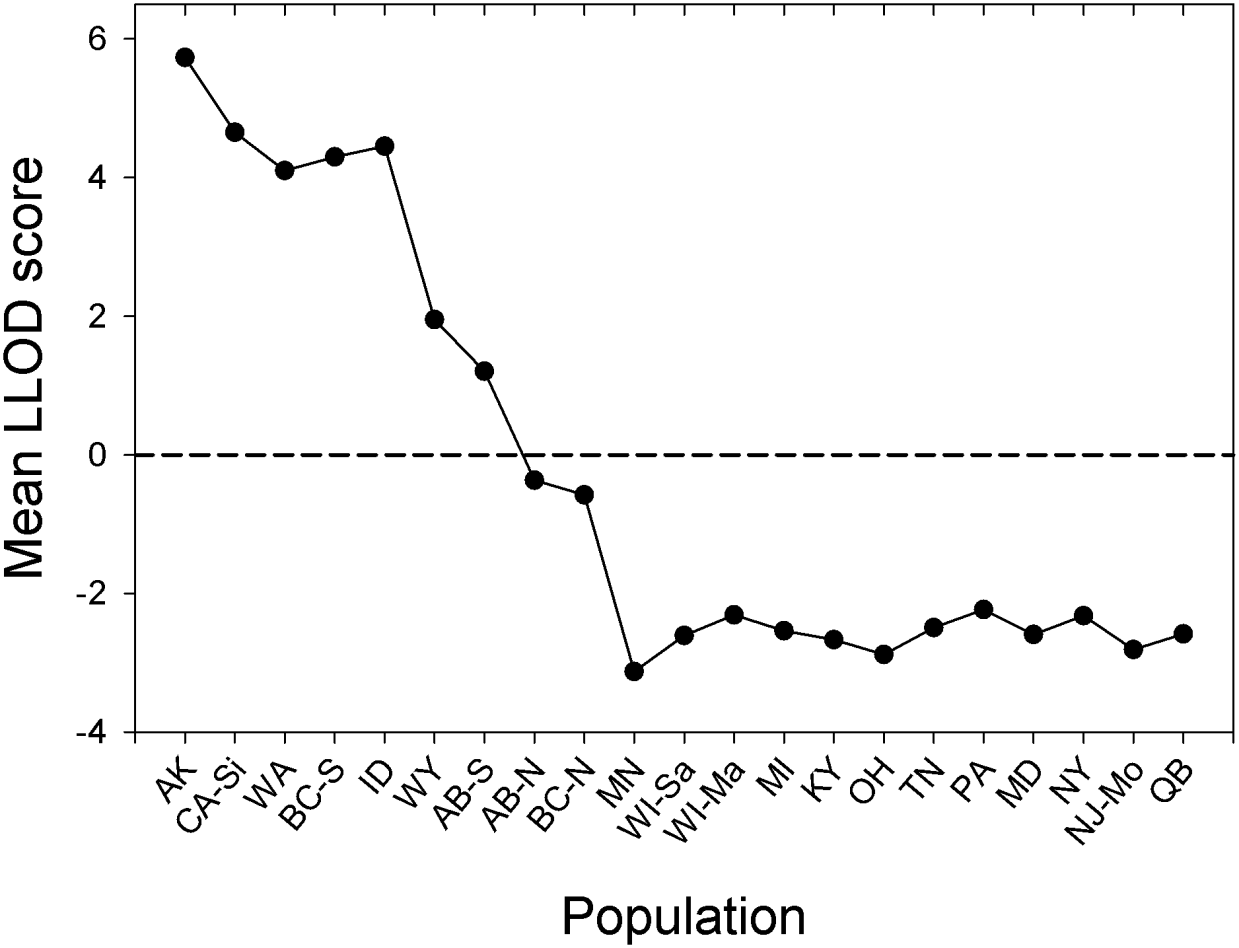
Mean log-likelihood difference (LLOD) between two genetic clusters obtained by FLOCK along a series of geographically ordered sites from west to east. For presentation, populations in the Great Plains-Rocky Mountains transition zone (BC-N AB-N, AB-S, and WY) are ordered by LLOD to demonstrate the transition among clusters.

All clustering methods employed [Bayesian clustering (STRUCTURE), repeated reallocation (FLOCK), and *K* means clustering (GENODIVE)] identified *K* = 2 as the most likely number of genetic clusters, roughly corresponding to clusters east versus west of the Great Plains-Rocky Mountains transition, and not corresponding to subspecies affiliation (Figs 3 and 4). However, four sites in the transition, namely British Columbia-North (BC-N), Alberta-North (AB-N), AB-S, and WY had intermediate Q (STRUCTURE) or LLOD (FLOCK) values indicative of admixture, and there was a clear gradation in this region between genetic clusters (Figs 3 and 4). On average, the BC-N and AB-N sites had higher proportional membership with the eastern cluster, and AB-S and WY sites with the western cluster, and therefore they were grouped accordingly in all subsequent analyses. To test the validity of the most likely number of clusters identified using each of the three algorithms, we performed contingency table analyses with the null hypothesis that cluster assignments were random with respect to sampling sites. In all cases we rejected the null hypothesis (STRUCTURE: *G* = 521.5; FLOCK: *G* = 422.6; GENODIVE: *G* = 77.1; df = 20 and *P* < 0.001 in all cases) and concluded that clusters were valid. However, all clusters identified by the three algorithms failed to meet HWE expectations (*P* < 0.05 in all cases). Across the four sites (WA, BC-S, AB-S, and WY) containing both carissima and lucifugus mtDNA haplotypes (setting aside the 3 alacensis-type individuals in WA), cluster membership was independent of subspecies affiliation for the clusters defined by FLOCK (*Χ* ^2^ = 1.65, *P* = 0.1990) and GENODIVE (*Χ* ^2^ = 0.22, *P* = 0.6390), but was not for STRUCTURE (*Χ* ^2^ = 5.65, *P* = 0.0175).

AMOVA analysis of microsatellite genotypes indicated weak but significant population structure (global *F*_ST_ = 0.0161, *P* < 0.001; proportion of variation within sites = 0.984). The grouping of sites that maximized among-group variation and minimized among-site/within-group variation included a group containing AK only, a western group of samples (California-Siskiyou (CA-Si), WA, BC-S, Idaho (ID), and WY), and an eastern group containing all other samples (variation among groups = 2.72%, *P* < 0.001; variation among sites within groups = 0.41%, *P* < 0.001). Generally, *F*_ST_ values between Alaska and all other sites were high and significant (0.049–0.089; S6 Table). *F*_ST_ values between sites in the western and eastern groups ranged from 0.004–0.045, values between sites within the western group ranged from 0.002–0.018, and values between sites within the eastern group ranged from -0.005–0.013 (S6 Table). An AMOVA grouping individuals based on their subspecific membership similarly indicated weak but significant population structure (global *F*_ST_ = 0.022, *P* < 0.001, proportion of variation within subspecies = 0.978). The maximum pairwise *F*_ST_ was between alascensis and lucifugus (0.0270, *P* < 0.001), the minimum was between alascensis and carissima (0.0141, *P* < 0.001), and differentiation between carissima and lucifugus was intermediate (0.0200, *P* < 0.001).

Genetic distance measures were higher among sites within the western group than among sites within the eastern group, and permutation tests approached significance at the *P* < 0.05 level (*F*_ST_: West: 0.023, East: 0.002, *P* = 0.0610; *G”*_ST_: West: 0.220, East: 0.016, *P* = 0.057; Jost’s D: West: 0.202, East: 0.014, *P* = 0.057). These results were largely driven by the inclusion of the AK site in the western group, and if this site was removed, genetic distance measures were still consistently higher within the west but not significantly different between groups (*F*_ST_: West: 0.007, East: 0.002, *P* = 0.361; *G”*_ST_: West: 0.088, East: 0.016, *P* = 0.273; Jost’s D: West: 0.081, East: 0.014, *P* = 0.274).

#### Isolation by distance

**Fig 5:**
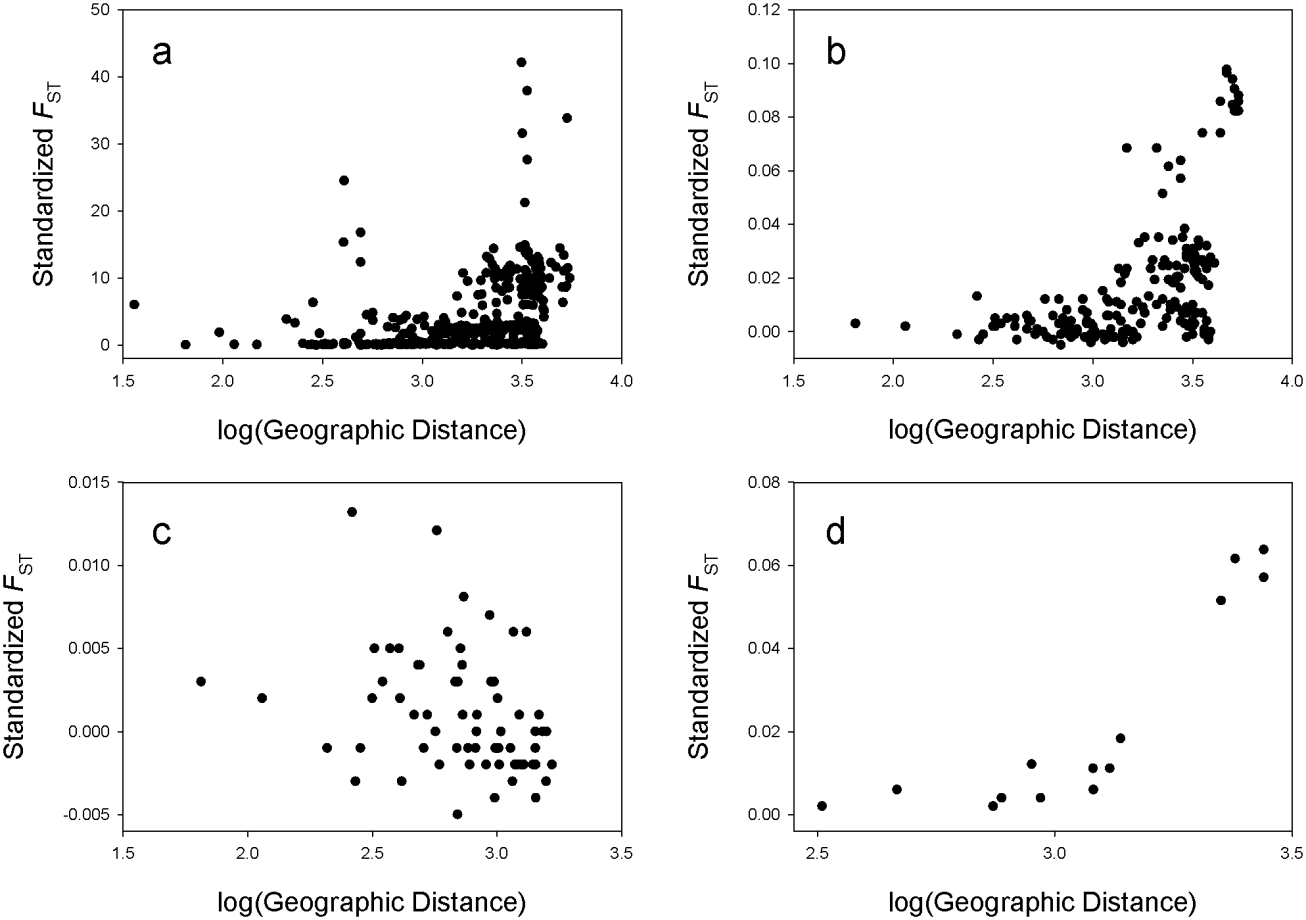
Standardized genetic distance [*F*_ST_/(1- *F*_ST_)] plotted against the logarithm of geographic distance including all sampled populations for mtDNA (a), and microsatellites (b), and for the eastern (c) and western (d) population clusters based on microsatellites.

To test for isolation by distance we performed Mantel tests on the logarithm of geographic distance and standardized genetic distance [*F*_ST_/(1-*F*_ST_)]. There were clear signals of IBD for both mitochondrial (*r* = 0.346, *P* < 0.0001; Fig 5a) and microsatellite DNA (*r* = 0.537, *P* < 0.0001; Fig 5b) across the range of little brown bats. Within identified clusters, there was no signal of IBD in the east (*r* = -0.307, *P* = 0.9925; Fig 5c), but there was within the west (*r* = 0.913, *P* = 0.0411; Fig 5d). To test if IBD in the west was disproportionately driven by the Alaska population, we re-ran the analysis with that site removed, and the signal of IDB remained (*r* = 0.880, *P* = 0.0069; S1 Fig).

**Fig 6:**
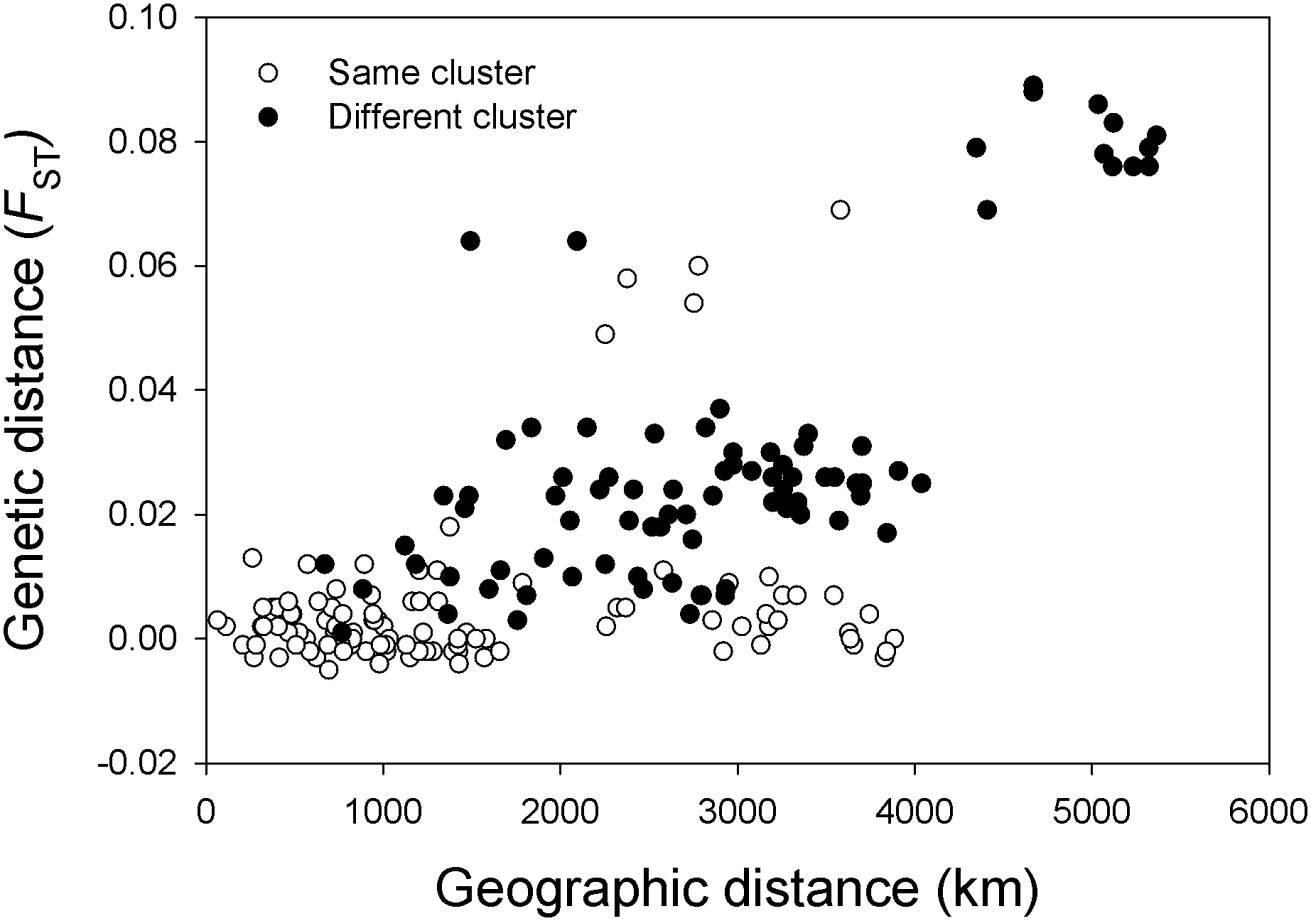
Pairwise genetic (*F*_ST_) based on microsatellites and geographic distance values highlighting population pairs within the same cluster (blue dots) and in different clusters (orange dots).

To assess the validity of clusters given the pattern of isolation by distance, we plotted geographic and genetic distance (*F*_ST_) based on microsatellites according to cluster membership (points identified separately for comparisons within the same cluster vs. in different clusters; Fig 6). There was no clear separation between site pairs in the same vs. different clusters, and low genetic distances for a given geographic distance were observed regularly for site pairs in different clusters. However, partial Mantel tests to examine the association between the matrix of genetic distances and a matrix of cluster membership for the microsatellite data, with the matrix of geographical distances as a covariate, were significant (*P* < 0.05 in all cases) regardless of coding scheme (see Methods) or genetic distance measure (*F*_ST_ or *G”*_ST_) used.

## Discussion

The emergence and spread of WNS has decimated bat populations in affected areas and raised the specter of extinction for *Myotis lucifugus*, *M*. *septentrionalis*, and other highly-affected bat species. Therefore, understanding population connectivity and possible barriers to disease transmission is vital to the ongoing management and conservation of affected species and the development of mitigation strategies to limit disease spread and associated mortality. Here, using a combined dataset of mtDNA sequences and nuclear microsatellite genotypes for little brown bats from across their range, we demonstrate considerable spatial variation in patterns of female dispersal and significant genetic variation between sites in eastern versus western portions of the range of little brown bats. Whether the observed variation is representative of discrete genetic clusters rather than isolation by distance is debatable (see below), but overall, it is clear that levels of nuclear genetic differentiation are low, and there is no evidence for any major barriers to nuclear gene flow across the range of little brown bats. However, some key spatial patterns emerge from our analyses, namely (1) patterns of mtDNA differentiation are highly variable, with high Φ_ST_ values between most sample pairs (including between all western samples, between western and eastern samples, and between some eastern samples), while low mitochondrial differentiation was observed within two groups of samples found in central and eastern regions of North America (shown in AMOVA and pairwise Φ_ST_ analyses; Fig 1b); (2) the site from Alaska is highly differentiated from all others in our study (shown in AMOVA and pairwise *F*_ST_ analyses); and (3) western sites are characterized by significant isolation by distance based on microsatellites, while those in the east are not. These data raise the possibility that the current patterns of spread of *Pd* observed in eastern North America may not apply to the entire range of the little brown bat, and that there may be broad-scale spatial variation in the risk of WNS transmission and occurrence if the disease continues to spread west.

The presence of isolation by distance is a major confounding factor when examining levels of genetic structure among populations of widespread species such as little brown bats, because clinal variation may be interpreted as the presence of discrete clusters even in the absence of barriers to gene flow [66,78,80]. All clustering methods we employed on our microsatellite data identified the presence of two genetic clusters, roughly dividing sites east vs. west of the Great Plains-Rocky Mountains transition. However, we also observed a strong pattern of isolation by distance, indicating that these observed clusters may be an artifact of dispersal limitation and clinal variation across the very large range of this species. Additional analyses to test the validity of clusters provided mixed results. On the one hand, genetic distances between eastern and western sites were relatively low (the best grouping in the AMOVA explained only 2.7% of the variation in microsatellite allele frequencies), a Mantel test showed no clear separation between site pairs in the same versus different clusters (Fig 6), and identified clusters failed to meet HW expectations, suggesting that clusters did not represent genetically panmictic populations (cf. [66]). On the other hand, partial Mantel tests controlling for geographic distance revealed a significant correlation between cluster membership and genetic distance, suggesting that western and eastern clusters were differentiated despite the signal of isolation by distance (cf. [80]).

What is clear from these data is that there is significant genetic variation among samples from east to west across the range of little brown bats (although whether they represent discrete genetic clusters rather than isolation by distance is debatable), but levels of nucDNA genetic differentiation were relatively low, and there was no unambiguous evidence for any major barriers to gene flow that might severely restrict the spread of WNS. Gene flow in temperate bats is mediated through the permanent dispersal of individuals and the exchange of genes at mating congregations during swarming and hibernation. The lack of isolation by distance and low levels of nucDNA differentiation among sites in eastern North America is concordant with the continuous spread of WNS from its origin in New York, and indicates that gene flow via mating has occurred over wide geographic areas. Furthermore, the disease has passed, or is currently passing, through regions in which there are low levels of mtDNA differentiation among sites (one group in the eastern United States and one group in the central United States and Canadian provinces; Fig 1b). Most temperate bats are characterized by relatively high levels of female philopatry and male-biased dispersal, resulting in significant matrilineal genetic structuring of populations (e.g. [35,86–88]). However, our data suggest that the exchange of females among populations across large portions of the range of little brown bats is a non-trivial source of gene flow that may be contributing to the spread of WNS, and are consistent with similar inferences of female dispersal among populations over smaller spatial scales in little brown bats [6,33,35,42] and other bat species [38,89] based on mtDNA. In addition, these data are consistent with banding data showing extensive movements by individual little brown bats of both sexes over hundreds of kilometers between summer roosts, swarming sites, and hibernacula within and between years in central Canada [27,42]. Banding studies of little brown bats in other parts of their range would help to resolve whether the observed differences are better explained by historical demography or current gene flow. The high levels of observed mtDNA differentiation in other portions of the range, particularly in the west, suggest important spatial variation in female dispersal patterns, and highlight the need to consider permanent movements of both males and females and incorporate regional variation in dispersal rates and distances in models of WNS transmission dynamics.

Given the lack of major physiographic barriers east of the Rocky Mountains and the high levels of gene flow we inferred, it is likely that WNS will continue to expand its range across eastern North America. Current models of disease spread indicate that WNS exhibits characteristics of an expanding epizootic wherein relatively distant sites have lower infection risk, but over time infection rates increase and the effect of distance diminishes as the disease ‘fills in’ behind the expansion front [19,20]. However, these models are based on data from eastern portions of the range where gene flow is high. Even within this region the rate of spread may be restricted by consistent spatial variation in above-ground or cave microclimates that may limit the survival and growth of *Pd* (cf. [20,21]), or by spatial variation in topography or land use that limits movements and dispersal by bats [6]. In Pennsylvania, for example, topographical features such as the Appalachian high plateau and the Allegheny Front escarpment may have influenced seasonal migration patterns of female bats, thereby limiting matrilineal gene flow and disease transmission rates among populations [6]. Indeed, two genetically distinct populations of wintering colonies were observed on either side of the Allegheny Front [6]; hibernating colonies of little brown bats located on the western Appalachian high plateau were infected with WNS 1–2 years later than colonies in the central mountainous and eastern lowland regions of the state [16]. Thus, topographic or climatic variation may slow the spread of WNS through some areas by limiting population connectivity of the host or the survival and growth of *Pd*, and may explain some of the observed spatial variation to date in the rate and direction of WNS spread through eastern North America.

Our genetic data indicating lower levels of population connectivity in the west suggest that if WNS reaches western populations, the rate of disease spread may decline. The high mtDNA Φ_ST_ values among western populations (S5 Table) indicate that female movements are highly restricted relative to eastern populations. Overall levels of nucDNA gene flow among western sites were reduced relative to the east, and western sites were characterized by isolation by distance based on microsatellites while eastern sites were not. These results may, in part, be related to the greater topographical and ecological heterogeneity in the west, which includes multiple mountain ranges, plateaus, basins, and coastal lowlands, and which has been implicated in recurrent phylogeographic patterns in a wide variety of other taxa [90]. Hibernation behavior is poorly characterized for western North American little brown bat populations. All known large hibernating populations (>10,000 individuals) are described from eastern North America, and identified hibernacula in the mountainous west typically have lower census sizes than many hibernacula in the east. The high physiographic variation in the mountainous west may limit population connectivity and the scale of bat movements, and the high density of mines and caves in many regions in the west may result in smaller and more diffuse hibernating colonies relative to eastern North America. Comparative data on connectivity between summer and winter sites (as in [27]) are urgently required to quantify spatial and temporal patterns of movement of little brown bats in the western portion of their range and to predict potential rates of WNS transmission. Further, the most distant population we sampled (in Alaska) was by far the most divergent from all other populations, and we require much more dense sampling in the western portion of the range of little brown bats to determine if any other populations are equally or more isolated and hence may have reduced contact rates with other regional populations.

The spatial variation in population connectivity we observed was largely independent of subspecific affiliation. Phylogenetic analysis revealed the presence of three divergent lineages based on mtDNA (corresponding to previously defined subspecies *M*. *l*. *alascensis*, *M*. *l*. *carissima* and *M*. *l*. *lucifugus*; as in [32]), with the notable finding of multiple lineages at the same sampling locations in southern British Columbia, southern Alberta (as in [33]), and Wyoming. Although Carstens and Dewey [32] provided some support from mtDNA and nuclear introns for discrete evolutionary lineages within *M*. *lucifugus*, we found that cluster membership based on microsatellites was independent of subspecific affiliation, and we estimated low levels of nucDNA differentiation among subspecies (*F*_ST_ = 0.022 in AMOVA analysis). Our observed discrepancy between mtDNA and nucDNA signals may be due in part to homoplasy, particularly at rapidly-evolving microsatellite loci. Alternatively, incomplete lineage sorting might be producing discordant patterns among loci, particularly for a species with a relatively large effective population size (*N*_e_ ≈ 400,000 based on Carstens and Dewey’s [32] estimate of θ for *M*. *lucifugus*) and relatively recent divergence (divergence from western *Myotis* sp. approximately 1–1.5 Mya [32]). A third alternative is that our finding of lower differentiation at nucDNA compared to mtDNA is that patterns at mtDNA may reflect genetic differentiation that evolved and were reinforced as populations used distinct glacial refuges (as in [34]), while patterns at nucDNA reflect secondary contact particularly mediated via male gene flow.

Our data show extensive spatial variation in levels of connectivity among little brown bat populations and provide valuable information for understanding past and future patterns of WNS spread. However, the use of genetic methods to infer patterns of transmission assumes that patterns of gene flow are indicative of the movement of infectious individuals, and we must recognize that the risk of disease transmission may be higher than genetic data may indicate because there may be more contacts among infected and susceptible individuals, including among members of multiple species, than just those that lead to gene flow. Urgent research is required to determine how and when individual bats may be exposed to *Pd* spores, and how contacts of varying durations and seasonal timings influence the risk of WNS transmission. Ultimately we need to learn whether brief contacts during mating can result in transfer of spores leading to infection or whether permanent dispersals are driving transmission. The usefulness of our genetic data on little brown bats also rests on the assumption that intraspecific transmission dynamics outweigh the impact of cross-species transmission, given that multiple sympatric bat species are affected by WNS. This assumption may be justified, as post-WNS population declines of affected bat species are not influenced by population sizes of other affected, cohabiting bat species [21], but research is required to assess how often cross-species transmission may take place and how the rate of introduction of infective propagules to environmental reservoirs is influenced by multiple species cohabiting the same hibernaculum.

In conclusion, this study identified high levels of genetic variation among populations of little brown bats across their range, and mitochondrial DNA sequences revealed considerable spatial variation in patterns of female dispersal. Overall levels of nuclear genetic differentiation among *M*. *lucifugus* populations are low, and we did not identify any major barriers to gene flow across their range. However, levels of genetic differentiation at both mtDNA and microsatellites are significantly higher among populations to the west of the Great Plains-Rocky Mountains transition, suggesting that the current pattern of spread of WNS and risk of transmission of *Pd* observed in eastern North America may not apply to the entire range of the little brown bat.

## Supporting Information

S1 Fig Standardized genetic distance [*F*_ST_/(1 –*F*_ST_)] for microsatellites plotted against the logarithm of geographic distance for the western population cluster with the Alaska population removed. (DOCX)

S1 File Data sources and permissions used to construct Fig 1. (PDF)

S1 Table List of *Myotis lucifugus* specimens included in analyses. (DOCX)

S2 Table Locus information for 11 microsatellites used to amplify *M*. *lucifugus*. (DOCX)

S3 Table List of specimens (all in the genus *Myotis*), year of collection, sampling localities, and Genbank accession numbers for COI sequences used in phylogenetic analysis. (DOCX)

S4 Table Diversity of microsatellite loci, including observed (*H*_O_) and expected heterozygosity (*H*_E_), number of alleles (*N*_A_), allelic richness (*AR*) and the inbreeding coefficient (*F*_IS_). (DOCX)

S5 Table Pairwise Φ_ST_ values among populations based on mtDNA COI sequences. (DOCX)

S6 Table Pairwise *F*_ST_ (lower diagonal) and Jost’s *D* (upper diagonal) based on nucDNA microsatellites. (DOCX)

## References

[1] Biek R, Real LA (2010) The landscape genetics of infectious disease emergence and spread. Mol Ecol 19: 3515–3531. 10.1111/j.1365-294X.2010.04679.x 20618897 PMC3060346

[2] Real LA, Biek R (2007) Spatial dynamics and genetics of infectious diseases on heterogeneous landscapes. J R Soc Interface 4: 935–948. 17490941 10.1098/rsif.2007.1041 PMC2074889

[3] Cullingham CI, Merrill EH, Pybus MJ, Bollinger TK, Wilson GA, Coltman DW (2010) Broad and fine-scale genetic analysis of white-tailed deer populations: estimating the relative risk of chronic wasting disease spread. Evol Appl 4: 116–131. 10.1111/j.1752-4571.2010.00142.x 25567957 PMC3352516

[4] Vollmer SA, Bormane A, Dinnis RE, Seelig F, Dobson ADM, Aanensen DM, et al (2011) Host migration impacts on the phylogeography of Lyme Borreliosis spirochaete species in Europe. Environ Microbiol 13: 184–192. 10.1111/j.1462-2920.2010.02319.x 20722696

[5] Gray RR, Salemi M (2012) Integrative molecular phylogeography in the context of infectious diseases on the human-animal interface. Parasitology 139: 1939–1951. 10.1017/S0031182012001102 22931895

[6] Miller-Butterworth CM, Vonhof MJ, Rosenstern J, Turner G, Russell A (2014) Genetic structure of little brown bats (*Myotis lucifugus*) corresponds with spread of white-nose syndrome among hibernacula. J Hered 105: 354–364. 10.1093/jhered/esu012 24591103

[7] Blehert DS, Hicks AC, Behr M, Meteyer CU, Berlowski-Zier BM, Buckles EL, et al (2009) Bat white-nose syndrome: An emerging fungal pathogen? Science 323: 227 10.1126/science.1163874 18974316

[8] Lorch JM, Meteyer CU, Behr MJ, Boyles JG, Cryan PM, Hicks AC, et al (2011) Experimental infection of bats with *Geomyces destructans* causes white-nose syndrome. Nature 480: 376–378. 10.1038/nature10590 22031324

[9] Turner GG, Reeder DM, Coleman JTH (2011) A five-year assessment of mortality and geographic spread of White-nose Syndrome in North American bats and a look to the future. Bat Res News 52: 13–27.

[10] Meteyer CU, Buckles EL, Blehert DS, Hicks AC, Green DE, Shearn-Bochsler V, et al (2009) Histopathologic criteria to confirm white-nose syndrome in bats. J Vet Diagn Invest 21: 411–414. 19564488 10.1177/104063870902100401

[11] Cryan PM, Meteyer CU, Boyles JG, Blehert DS (2010) Wing pathology of white-nose syndrome in bats suggests life-threatening disruption of physiology. BMC Biol 8: 135 10.1186/1741-7007-8-135 21070683 PMC2984388

[12] Willis CKR, Menzies AK, Boyles JG, Wojciechowski MS (2011) Evaporative water loss is a plausible explanation for mortality of bats from white-nose syndrome. Integr Comp Biol 51: 364–373. 10.1093/icb/icr076 21742778

[13] Cryan PM, Meteyer CU, Blehert DS, Lorch JM, Reeder DM, Turner GG, et al (2013) Electrolyte depletion in white-nose syndrome bats. J Wildl Dis 49: 398–402. 10.7589/2012-04-121 23568916

[14] Reeder DM, Frank CL, Turner GG, Meteyer CU, Kurta A, Britzke ER, et al (2012) Frequent arousal from hibernation linked to severity of infection and mortality in bats with white-nose syndrome. PLoS One 7: e38920 10.1371/journal.pone.0038920 22745688 PMC3380050

[15] Verant ML, Meteyer CU, Speakman JR, Cryan PM, Lorch JM, Blehert DS (2014) White-nose syndrome initiates a cascade of physiologic disturbances in the hibernating bat host. BMC Physiology 14: e10. 10.1186/s12899-014-0010-4 PMC4278231 25487871

[16] U.S. Fish and Wildlife Service (2014) Current WNS Occurrence Map. Map by C. Butchkoski. Available: http://whitenosesyndrome.org/about/where-is-it-now

[17] U.S. Fish and Wildlife Service (2012) North American bat death toll exceeds 5.5 million from white-nose syndrome. U.S. Fish and Wildlife Service News Release.

[18] Frick WF, Pollock JF, Hicks AC, Langwig KE, Reynolds DS, Turner GG, et al (2010) An emerging disease causes regional population collapse of a common North American bat species. Science 329: 679–682. 10.1126/science.1188594 20689016

[19] Wilder AP, Frick WF, Langwig KE, Kunz TH (2011) Risk factors associated with mortality from white-nose syndrome among hibernating bat colonies. Biology Lett 7: 950–953. 10.1098/rsbl.2011.0355 PMC3210657 21632616

[20] Thogmartin WE, King RA, Szymanski JA, Pruitt L (2012) Space-time models for a panzootic in bats, with a focus on the endangered Indiana bat. J Wildl Dis 48: 876–887. 10.7589/2011-06-176 23060489

[21] Langwig KE, Frick WF, Bried JT, Hicks AC, Kunz TH, Kilpatrick AM (2012) Sociality, density-dependence and microclimates determine the persistence of populations suffering from a novel fungal disease, white-nose syndrome. Ecol Lett 15: 1050–1057. 10.1111/j.1461-0248.2012.01829.x 22747672

[22] Fenton MB, Barclay RMR (1980) Myotis lucifugus. Mamm Species 142: 1–8.

[23] Reid FA (2006) Mammals of North America. Boston: Houghton Mifflin Company.

[24] Davis WH, Hitchcock HB (1965) Biology and migration of the bat, *Myotis lucifugus*, in New England. J Mammal 46: 296–313.

[25] Thomas DW, Fenton MB, Barclay RMR (1979) Social behavior of the little brown bat, *Myotis lucifugus*. I. Mating behavior. Behav Ecol Sociobiol 6: 129–136.

[26] Rivers NM, Butlin RK, Altringham JD (2006) Autumn swarming behaviour of Natterer's bats in the UK: Population size, catchment area and dispersal. Biol Conserv 127: 215–226.

[27] Norquay KJO, Martinez-Nuñez F, Dubois JE, Monson KM, Willis CKR (2013) Long-distance movements of little brown bats (*Myotis lucifugus*). J Mammal 94: 506–515.

[28] Kerth G, Kiefer A, Trappmann C, Weishaar M (2003) High gene diversity at swarming sites suggest hot spots for gene flow in the endangered Bechstein's bat. Conserv Genet 4: 491–499.

[29] Veith M, Beer N, Kiefer A, Johannesen J, Seitz A (2004) The role of swarming sites for maintaining gene flow in the brown long-eared bat (*Plecotus auritus*). Heredity 93: 342–349. 15241447 10.1038/sj.hdy.6800509

[30] Furmankiewicz J, Altringham J (2007) Genetic structure in a swarming brown long-eared bat (*Plecotus auritus*) population: evidence for mating at swarming sites. Conserv Genet 8: 913–923.

[31] Wilson DE, Reeder DM (2005) Mammal species of the world: A taxonomic and geographic reference, third edition Baltimore: Johns Hopkins University Press.

[32] Carstens BC, Dewey TA (2010) Species delimitation using a combined coalescent and information-theoretic approach: An example from North American *Myotis* bats. Systematic Biol 59: 400–414. 10.1093/sysbio/syq024 20547777 PMC2885268

[33] Lausen CL, Delisle I, Barclay RMR, Strobeck C (2008) Beyond mtDNA: nuclear gene flow suggests taxonomic oversplitting in the little brown bat (*Myotis lucifugus*). Can J Zool 86: 700–713.

[34] Dixon MD (2011) Post-Pleistocene range expansion of the recently imperiled eastern little brown bat (*Myotis lucifugus lucifugus*) from a single southern refugium. Ecol Evol 1: 191–200. 10.1002/ece3.20 22393495 PMC3287298

[35] Dixon MD (2011) Population genetic structure and natal philopatry in the widespread North American bat *Myotis lucifugus* . J Mammal 92: 1343–1351.

[36] Bilgin R, Karatas A, Coraman E, Morales JC (2008) The mitochondrial and nuclear genetic structure of *Myotis capaccinii* (Chiroptera: Vespertilionidae) in the Eurasian transition, and its taxonomic implications. Zool Scr 37: 253–262.

[37] Kerth G, Petrov B, Conti A, Anastasov D, Weishaar M, Gazaryan S, et al (2008) Communally breeding Bechstein's bats have a stable social system that is independent from the postglacial history and location of the populations. Mol Ecol 17: 2368–2381. 10.1111/j.1365-294X.2008.03768.x 18429964

[38] Vonhof MJ, Strobeck C, Fenton MB (2008) Genetic variation and population structure in big brown bats (*Eptesicus fuscus*): is female dispersal important? J Mammal 89: 1411–1420.

[39] Bryja J, Kanuch P, Fornuskova A, Bartonicka T, Rehak Z (2009) Low population genetic structuring of two cryptic bat species suggests their migratory behaviour in continental Europe. Biol J Linn Soc 96: 103–114.

[40] Lack JB, Wilkinson JE, van den Bussche RA (2010) Range-wide population genetic structure of the pallid bat (*Antrozous pallidus*)—incongruent results from nuclear and mitochondrial DNA. Acta Chiropt 12: 401–413.

[41] Turmelle AS, Kunz TH, Sorenson MD (2011) A tale of two genomes: contrasting patterns of phylogeographic structure in a widely distributed bat. Mol Ecol 20: 357–375. 10.1111/j.1365-294X.2010.04947.x 21143331

[42] Burns LE, Frasier TR, Broders HG (2014) Genetic connectivity among swarming sites in the wide ranging and recently declining little brown bat (*Myotis lucifugus*). Ecol Evol 4: 4130–4149. 10.1002/ece3.1266 25505539 PMC4242565

[43] Gibbs HL, Dawson RJG, Hobson KA (2000) Limited differentiation in microsatellite DNA variation among northern populations of the yellow warbler: evidence for male-biased gene flow? Mol Ecol 9: 2137–2147. 11123625 10.1046/j.1365-294x.2000.01136.x

[44] Kimura M, Clegg SM, Lovette IJ, Holder KR, Girman DJ, Milá B, et al (2002) Phylogeographical approaches to assessing demographic connectivity between breeding and overwintering regions in a Nearctic-Neotropical warbler (*Wilsonia pusilla*). Mol Ecol 11: 1605–1616. 12207712 10.1046/j.1365-294x.2002.01551.x

[45] Jones KL, Krapu GL, Brandt DA, Ashley MV (2005) Population genetic structure in migratory sandhill cranes and the role of Pleistocene glaciations. Mol Ecol 14: 2645–2657. 16029467 10.1111/j.1365-294X.2005.02622.x

[46] Irwin DE, Irwin JH, Smith TB (2011) Genetic variation and seasonal migratory connectivity in Wilson's warblers (*Wilsonia pusilla*): species-level differences in nuclear DNA between western and eastern populations. Mol Ecol 20: 3102–3115. 10.1111/j.1365-294X.2011.05159.x 21689190

[47] Worthington Wilmer J, Barratt E (1996) A non-lethal method of tissue sampling for genetic studies of chiropterans. Bat Res News 37: 1–3.

[48] Hebert PDN, Cywinska A, Ball SL, DeWaard JR (2003) Biological identifications through DNA barcodes. Proc R Soc Lond Ser B-Biol Sci 270: 313–321. 10.1098/rspb.2002.2218 PMC1691236 12614582

[49] Ivanova NV, Dewaard JR, Hebert PDN (2006) An inexpensive, automation-friendly protocol for recovering high-quality DNA. Mol Ecol Notes 6: 998–1002.

[50] Katoh K, Standley DM (2013) MAFFT Multiple Sequence Alignment Software Version 7: Improvements in performance and usability. Mol Biol Evol 30: 772–780. 10.1093/molbev/mst010 23329690 PMC3603318

[51] Oyler-McCance SJ, Fike JA (2011) Characterization of small microsatellite loci isolated in endangered Indiana bat (*Myotis sodalis*) for use in non-invasive sampling. Conserv Genet Resour 3: 243–245.

[52] Trujillo RG, Amelon SK (2009) Development of microsatellite markers in *Myotis sodalis* and cross-species amplification in *M* . *grisescens*, *M*. *leibii*, *M*. *lucifugus*, and *M*. *septentrionalis* . Conserv Genet 10: 1965–1968.

[53] Castella V, Reudi M, Excoffier L, Ibanez C, Arlettaz R, Hausser J (2000) Is the Gibraltar Strait a barrier to gene flow for the bat *Myotis myotis* (Chiroptera: Vespertilionidae)? Mol Ecol 9: 1761–1772. 11091312 10.1046/j.1365-294x.2000.01069.x

[54] Piaggio AJ, Figueroa JA, Perkins SL (2009) Development and characterization of 15 polymorphic microsatellite loci isolated from Rafinesque's big-eared bat, *Corynorhinus rafinesquii* . Mol Ecol Resour 9: 1191–1193. 10.1111/j.1755-0998.2009.02625.x 21564872

[55] Piaggio AJ, Miller KEG, Matocq MD, Perkins SL (2009) Eight polymorphic microsatellite loci developed and characterized from Townsend's big-eared bat, *Corynorhinus townsendii* . Mol Ecol Resour 9: 258–260. 10.1111/j.1755-0998.2008.02243.x 21564620

[56] Librado P, Rozas J (2009) DnaSP v5: A software for comprehensive analysis of DNA polymorphism data. Bioinformatics 25: 1451–1452. 10.1093/bioinformatics/btp187 19346325

[57] Villesen P (2007) FaBox: an online toolbox for fasta sequences. Mol Ecol Notes 7: 965–968.

[58] Excoffier L, Laval G, Schneider S (2005) Arlequin ver. 3.0: An integrated software package for population genetics data analysis. Evol Bioinform Online 1: 47–50. PMC2658868 19325852

[59] Excoffier L, Smouse PE, Quattro JM (1992) Analysis of molecular variance inferred from metric distances among DNA haplotypes: application to human mitochondrial DNA restriction data. Genetics 131: 479–491. 1644282 10.1093/genetics/131.2.479 PMC1205020

[60] Guindon S, Dufayard J-F, Lefort V, Anisimova M, Hordijk W, Gascuel O (2010) New algorithms and methods to estimate maximum-likelihood phylogenies: Assessing the performance of PhyML 3.0. Syst Biol 59: 307–321. 10.1093/sysbio/syq010 20525638

[61] Stadelmann B, Lin L-K, Kunz TH, Ruedi M (2007) Molecular phylogeny of New World *Myotis* (Chiroptera, Vespertilionidae) inferred from mitochondrial and nuclear DNA genes. Mol Phylogent Evol 43: 32–48. 10.1016/j.ympev.2006.06.019 17049280

[62] Tamura K, Peterson D, Peterson N, Stecher G, Nei M, Kumar S (2011) MEGA5: Molecular evolutionary genetics analysis using maximum likelihood, evolutionary distance, and maximum parsimony methods. Mol Biol Evol 28: 2731–2739. 10.1093/molbev/msr121 21546353 PMC3203626

[63] Goudet J (1995) FSTAT (Version 1.2): A computer program to calculate F-statistics. J Hered 86: 485–486.

[64] Kalinowski ST (2005) HP-RARE 1.0: a computer program for performing rarefaction on measures of allelic richness. Mol Ecol Notes 5: 187–189.

[65] Meirmans PG (2012) AMOVA-based clustering of population genetic data. J Hered 103: 744–750. 10.1093/jhered/ess047 22896561

[66] Guillot G, Leblois R, Coulon A, Frantz AC (2009) Statistical methods in spatial genetics. Mol Ecol 18: 4734–4756. 10.1111/j.1365-294X.2009.04410.x 19878454

[67] Pritchard JK, Stephens M, Donnelly P (2000) Inference of population structure using multilocus genotype data. Genetics 155: 945–959. 10835412 10.1093/genetics/155.2.945 PMC1461096

[68] Falush D, Stephens M, Pritchard JK (2003) Inference of population structure using multilocus genotype data: linked loci and correlated allele frequencies. Genetics 164: 1567–1587. 12930761 10.1093/genetics/164.4.1567 PMC1462648

[69] Hubisz MJ, Falush D, Stephens M, Pritchard JK (2009) Inferring weak population structure with the assistance of sample group information. Mol Ecol Resour 9: 1322–1332. 10.1111/j.1755-0998.2009.02591.x 21564903 PMC3518025

[70] Evanno G, Regnaut S, Goudet J (2005) Detecting the number of clusters of individuals using the software STRUCTURE: a simulation study. Mol Ecol 14: 2611–2620. 15969739 10.1111/j.1365-294X.2005.02553.x

[71] Earl DA, vonHoldt BM (2012) STRUCTURE HARVESTER: a website and program for visualizing STRUCTURE output and implementing the Evanno method. Conserv Genet Resour 4: 359–361.

[72] Jakobsson M, Rosenberg NA (2007) CLUMPP: a cluster matching and permutation program for dealing with label switching and multimodality in analysis of population structure. Bioinformatics 23: 1801–1806. 17485429 10.1093/bioinformatics/btm233

[73] Duchesne P, Turgeon J (2012) FLOCK provides reliable solutions to the "number of populations" problem. J Hered 103: 734–743. 10.1093/jhered/ess038 22615162

[74] Duchesne P, Turgeon J (2009) FLOCK: a method for quick mapping of admixture without source samples. Mol Ecol Resour 9: 1333–1344. 10.1111/j.1755-0998.2009.02571.x 21564904

[75] Weir BS, Cockerham CC (1984) Estimating *F*-statistics for the analysis of population structure. Evolution 38: 1358–1370. 28563791 10.1111/j.1558-5646.1984.tb05657.x

[76] Jost L (2008) G_ST_ and its relatives do not measure differentiation. Mol Ecol 17: 4015–4026. 19238703 10.1111/j.1365-294x.2008.03887.x

[77] Meirmans PG, Hedrick PW (2011) Assessing population structure: *F* _*ST*_ and related measures. Mol Ecol Resour 11: 5–18. 10.1111/j.1755-0998.2010.02927.x 21429096

[78] Frantz AC, Cellina S, Krier A, Schley L, Burke T (2009) Using spatial Bayesian methods to determine the genetic structure of a continuously distributed population: clusters or isolation by distance? J Appl Ecol 46: 493–505.

[79] Schwartz M, McKelvey K (2009) Why sampling scheme matters: the effect of sampling scheme on landscape genetic results. Conserv Genet 10: 441–452.

[80] Meirmans PG (2012) The trouble with isolation by distance. Mol Ecol 21: 2839–2846. 10.1111/j.1365-294X.2012.05578.x 22574758

[81] Rousset F (1997) Genetic differentiation and estimation of gene flow from *F*-statistics under isolation by distance. Genetics 145: 1219–1228. 9093870 10.1093/genetics/145.4.1219 PMC1207888

[82] Jensen JL, Bohonak AJ, Kelley ST (2005) Isolation by distance, web service. BMC Genet 6: 13 15760479 10.1186/1471-2156-6-13 PMC1079815

[83] McRae B, Beier P, Huynh L, DeWald L, Keim P (2005) Habitat barriers limit gene flow and illuminate historical events in a wide ranging carnivore, the American puma. Mol Ecol 14: 1965–1977. 15910319 10.1111/j.1365-294x.2005.02571.x

[84] Fontaine MC, Baird SJE, Piry S, Ray N, Tolley KA, Duke S, et al (2007) Rise of oceanographic barriers in continuous populations of a cetacean: the genetic structure of harbor porpoises in old world waters. BMC Biology 5: 30 17651495 10.1186/1741-7007-5-30 PMC1971045

[85] Rosenberg MS, Anderson CD (2011) PASSaGE: Pattern Analysis, Spatial Statistics and Geographic Exegesis. Version 2. Method Ecol Evol 2: 229–232.

[86] Castella V, Ruedi M, Excoffier L (2001) Contrasted patterns of mitochondrial and nuclear structure among nursery colonies of the bat *Myotis myotis* . J Evolution Biol 14: 708–720.

[87] Kerth G, Mayer F, Petit E (2002) Extreme sex-biased dispersal in the communally breeding, nonmigratory Bechstein's bat (*Myotis bechsteinii*). Mol Ecol 11: 1491–1498. 12144668 10.1046/j.1365-294x.2002.01528.x

[88] Moussy C, Hosken DJ, Mathews F, Smith GC, Aegerter JN, Bearhop S (2013) Migration and dispersal patterns of bats and their influence on genetic structure. Mammal Rev 43: 183–195.

[89] Bogdanowicz W, Lesiński G, Sadkowska-Todys M, Gajewska M, Rutkowski R (2013) Population genetics and bat rabies: a case study of *Eptesicus serotinus* in Poland. Acta Chiropt 15: 35–56.

[90] Shafer ABA, Cullingham CI, Cote SD, Coltman DW (2010) Of glaciers and refugia: a decade of study sheds new light on the phylogeography of northwestern North America. Mol Ecol 19: 4589–4621. 10.1111/j.1365-294X.2010.04828.x 20849561

