# Supplementary material for: Range-Wide Genetic Analysis of Little Brown Bat (Myotis lucifugus) Populations: Estimating the Risk of Spread of White-Nose Syndrome
Source: PLoS One. 2015 Jul 8;10(7):e0128713. doi: 10.1371/journal.pone.0128713 (PMC4495924; doi:10.1371/journal.pone.0128713)
Supplement: S1 Fig — (DOCX) [file pone.0128713.s001.docx]

**Figure S1. Standardized genetic distance [*F*_ST_/(1 – *F*_ST_)] for microsatellites plotted against the logarithm of geographic distance for the western population cluster with the Alaska population removed.**
