# Supplementary material for: Range-Wide Genetic Analysis of Little Brown Bat (Myotis lucifugus) Populations: Estimating the Risk of Spread of White-Nose Syndrome
Source: PLoS One. 2015 Jul 8;10(7):e0128713. doi: 10.1371/journal.pone.0128713 (PMC4495924; doi:10.1371/journal.pone.0128713)
Supplement: S1 File — (PDF) [file pone.0128713.s002.pdf]

Sources for spatial data used to produce the map (Figure 1) include:

nationalatlas.gov (available at: ), which is publicly available and has no license restrictions

iucnredlist.org (available at: ), which is publicly available and has no license restrictions (see attached documentation from their website)

ESRI Data and Maps 2006 (available at: http://downloads2.esri.com/support/whitepapers/ao_/J9509_ESRI_DataandMaps2006.pdf). The specific files used were srtm_n_relief_w.jp2 file and the gtopo30_n_relief_w.jp2 file. These files fall under "Yes 1 - Redistribution rights are granted by the data vendor for hard-copy renditions or static, electronic map images (e.g. .gif, .jpeg, etc.) that are plotted, printed, or publicly displayed with proper metadata and source/copyright attribution to the respective data vendor(s)." The metadata outlining these permissions is attached.

The maps were produced by Mr. Jason Glatz of Western Michigan University. He has provided permission to publish the maps in the attached letter

    - Limitations of the Data
    - Glossary
    - Download GIS Data
- Acknowledgements
-


# Download GIS Data

The 2009 IUCN Red List of Threatened Species contains assessments for 49,000 species of which spatial data exists for about 25,000 species, including all mammals. Some species listed as Data Deficient are not mapped. These data are made freely available to the public to help inform conservation planning and other decision making processes. Detailed information on the assessment process is available on this website here.

The data are held in shapefiles, the ESRI native format and contain the known range of each species. Ranges are depicted as polygons. DBF files accompanying each polygon contain taxonomic information, and contain information on distribution status, sources and other details about the maps (see **metadata document**).

The data is available both in ESRI File Geodatabase format and the ESRI Shapefile format and is held in geographical coordinates. Please note that the files are large, and download times could be quite lengthy.

To download the mammals range data that was used for the spatial analysis, please visit the Spatial Data Download page in the Technical Documents section of this website.

**Download mammals data here**.

ISSN 2307-8235


© International Union for Conservation of Nature and Natural Resources.

Western Michigan University

University Libraries

February 2, 2015

To Whom It May Concern:

I grant permission for the open-access journal PLOS ONE to publish "Figure 1. Map of Canada and the United States showing the distribution of described Myotis lucifugus subspecies (modified from [17]) and sampling locations" under the Creative Commons Attribution License (CCAL) CC BY 3.0 (http://creativecommons.org/licenses/by/3.0/us/). I am aware that this license allows unrestricted use and distribution, even commercially, by third parties.

Sincerely,

Jason Glatz
Maps Coordinator
Waldo Library
Western Michigan University
Kalamazoo, MI 49008

# Redistribution Rights

| Description | Data Source | File Name | Directory | Redistribution (see FAQ below) |
|---|---|---|---|---|

| **World** | | | | |
|---|---|---|---|---|
| Countries (generalized) | ArcWorld Supplement | country.* | \world | Yes 1,2,3 |
| Countries 2006 | ArcWorld Supplement | cntry06.* | \world | Yes 1,2,3 |
| Country Boundaries (generalized) | ArcWorld | country_ln.* | \world | Yes 1,2,3 |
| Country Boundaries | ArcWorld | cntry06_ln.* | \world | Yes 1,2,3 |
| Administrative Units | ArcWorld Supplement | admin.* | \world | Yes 1,2,3 |
| Administrative Boundaries | ArcWorld Supplement | admin_ln.* | \world | Yes 1,2,3 |
| Continents | ArcWorld Supplement | continent.* | \world | Yes 1,2,3 |
| Regions | ArcWorld Supplement | region.* | \world | Yes 1,2,3 |
| CountryWatch Demographics (table) | CountryWatch, US and International sources | cntrywch00.* | \world | Yes 1 |
| Country Memberships of Political Organizations (table) | CIA Factbook | pol_org.* | \world | Yes 4 |
| Cities | ArcWorld | cities.* | \world | Yes 1,2,3 |
| Gazetteer | DCW | gaz.* | \world | Yes 1,2,3 |
| Drainage Systems, Lakes, and Rivers | ArcWorld | lakes.*, rivers.*, drainage.* | \world | Yes 1,2,3 |
| World Wildlife Fund Terrestrial Ecoregions | World Wildlife Fund, DCW | wwf_terr.* | \world | No |
| World Wildlife | | | | |

| | | | | |
|---|---|---|---|---|
| Fund Marine Ecoregions | World Wildlife Fund, DCW | wwf_mar.* | \world | No |
| UTM Zones | ArcWorld Supplement | utmzone.* | \world | Yes 1,2,3 |
| Time Zones | ESRI | timezone.* | \world | Yes 1,2,3 |
| Latitude and Longitude Grids | ESRI | latlong.* | \world | Yes 1,2,3 |
| Named Latitudes and Longitudes | ESRI | geogrid.* | \world | Yes 1,2,3 |
| World Map Background | ESRI | world30.* | \world | Yes 1,2,3 |
| **Canada** | | | | |
| Provinces | DMTI Spatial Inc. | province.* | \canada | Yes 1,2 |
| Major Cities | DMTI Spatial Inc. | mjcities.* | \canada | Yes 1,2 |
| Rural Cities | DMTI Spatial Inc. | rurcities.* | \canada | Yes 1,2 |
| Urban Cities | DMTI Spatial Inc. | urbcities.* | \canada | Yes 1,2 |
| Municipalities | DMTI Spatial Inc. | municplt.* | \canada | Yes 1,2 |
| Regional Municipalities | DMTI Spatial Inc. | regnmuni.* | \canada | Yes 1,2 |
| Indian Reserves | DMTI Spatial Inc. | indnrsrv.* | \canada | Yes 1,2 |
| Highways | DMTI Spatial Inc. | mjrroads.* | \canada | Yes 1,2 |
| Railways | DMTI Spatial Inc. | rails.* | \canada | Yes 1,2 |
| FSA Postal Centroids | DMTI Spatial Inc. | postal.* | \canada | Yes 1,2 |
| Telephone Area Code Boundaries | DMTI Spatial Inc. | areacode.* | \canada | Yes 1,2 |
| National Parks | DMTI Spatial Inc. | natlpark.* | \canada | Yes 1,2 |
| Provincial Parks | DMTI Spatial Inc. | provpark.* | \canada | Yes 1,2 |
| Water Bodies | DMTI Spatial Inc. | water.* | \canada | Yes 1,2 |
| **Mexico** | | | | |
| States | Sistemas de Informacion Geografica, S.A. de C.V. | states.* | \mexico | Yes 1 |
| Cities | Sistemas de Informacion Geografica, S.A. de C.V. | city.* | \mexico | Yes 1 |

| | | | | |
|---|---|---|---|---|
| Municipalities | Sistemas de Informacion Geografica, S.A. de C.V. | admin.* | \mexico | Yes 1 |
| Urban Areas | Sistemas de Informacion Geografica, S.A. de C.V. | urban.* | \mexico | Yes 1 |
| Contours | Sistemas de Informacion Geografica, S.A. de C.V. | contours.* | \mexico | Yes 1 |
| Roads | Sistemas de Informacion Geografica, S.A. de C.V. | roads.* | \mexico | Yes 1 |
| Railroads | Sistemas de Informacion Geografica, S.A. de C.V. | rails.* | \mexico | Yes 1 |
| Rivers and Streams | Sistemas de Informacion Geografica, S.A. de C.V. | hydro_l.* | \mexico | Yes 1 |
| Water Bodies | Sistemas de Informacion Geografica, S.A. de C.V. | hydro_p.* | \mexico | Yes 1 |

| **United States** | | | | |
|---|---|---|---|---|
| States and Counties (generalized) | ArcUSA, US Census, ESRI (Pop2005 field) | states.*, counties.* | \usa\census | US Census - Yes 1,2,3; ESRI - Yes 1,2,3 |
| States and Counties | ESRI, derived from Tele Atlas, US Census, ESRI (Pop2005 field) | dtl_st.*, dtl_cnty.* | \usa\census | Tele Atlas - Yes 1,2; US Census - Yes 1,2,3; ESRI - Yes 1,2,3 |
| State and County Boundaries | ESRI, derived from Tele Atlas | dtl_st_ln.*, dtl_cty_ln.* | \usa\census | Tele Atlas - Yes 1,2 |

| | | | | |
|---|---|---|---|---|
| County Population Estimates (tables) | US Census, Federal State Cooperative Program for Population Estimates | popestmt90.*, popestmt00.* | \usa\census | Yes 4 |
| Census Tracts | Tele Atlas, US Census, ESRI (Pop2005 field) | tracts.* | \usa\census | Tele Atlas - Yes 1,2; US Census - Yes 1,2,3; ESRI - Yes 1,2,3 |
| Census Feature Class Codes (table) | US Census | cfcc.dbf | \usa\census | Yes 4 |
| 108th Congressional Districts | ESRI | cd108.* | \usa\census | Yes 1,2,3 |
| 109th Congressional Districts | ESRI | cd109.* | \usa\census | Yes 1,2,3 |
| Cities | US Census | cities.* | \usa\census | Yes 4 |
| Populated Place Points | US Census | places.* | \usa\census | Yes 4 |
| Populated Place Areas | Tele Atlas, US Census | placeply.* | \usa\census | Yes 1,2 |
| Core Based Statistical Areas | Tele Atlas | cbsa.* | \usa\census | Yes 1,2 |
| Major Roads | Tele Atlas | mjrrds.* | \usa\trans | Yes 1,2 |
| Highways | ESRI | highways.* | \usa\trans | Yes 1,2,3 |
| Major Highways | ESRI | mjr_hwys.* | \usa\trans | Yes 1,2,3 |
| National Transportation Atlas - Interstate Highways | US Bureau Transportation Statistics | intrstat.* | \usa\trans | Yes 4 |
| National Transportation Atlas - Railroads | US Bureau Transportation Statistics | rail100k.* | \usa\trans | Yes 4 |
| Census Urbanized Areas | US Census | urban.* | \usa\census | Yes 4 |
| Major Parks | National Park | parks.* | \usa\landmarks | Yes 1,2 |

| | | | | |
|---|---|---|---|---|
| | Service, ArcUSA, Tele Atlas | | | |
| Drainage Systems, Lakes, and Rivers (generalized) | ArcWorld | drainage.*, lakes.*, and rivers.* | \usa\hydro | Yes 1,2, |
| Telephone Area Code Boundaries | Tele Atlas | areacode.* | \usa\census | Yes 1,2 |
| ZIP Code Points | Tele Atlas | zip_usa.* | \usa\census | Yes 1,2 |
| ZIP Code Areas (Five-Digit) | Tele Atlas, ESRI (Pop2005 field) | zip_poly.* | \usa\census | Tele Atlas - Yes 1,2; ESRI - Yes 1,2,3 |
| ZIP Code Areas (Three-Digit) | ESRI, derived from Tele Atlas, ESRI (Pop2005 field) | zip3.* | \usa\census | Tele Atlas - Yes 1,2; ESRI - Yes 1,2,3 |
| National Atlas - Airports | National Atlas of the United States | airports.* | \usa\trans | Yes 4 |
| National Atlas - Cities | National Atlas of the United States | cities_dtl.* | \usa\census | Yes 4 |
| National Atlas - Urbanized Areas | National Atlas of the United States | urban_dtl.* | \usa\census | Yes 4 |
| National Atlas - Federal and Indian Land Areas | National Atlas of the United States | fedlandp.* | \usa\other | Yes 4 |
| National Atlas - Federal Land Lines | National Atlas of the United States, USGS | fedlandl.* | \usa\other | Yes 4 |
| National Atlas - Water Feature Areas and Lines | National Atlas of the United States, USGS | hydroply.*, hydroln.* | \usa\hydro | Yes 4 |
| National Atlas - Public Land Survey | National Atlas of the United States, USGS | publdsur.* | \usa\other | Yes 4 |
| National Atlas - Historic Earthquakes | National Atlas of the United States, USGS | quakehis.* | \usa\landmarks | Yes 4 |
| National Atlas - | Smithsonian | volcano.* | \usa\landmarks | Yes 4 |

| | | | | |
|---|---|---|---|---|
| Volcanoes | Institution, Global Volcanism Program, National Atlas of the United States | | | |
| Airports | Tele Atlas | airportp.* | \usa\trans | Yes 1,2 |
| Institutions | Tele Atlas | institut.* | \usa\landmarks | Yes 1,2 |
| Large Area Landmarks | Tele Atlas | lalndmrk.* | \usa\landmarks | Yes 1,2 |
| Parks | Tele Atlas | park_dtl.* | \usa\landmarks | Yes 1,2 |
| Recreation Areas | Tele Atlas | recareas.* | \usa\landmarks | Yes 1,2 |
| Transportation Terminals | Tele Atlas | tranterm.* | \usa\trans | Yes 1,2 |
| Hospitals | American Hospital Association | hospitals.* | \usa\landmarks | No |
| Geographic Names Information System - Buildings | USGS - GNIS | gblding.* | \usa\landmarks | Yes 4 |
| Geographic Names Information System - Cemeteries | USGS - GNIS | gcemetry.* | \usa\landmarks | Yes 4 |
| Geographic Names Information System - Churches | USGS - GNIS | gchurch.* | \usa\landmarks | Yes 4 |
| Geographic Names Information System - Golf Locales | USGS - GNIS | ggolf.* | \usa\landmarks | Yes 4 |
| Geographic Names Information System - Hospitals | USGS - GNIS | ghospitl.* | \usa\landmarks | Yes 4 |

| | | | | |
|---|---|---|---|---|
| Geographic Names Information System - Locales | USGS - GNIS | glocale.* | \usa\landmarks | Yes 4 |
| Geographic Names Information System - Populated Places | USGS - GNIS | gppl.* | \usa\landmarks | Yes 4 |
| Geographic Names Information System - Schools | USGS - GNIS | gschools.* | \usa\landmarks | Yes 4 |
| Geographic Names Information System - Summits | USGS - GNIS | gsummit.* | \usa\landmarks | Yes 4 |
| State Plane Zones (NAD 1927, NAD 1983) | NOAA, USGS, ESRI | spcszn27.*, spcszn83.* | \usa\other | Yes 1,2,3 |
| USGS Topographic Quadrangle Series Indexes - 1:24,000, 1:100,000, 1:250,000 | ArcUSA | topoq24.*, topoq100.*, topoq250.* | \usa\other | Yes 1,2,3 |
| Census Block Groups | Tele Atlas, US Census, ESRI (Pop2005) | blkgrp.* | \usa\census | Tele Atlas - Yes 1,2; US Census - Yes 1,2,3; ESRI - Yes 1,2,3 |
| Census Block Centroid Populations | US Census | blockpop.* | \usa\census | Yes 4 |
| Rivers and Streams | USGS, ESRI | dtl_riv.* | \usa\hydro | Yes 1,2,3 |
| Water Bodies | USGS, ESRI | dtl_wat.* | \usa\hydro | Yes 1,2,3 |

| | | | | |
|---|---|---|---|---|
| StreetMap USA Detailed Streets | ESRI | streets.* | \Usa\Streetmap_usa\Streets | Yes 1,2,3 |
| **Europe** | | | | |
| Europe Base Map - Countries | AND Data Solutions, B.V. | country.* | \europe\basemap | No |
| Europe Base Map - Level 1 Provinces | AND Data Solutions, B.V. | prov1.* | \europe\basemap | No |
| Europe Base Map - Level 2 Provinces | AND Data Solutions, B.V. | prov2.* | \europe\basemap | No |
| Europe Base Map - Level 3 Provinces | AND Data Solutions, B.V. | prov3.* | \europe\basemap | No |
| Europe Base Map - Cities | AND Data Solutions, B.V. | cities.* | \europe\basemap | No |
| Europe Base Map - Places | AND Data Solutions, B.V. | places.* | \europe\basemap | No |
| Europe Base Map - Urbanized Areas | AND Data Solutions, B.V. | urban.* | \europe\basemap | No |
| Europe Base Map - Major Roads | AND Data Solutions, B.V. | mjrroads.* | \europe\basemap | No |
| Europe Base Map - Roads | AND Data Solutions, B.V. | roads.* | \europe\basemap | No |
| Europe Base Map - Railroads | AND Data Solutions, B.V. | rails.* | \europe\basemap | No |
| Europe Base Map - Major Lakes | AND Data Solutions, B.V. | mjrlakes.* | \europe\basemap | No |
| Europe Base Map - Major Rivers | AND Data Solutions, B.V. | mjrrivers.* | \europe\basemap | No |
| Europe Base Map - Water Bodies | AND Data Solutions, B.V. | water.* | \europe\basemap | No |
| Europe Base Map - Ferries | AND Data Solutions, B.V. | ferries.* | \europe\basemap | No |
| Europe Base Map - Railroad | AND Data Solutions, B.V. | rr_stns.* | \europe\basemap | No |

| | | | | |
|---|---|---|---|---|
| Stations | | | | |
| Europe Demographic - NUTS 0 Demographics | Michael Bauer Research GmbH I.G.; EuroGeographics; AND Data Solutions, B.V. | nuts0.* | \europe\demography | Michael Bauer - Yes 1; EuroGeographics - Yes 1; AND Data Solutions - No |
| Europe Demographic - NUTS 1 Demographics | Michael Bauer Research GmbH I.G.; EuroGeographics; AND Data Solutions, B.V. | nuts1.* | \europe\demography | Michael Bauer - Yes 1; EuroGeographics - Yes 1; AND Data Solutions - No |
| Europe Demographic - NUTS 2 Demographics | Michael Bauer Research GmbH I.G.; EuroGeographics; AND Data Solutions, B.V. | nuts2.* | \europe\demography | Michael Bauer - Yes 1; EuroGeographics - Yes 1; AND Data Solutions - No |
| Europe Demographic - NUTS 3 Demographics | Michael Bauer Research GmbH I.G.; EuroGeographics; AND Data Solutions, B.V. | nuts3.* | \europe\demography | Michael Bauer - Yes 1; EuroGeographics - Yes 1; AND Data Solutions - No |

| | | | | |
|---|---|---|---|---|
| **Image Data** | | | | |
| Global Digital Elevation Model (SRTM) | SRTM: NASA, NGA, USGS EROS, ESRI<br><br>GTOPO30: USGS EROS, ESRI | srtm_n_elev_c.jp2, srtm_n_elev_e.jp2, srtm_n_elev_w.jp2, srtm_s_elev_c.jp2, srtm_s_elev_e.jp2, srtm_s_elev_w.jp2, gtopo30_n_elev_c.jp2, gtopo30_n_elev_e.jp2, gtopo30_n_elev_w.jp2, gtopo30_s_elev_c.jp2, gtopo30_s_elev_e.jp2, gtopo30_s_elev_w.jp2 | \srtm_void_filled\elevation | Yes 1,2 |
| Global Digital Elevation Model (ETOPO2) | National Geophysical Data Center | etopo2 | \world_elevation | Yes 1,2,3 |
| Global Digital Elevation Model | USGS EROS Data Center | gtopo30 | \world_elevation | Yes 1,2,3 |

| | | | | |
|---|---|---|---|---|
| (GTOPO30) | | | | |
| Global Shaded Relief | SRTM: NASA, NGA, USGS EROS, ESRI<br><br>GTOPO30: USGS EROS, ESRI | gtopo30_n_relief_w.jp2, gtopo30_s_relief_w.jp2, srtm_n_relief_w.jp2, srtm_s_relief_w.jp2, gtopo30_n_relief_c.jp2, gtopo30_s_relief_c.jp2, srtm_n_relief_c.jp2, srtm_s_relief_c.jp2, gtopo30_n_relief_e.jp2, gtopo30_s_relief_e.jp2, srtm_n_relief_e.jp2, srtm_s_relief_e.jp2 | \shaded_relief | Yes 1,2 |
| Global Imagery 150-meter Resolution | Earth Satellite Corporation | africa_150m_earthsat.jp2, asia_east_150m_earthsat.jp2, asia_west_150m_earthsat.jp2, australia_150m_earthsat.jp2, europe_150m_earthsat.jp2, northamerica_north_150m_earthsat.jp2, northamerica_south_150m_earthsat.jp2, southamerica_150m_earthsat.jp2 | \global_imagery | Yes 2 |
| World Topography and Bathymetry | DCW, NOAA, UCSD | world.sid | \world_elevation | No |
| WorldSat Color Shaded Relief Image | WorldSat International, Inc. | WSI-Earth99-2k.sid | \world_images | No |
| World Cloud Free Image | NASA | earth_1km.jp2 | \world_images | Yes 1,2,3 |
| World with Clouds Image | NASA | earth_clouds_5km.bil | \world_images | Yes 1,2,3 |
| World with Ice Image | NASA | earth_ice_5km.bil | \world_images | Yes 1,2,3 |
| World at Night Image | NASA | earth_city_lights_5km.bil | \world_images | Yes 1,2,3 |
| Global Elevation Index | ESRI | elev_index.* | \srtm_void_filled\data_quality | Yes 1,2 |
| GTOPO30 | | | | |

| | | | | |
|---|---|---|---|---|
| Source Index | USGS EROS, ESRI | gtopo30_source.* | \srtm_void_filled\data_quality | Yes 1,2 |
| Void Areas (SRTM) | NASA, NGA, ESRI | srtm_n_nodata_c.sdc, srtm_n_nodata_e.sdc, srtm_n_nodata_w.sdc, srtm_s_nodata_c.sdc, srtm_s_nodata_e.sdc, srtm_s_nodata_w.sdc | \srtm_void_filled\data_quality | Yes 1,2 |
| Water Bodies | SRTM: NASA, NGA, ESRI<br><br>GTOPO30: DCW, USGS EROS, ESRI | gtopo30_n_water_c.sdc, gtopo30_n_water_e.sdc, gtopo30_n_water_w.sdc, gtopo30_s_water_c.sdc, gtopo30_s_water_e.sdc, gtopo30_s_water_w.sdc, srtm_n_water_c.sdc, srtm_n_water_e.sdc, srtm_n_water_w.sdc, srtm_s_water_c.sdc, srtm_s_water_e.sdc, srtm_s_water_w.sdc | \srtm_void_filled\water | Yes 1,2 |

All data are available for internal use.

# Frequently Asked Questions (FAQ)

Q: Are all of the sample data provided on the ESRI Data & Maps disks freely redistributable?

A: No. Much of the sample data are provided by multiple, third party data vendors under license to ESRI for inclusion on these disks specifically for use with ESRI software.

Q: What does the last column "Redistribution" on the Redistribution Rights matrix with its "Yes" or "No" answers mean?

A: Each data vendor has its own data licensing policies and may grant varying redistribution rights to end users. Please consult the Redistribution Rights Codes below to determine the redistribution rights for a certain sample data file provided on the CD–ROMs and DVD-ROMs. As used herein, "Geodata" shall mean any digital data set consisting of geographic data coordinates and associated attributes.

"No" Internal Use Only. No redistribution rights are granted by the data vendor and the data is for the end user's own internal use only.

"Yes 1" Redistribution rights are granted by the data vendor for hard-copy renditions or static, electronic map images (e.g. .gif, .jpeg, etc.) that are plotted, printed, or publicly displayed with proper metadata and source/copyright attribution to the respective data vendor(s).

"Yes 2" Geodata are redistributable with a Value-Added Software Application developed by ESRI Business

Partners on a royalty-free basis with proper metadata and source/copyright attribution to the respective data vendor(s).

“Yes 3" Geodata are redistributable without a Value-Added Software Application (i.e., adding the sample data to an existing, [non]commercial data set for redistribution) with proper metadata and source/copyright attribution to the respective data vendor(s).

“Yes 4" Public domain data from US government are freely redistributable with proper metadata and source attribution.

Q: Are there any legal terms and conditions I need to be aware of under this license to use the sample data provided on the ESRI Data & Maps disks?

A: Yes. The terms and conditions below apply to all the sample data sets provided on the disks.

**High Risk Activities:** (a) The Software, Data, and Documentation are not fault-tolerant and are not designed, manufactured, or intended for use or resale for insurance underwriting or with critical health and safety or online control equipment in hazardous environments that require fail-safe performance, such as in the operation of nuclear facilities, aircraft navigation, or communication systems, air traffic control, emergency response, terrorism prevention or response, life support, or weapons systems ("High Risk Activities"). ESRI SPECIFICALLY DISCLAIMS ANY EXPRESS OR IMPLIED WARRANTY OF FITNESS FOR HIGH RISK ACTIVITIES.

(b) To the extent permitted by law, Licensee agrees to indemnify, defend, and hold ESRI, its officers, directors, employees, agents, subcontractors, licensors, successors, and assigns harmless from and against any and all liability, losses, claims, expenses (including attorneys' fees), demands, or damages of any kind, including direct, indirect, special, punitive, incidental, or consequential damages, arising out of or in any way connected with Licensee's use or permitting the use by others of the Software, Data, and vendor's hardware for High Risk Activities. Delivery of the Software, Data, and vendor's hardware does not constitute a waiver of the rights and obligations set forth in this Article.

**Proprietary Rights and Copyright:** Licensee acknowledges that the Data and Related Materials contain proprietary and confidential property of ESRI and its licensor(s). The Data and Related Materials are owned by ESRI and its licensor(s) and are protected by United States copyright laws and applicable international copyright treaties and/or conventions.

**Limited Warranty and Disclaimer:** ESRI warrants that the media upon which the Data and Related Materials are provided will be free from defects in materials and workmanship under normal use and service for a period of ninety (90) days from the date of receipt.

**THE DATA AND RELATED MATERIALS ARE EXCLUDED FROM THE LIMITED WARRANTY, AND THE LICENSEE EXPRESSLY ACKNOWLEDGES THAT THE DATA CONTAIN SOME NONCONFORMITIES, DEFECTS, OR ERRORS. ESRI DOES NOT WARRANT THAT THE DATA WILL MEET LICENSEE'S NEEDS OR EXPECTATIONS; THAT THE USE OF THE DATA WILL BE UNINTERRUPTED; OR THAT ALL NONCONFORMITIES, DEFECTS, OR ERRORS CAN OR WILL BE CORRECTED. ESRI IS NOT INVITING RELIANCE ON THESE DATA, AND THE LICENSEE SHOULD ALWAYS VERIFY ACTUAL DATA.**

**EXCEPT FOR THE LIMITED WARRANTY SET FORTH ABOVE, THE DATA AND RELATED MATERIALS CONTAINED THEREIN ARE PROVIDED "AS-IS," WITHOUT WARRANTY OF ANY KIND, EITHER EXPRESS OR IMPLIED, INCLUDING, BUT NOT LIMITED TO, THE IMPLIED WARRANTIES OF MERCHANTABILITY AND FITNESS FOR A PARTICULAR PURPOSE.**

**Exclusive Remedy and Limitation of Liability:** The entire liability of ESRI or its licensor(s) and Licensee's exclusive remedy shall be to terminate the Agreement upon Licensee returning the Data and Related Materials to ESRI with a copy of Licensee's invoice/receipt and ESRI returning the license fees paid to Licensee.

**IN NO EVENT SHALL ESRI AND/OR ITS LICENSOR(S) BE LIABLE FOR COSTS OF PROCUREMENT OF SUBSTITUTE GOODS OR SERVICES; LOST PROFITS, LOST SALES, OR BUSINESS EXPENDITURES, INVESTMENTS, OR COMMITMENTS IN CONNECTION WITH ANY BUSINESS; LOSS OF ANY GOODWILL; OR FOR ANY INDIRECT, SPECIAL, INCIDENTAL, EXEMPLARY, OR CONSEQUENTIAL DAMAGES ARISING OUT OF THIS AGREEMENT OR USE OF THE DATA AND RELATED MATERIALS, HOWEVER CAUSED, ON ANY THEORY OF LIABILITY, AND WHETHER OR NOT ESRI OR ITS LICENSOR(S) HAVE BEEN ADVISED OF THE POSSIBILITY OF SUCH DAMAGE. THESE LIMITATIONS SHALL APPLY NOTWITHSTANDING ANY FAILURE OF ESSENTIAL PURPOSE OF ANY EXCLUSIVE REMEDY.**

**Third Party Beneficiary:** ESRI's licensor(s) has (have) authorized ESRI to (sub)distribute and (sub)license its (their) data as incorporated into the Data and Related Materials. As an intended third party beneficiary to this Agreement, the ESRI licensor(s) is (are) entitled to directly enforce, in its own name, the rights and obligations undertaken by the Licensee and to seek all legal and equitable remedies as are afforded to ESRI.

Q: How should I attribute the sample data provided on the CD–ROMs and DVD-ROMs if I use it as proscribed above?

A: In the event that the data vendor(s) has (have) granted the end user permission to redistribute the Geodata, please use proper proprietary or copyright attribution for the various data vendor(s), and provide the associated metadata file(s) with the Geodata. In compliance with FGDC metadata standards, ESRI has attempted to practice proper metadata methodologies by providing any data source information, descriptions, and file names to assist in this effort.
