# Supplementary material for: Range-Wide Genetic Analysis of Little Brown Bat (Myotis lucifugus) Populations: Estimating the Risk of Spread of White-Nose Syndrome
Source: PLoS One. 2015 Jul 8;10(7):e0128713. doi: 10.1371/journal.pone.0128713 (PMC4495924; doi:10.1371/journal.pone.0128713)
Supplement: S1 Table — (DOCX) [file pone.0128713.s003.docx]

Table S1. List of *Myotis lucifugus* specimens included in analyses. Population refers to those listed in Table 1 and Figure 1. Haplotypes were determined by collapsing the mtDNA COI dataset to unique haplotypes; individuals sharing the same number share the same haplotype. Genbank accession numbers are provided for each unique haplotype the first time they appear in the table. Sex (m= male, f=female) and age (a=adult, j=juvenile) are provided for each specimen.

| Sample ID | Population | Genbank | Haplotype | Collection Date | Sex | Age | Province or State | County | Country | Location | Source |
| --- | --- | --- | --- | --- | --- | --- | --- | --- | --- | --- | --- |
| SL-01 | AK | KM363877 | 1 | 14-Jul-2011 | m | a | Alaska | Kenai Peninsula | USA | Moose Creek | Susan Loeb |
| SL-02 | AK |  | 1 | 14-Jul-2011 | m | a | Alaska | Kenai Peninsula | USA | Moose Creek | Susan Loeb |
| SL-03 | AK |  | 1 | 21-Jul-2011 | f | a | Alaska | Kenai Peninsula | USA | Glaser House | Susan Loeb |
| SL-04 | AK |  | 1 | 21-Jul-2011 | f | a | Alaska | Kenai Peninsula | USA | Glaser House | Susan Loeb |
| SL-05 | AK | KM363878 | 2 | 21-Jul-2011 | f | a | Alaska | Kenai Peninsula | USA | Glaser House | Susan Loeb |
| SL-06 | AK |  | 1 | 21-Jul-2011 | m | j | Alaska | Kenai Peninsula | USA | Glaser House | Susan Loeb |
| SL-07 | AK | KM363879 | 3 | 21-Jul-2011 | f | a | Alaska | Kenai Peninsula | USA | Glaser House | Susan Loeb |
| SL-08 | AK |  | 1 | 21-Jul-2011 | f | a | Alaska | Kenai Peninsula | USA | Glaser House | Susan Loeb |
| SL-09 | AK |  | 2 | 21-Jul-2011 | f | a | Alaska | Kenai Peninsula | USA | Glaser House | Susan Loeb |
| SL-10 | AK |  | 1 | 21-Jul-2011 | f | a | Alaska | Kenai Peninsula | USA | Glaser House | Susan Loeb |
| SL-11 | AK |  | 3 | 21-Jul-2011 | f | a | Alaska | Kenai Peninsula | USA | Glaser House | Susan Loeb |
| SL-12 | AK |  | 3 | 21-Jul-2011 | f | a | Alaska | Kenai Peninsula | USA | Glaser House | Susan Loeb |
| SL-13 | AK |  | 2 | 21-Jul-2011 | f | a | Alaska | Kenai Peninsula | USA | Glaser House | Susan Loeb |
| SL-14 | AK |  | 1 | 21-Jul-2011 | f | a | Alaska | Kenai Peninsula | USA | Glaser House | Susan Loeb |
| SL-15 | AK | KM363880 | 4 | 21-Jul-2011 | f | a | Alaska | Kenai Peninsula | USA | Glaser House | Susan Loeb |
| SL-16 | AK |  | 1 | 21-Jul-2011 | f | a | Alaska | Kenai Peninsula | USA | Glaser House | Susan Loeb |
| SL-17 | AK |  | 3 | 21-Jul-2011 | f | a | Alaska | Kenai Peninsula | USA | Glaser House | Susan Loeb |
| SL-18 | AK |  | 1 | 21-Jul-2011 | f | a | Alaska | Kenai Peninsula | USA | Glaser House | Susan Loeb |
| DP-34 | CA-Si | KM363881 | 5 | 26-Jun-1999 | f | a | California | Siskiyou | USA | Meiss Ranch | Dixie Pierson |
| DP-35 | CA-Si | KM363882 | 6 | 26-Jun-1999 | f | a | California | Siskiyou | USA | Meiss Ranch | Dixie Pierson |
| DP-36 | CA-Si |  | 5 | 26-Jun-1999 | f | a | California | Siskiyou | USA | Meiss Ranch | Dixie Pierson |
| DP-37 | CA-Si | KM363883 | 7 | 26-Jun-1999 | f | a | California | Siskiyou | USA | Meiss Ranch | Dixie Pierson |
| DP-38 | CA-Si | KM363884 | 8 | 26-Jun-1999 | f | a | California | Siskiyou | USA | Meiss Ranch | Dixie Pierson |
| DP-39 | CA-Si |  | 6 | 26-Jun-1999 | f | a | California | Siskiyou | USA | Meiss Ranch | Dixie Pierson |
| DP-40 | CA-Si |  | 7 | 26-Jun-1999 | f | a | California | Siskiyou | USA | Meiss Ranch | Dixie Pierson |
| DP-41 | CA-Si | KM363885 | 9 | 26-Jun-1999 | f | a | California | Siskiyou | USA | Meiss Ranch | Dixie Pierson |
| DP-42 | CA-Si | KM363886 | 10 | 26-Jun-1999 | f | a | California | Siskiyou | USA | Meiss Ranch | Dixie Pierson |
| DP-43 | CA-Si |  | 6 | 26-Jun-1999 | f | a | California | Siskiyou | USA | Meiss Ranch | Dixie Pierson |
| DP-44 | CA-Si |  | 6 | 26-Jun-1999 | f | a | California | Siskiyou | USA | Meiss Ranch | Dixie Pierson |
| DP-45 | CA-Si |  | 8 | 26-Jun-1999 | f | a | California | Siskiyou | USA | Meiss Ranch | Dixie Pierson |
| DP-46 | CA-Si |  | 5 | 26-Jun-1999 | f | a | California | Siskiyou | USA | Meiss Ranch | Dixie Pierson |
| DP-47 | CA-Si |  | 6 | 26-Jun-1999 | f | a | California | Siskiyou | USA | Meiss Ranch | Dixie Pierson |
| DP-48 | CA-Si |  | 10 | 26-Jun-1999 | f | a | California | Siskiyou | USA | Meiss Ranch | Dixie Pierson |
| DP-50 | CA-Si | KM363887 | 11 | 26-Jun-1999 | f | a | California | Siskiyou | USA | Meiss Ranch | Dixie Pierson |
| DP-51 | CA-Si |  | 6 | 26-Jun-1999 | f | a | California | Siskiyou | USA | Meiss Ranch | Dixie Pierson |
| DP-52 | CA-Si |  | 7 | 26-Jun-1999 | f | a | California | Siskiyou | USA | Meiss Ranch | Dixie Pierson |
| DP-53 | CA-Si |  | 6 | 26-Jun-1999 | f | a | California | Siskiyou | USA | Meiss Ranch | Dixie Pierson |
| DP-54 | CA-Si |  | 10 | 26-Jun-1999 | f | a | California | Siskiyou | USA | Meiss Ranch | Dixie Pierson |
| DP-24 | CA-Ma | KM363888 | 12 | 4-Aug-1998 | m | a | California | Mariposa | USA | Tenaya Creek | Dixie Pierson |
| DP-25 | CA-Ma |  | 12 | 4-Aug-1998 | f | a | California | Mariposa | USA | Tenaya Creek | Dixie Pierson |
| DP-26 | CA-Ma |  | 12 | 4-Aug-1998 | f | a | California | Mariposa | USA | Tenaya Creek | Dixie Pierson |
| DP-27 | CA-Ma |  | 12 | 4-Aug-1998 | f | a | California | Mariposa | USA | Tenaya Creek | Dixie Pierson |
| DP-28 | CA-Ma |  | 12 | 4-Aug-1998 | f | a | California | Mariposa | USA | Tenaya Creek | Dixie Pierson |
| DP-29 | CA-Ma |  | 12 | 4-Aug-1998 | f | a | California | Mariposa | USA | Tenaya Creek | Dixie Pierson |
| DP-30 | CA-Ma |  | 12 | 4-Aug-1998 | f | a | California | Mariposa | USA | Tenaya Creek | Dixie Pierson |
| DP-31 | CA-Ma |  | 12 | 4-Aug-1998 | f | a | California | Mariposa | USA | Tenaya Creek | Dixie Pierson |
| DP-32 | CA-Ma |  | 12 | 4-Aug-1998 | f | a | California | Mariposa | USA | Tenaya Creek | Dixie Pierson |
| DP-33 | CA-Ma | KM363889 | 13 | 4-Aug-1998 | f | a | California | Mariposa | USA | Tenaya Creek | Dixie Pierson |
| DP-55 | CA-Mo |  | 13 | 18-Aug-1999 | f | a | California | Mono | USA | Lee Vining Creek | Dixie Pierson |
| DP-56 | CA-Mo |  | 13 | 18-Aug-1999 | m | a | California | Mono | USA | Lee Vining Creek | Dixie Pierson |
| DP-57 | CA-Mo |  | 13 | 18-Aug-1999 | m | a | California | Mono | USA | Lee Vining Creek | Dixie Pierson |
| DP-58 | CA-Mo |  | 13 | 18-Aug-1999 | m | j | California | Mono | USA | Lee Vining Creek | Dixie Pierson |
| DP-59 | CA-Mo |  | 13 | 18-Aug-1999 | m | a | California | Mono | USA | Lee Vining Creek | Dixie Pierson |
| DP-60 | CA-Mo |  | 13 | 18-Aug-1999 | m | a | California | Mono | USA | Lee Vining Creek | Dixie Pierson |
| DP-61 | CA-Mo |  | 13 | 18-Aug-1999 | m | a | California | Mono | USA | Lee Vining Creek | Dixie Pierson |
| DP-62 | CA-Mo |  | 13 | 18-Aug-1999 | m | a | California | Mono | USA | Lee Vining Creek | Dixie Pierson |
| DP-63 | CA-Mo |  | 13 | 18-Aug-1999 | m | a | California | Mono | USA | Lee Vining Creek | Dixie Pierson |
| DP-64 | CA-Mo |  | 13 | 18-Aug-1999 | m | a | California | Mono | USA | Lee Vining Creek | Dixie Pierson |
| DP-65 | CA-Mo | KM363890 | 14 | 18-Aug-1999 | m | a | California | Mono | USA | Lee Vining Creek | Dixie Pierson |
| DP-66 | CA-Mo |  | 14 | 18-Aug-1999 | m | a | California | Mono | USA | Lee Vining Creek | Dixie Pierson |
| DP-67 | CA-Mo |  | 14 | 18-Aug-1999 | m | a | California | Mono | USA | Lee Vining Creek | Dixie Pierson |
| DP-68 | CA-Mo |  | 14 | 18-Aug-1999 | m | j | California | Mono | USA | Lee Vining Creek | Dixie Pierson |
| DP-69 | CA-Mo |  | 14 | 18-Aug-1999 | m | j | California | Mono | USA | Lee Vining Creek | Dixie Pierson |
| DP-75 | CA-Sh | KM363891 | 15 | 16-Jul-2000 | f | a | California | Shasta | USA | Pit River | Dixie Pierson |
| DP-76 | CA-Sh |  | 15 | 16-Jul-2000 | f | a | California | Shasta | USA | Pit River | Dixie Pierson |
| DP-77 | CA-Sh |  | 10 | 16-Jul-2000 | m | a | California | Shasta | USA | Pit River | Dixie Pierson |
| DP-78 | CA-Sh |  | 15 | 16-Jul-2000 | f | a | California | Shasta | USA | Pit River | Dixie Pierson |
| DP-79 | CA-Sh |  | 15 | 16-Jul-2000 | m | a | California | Shasta | USA | Pit River | Dixie Pierson |
| DP-80 | CA-Sh |  | 15 | 16-Jul-2000 | f | a | California | Shasta | USA | Pit River | Dixie Pierson |
| DP-81 | CA-Sh |  | 15 | 16-Jul-2000 | m | a | California | Shasta | USA | Pit River | Dixie Pierson |
| DP-82 | CA-Sh |  | 15 | 16-Jul-2000 | m | a | California | Shasta | USA | Pit River | Dixie Pierson |
| DP-83 | CA-Sh |  | 15 | 16-Jul-2000 | f | a | California | Shasta | USA | Pit River | Dixie Pierson |
| DP-84 | CA-Sh |  | 15 | 16-Jul-2000 | m | a | California | Shasta | USA | Pit River | Dixie Pierson |
| DP-85 | CA-Sh |  | 15 | 16-Jul-2000 | f | a | California | Shasta | USA | Pit River | Dixie Pierson |
| NC-08 | WA | KM363892 | 16 | 31-Jul-2001 | m | a | Washington | Skagit | USA | North Cascades National Park | Roger Christophersen |
| NC-09 | WA | KM363893 | 17 | 31-Jul-2001 | m | a | Washington | Skagit | USA | North Cascades National Park | Roger Christophersen |
| NC-11 | WA |  | 16 | 7-Aug-2001 | m | a | Washington | Whatcom | USA | North Cascades National Park | Roger Christophersen |
| NC-12 | WA |  | 16 | 7-Aug-2001 | m | a | Washington | Whatcom | USA | North Cascades National Park | Roger Christophersen |
| NC-13 | WA |  | 16 | 7-Aug-2001 | m | a | Washington | Whatcom | USA | North Cascades National Park | Roger Christophersen |
| NC-14 | WA |  | 16 | 8-Aug-2001 | m | a | Washington | Whatcom | USA | North Cascades National Park | Roger Christophersen |
| NC-16 | WA | KM363894 | 18 | 28-Aug-2001 | f | a | Washington | Chelan | USA | North Cascades National Park | Roger Christophersen |
| NC-17 | WA |  | 13 | 28-Aug-2001 | f | a | Washington | Chelan | USA | North Cascades National Park | Roger Christophersen |
| NC-18 | WA | KM363895 | 19 | 28-Aug-2001 | f | a | Washington | Chelan | USA | North Cascades National Park | Roger Christophersen |
| NC-20 | WA |  | 19 | 29-Aug-2001 | f | a | Washington | Chelan | USA | North Cascades National Park | Roger Christophersen |
| NC-21 | WA |  | 16 | 29-Aug-2001 | m | a | Washington | Chelan | USA | North Cascades National Park | Roger Christophersen |
| NC-22 | WA | KM363896 | 20 | 29-Aug-2001 | m | a | Washington | Chelan | USA | North Cascades National Park | Roger Christophersen |
| NC-23 | WA |  | 16 | 29-Aug-2001 | m | a | Washington | Chelan | USA | North Cascades National Park | Roger Christophersen |
| NC-26 | WA | KM363897 | 21 | 12-Aug-2002 | f | j | Washington | Whatcom | USA | North Cascades National Park | Roger Christophersen |
| NC-29 | WA | KM363898 | 22 | 12-Aug-2002 | m | a | Washington | Whatcom | USA | North Cascades National Park | Roger Christophersen |
| NC-32 | WA |  | 16 | 12-Aug-2002 | f | a | Washington | Whatcom | USA | North Cascades National Park | Roger Christophersen |
| NC-33 | WA |  | 16 | 12-Aug-2002 | m | a | Washington | Whatcom | USA | North Cascades National Park | Roger Christophersen |
| HE05 | BC-S |  | 18 | 3-Aug-1997 | m | j | British Columbia |  | Canada | Kootenay Lake, West Arm, south shore | John Gwilliam |
| HE06 | BC-S | KM363899 | 23 | 3-Aug-1997 | f | a | British Columbia |  | Canada | Kootenay Lake, West Arm, south shore | John Gwilliam |
| HE07 | BC-S |  | 23 | 3-Aug-1997 | f | a | British Columbia |  | Canada | Kootenay Lake, West Arm, south shore | John Gwilliam |
| HE09 | BC-S | KM363900 | 24 | 3-Aug-1997 | f | a | British Columbia |  | Canada | Kootenay Lake, West Arm, south shore | John Gwilliam |
| HE13 | BC-S |  | 24 | 3-Aug-1997 | f | a | British Columbia |  | Canada | Kootenay Lake, West Arm, south shore | John Gwilliam |
| HE15 | BC-S | KM363901 | 25 | 3-Aug-1997 | f | a | British Columbia |  | Canada | Kootenay Lake, West Arm, south shore | John Gwilliam |
| HE16 | BC-S |  | 24 | 3-Aug-1997 | f | a | British Columbia |  | Canada | Kootenay Lake, West Arm, south shore | John Gwilliam |
| HE17 | BC-S |  | 24 | 3-Aug-1997 | f | a | British Columbia |  | Canada | Kootenay Lake, West Arm, south shore | John Gwilliam |
| HE18 | BC-S |  | 24 | 3-Aug-1997 | f | j | British Columbia |  | Canada | Kootenay Lake, West Arm, south shore | John Gwilliam |
| HE19 | BC-S |  | 24 | 3-Aug-1997 | f | a | British Columbia |  | Canada | Kootenay Lake, West Arm, south shore | John Gwilliam |
| HE20 | BC-S |  | 24 | 3-Aug-1997 | f | a | British Columbia |  | Canada | Kootenay Lake, West Arm, south shore | John Gwilliam |
| HE25 | BC-S |  | 24 | 3-Aug-1997 | f | a | British Columbia |  | Canada | Kootenay Lake, West Arm, south shore | John Gwilliam |
| HE28 | BC-S |  | 24 | 3-Aug-1997 | f | a | British Columbia |  | Canada | Kootenay Lake, West Arm, south shore | John Gwilliam |
| HE30 | BC-S |  | 24 | 3-Aug-1997 | f | a | British Columbia |  | Canada | Kootenay Lake, West Arm, south shore | John Gwilliam |
| HE31 | BC-S | KM363902 | 26 | 3-Aug-1997 | f | j | British Columbia |  | Canada | Kootenay Lake, West Arm, south shore | John Gwilliam |
| HE32 | BC-S |  | 24 | 3-Aug-1997 | f | a | British Columbia |  | Canada | Kootenay Lake, West Arm, south shore | John Gwilliam |
| HE33 | BC-S | KM363903 | 27 | 3-Aug-1997 | f | a | British Columbia |  | Canada | Kootenay Lake, West Arm, south shore | John Gwilliam |
| HE36 | BC-S |  | 23 | 3-Aug-1997 | f | a | British Columbia |  | Canada | Kootenay Lake, West Arm, south shore | John Gwilliam |
| HE37 | BC-S |  | 23 | 3-Aug-1997 | f | a | British Columbia |  | Canada | Kootenay Lake, West Arm, south shore | John Gwilliam |
| HE38 | BC-S |  | 23 | 3-Aug-1997 | f | a | British Columbia |  | Canada | Kootenay Lake, West Arm, south shore | John Gwilliam |
| HE41 | BC-S |  | 23 | 3-Aug-1997 | f | j | British Columbia |  | Canada | Kootenay Lake, West Arm, south shore | John Gwilliam |
| HE42 | BC-S |  | 24 | 3-Aug-1997 | f | j | British Columbia |  | Canada | Kootenay Lake, West Arm, south shore | John Gwilliam |
| HE46 | BC-S |  | 23 | 3-Aug-1997 | f | j | British Columbia |  | Canada | Kootenay Lake, West Arm, south shore | John Gwilliam |
| HE49 | BC-S |  | 23 | 3-Aug-1997 | f | a | British Columbia |  | Canada | Kootenay Lake, West Arm, south shore | John Gwilliam |
| HE52 | BC-S |  | 24 | 3-Aug-1997 | f | a | British Columbia |  | Canada | Kootenay Lake, West Arm, south shore | John Gwilliam |
| HE55 | BC-S |  | 24 | 3-Aug-1997 | f | j | British Columbia |  | Canada | Kootenay Lake, West Arm, south shore | John Gwilliam |
| HE56 | BC-S |  | 23 | 3-Aug-1997 | f | a | British Columbia |  | Canada | Kootenay Lake, West Arm, south shore | John Gwilliam |
| HE57 | BC-S | KM363904 | 28 | 3-Aug-1997 | f | a | British Columbia |  | Canada | Kootenay Lake, West Arm, south shore | John Gwilliam |
| HE58 | BC-S |  | 26 | 3-Aug-1997 | f | j | British Columbia |  | Canada | Kootenay Lake, West Arm, south shore | John Gwilliam |
| HE59 | BC-S |  | 24 | 3-Aug-1997 | f | a | British Columbia |  | Canada | Kootenay Lake, West Arm, south shore | John Gwilliam |
| FG-4556 | ID |  | 13 | 8-May-2001 | f | ? | Idaho | Bonner | USA | Sandpoint | Kari Getz |
| FG-4576 | ID | KM363905 | 29 | 24-May-2001 | f | ? | Idaho | Bonneville | USA | Idaho Falls | Kari Getz |
| FG-4585 | ID |  | 29 | 30-May-2001 | f | ? | Idaho | Bingham | USA | Blackfoot | Kari Getz |
| FG-4627 | ID |  | 29 | 22-Jun-2001 | m | ? | Idaho | Power | USA | American Falls | Kari Getz |
| FG-4628 | ID |  | 29 | 22-Jun-2001 | m | ? | Idaho | Bannock | USA | Downey | Kari Getz |
| FG-4640 | ID | KM363906 | 30 | 27-Jun-2001 | f | ? | Idaho | Bonneville | USA | Idaho Falls | Kari Getz |
| FG-4643 | ID |  | 29 | 29-Jun-2001 | f | ? | Idaho | Caribou | USA | Soda Springs | Kari Getz |
| FG-4655 | ID |  | 29 | 9-Jul-2001 | f | ? | Idaho | Bannock | USA | Pocatello | Kari Getz |
| FG-4656 | ID |  | 29 | 9-Jul-2001 | f | ? | Idaho | Franklin | USA | Preston | Kari Getz |
| FG-4657 | ID |  | 29 | 9-Jul-2001 | f | ? | Idaho | Franklin | USA | Preston | Kari Getz |
| FG-4671 | ID |  | 13 | 17-Jul-2001 | m | ? | Idaho | Bonner | USA | Sandpoint | Kari Getz |
| FG-4682 | ID |  | 29 | 20-Jul-2001 | m | ? | Idaho | Bear Lake | USA | Fish Haven | Kari Getz |
| FG-4683 | ID |  | 29 | 20-Jul-2001 | m | ? | Idaho | Caribou | USA | Soda Springs | Kari Getz |
| FG-4689 | ID |  | 30 | 25-Jul-2001 | f | ? | Idaho | Fremont | USA | St. Anthony | Kari Getz |
| FG-4691 | ID |  | 29 | 25-Jul-2001 | m | ? | Idaho | Bonneville | USA | Idaho Falls | Kari Getz |
| FG-4693 | ID | KM363907 | 31 | 26-Jul-2001 | f | ? | Idaho | Caribou | USA | Soda Springs | Kari Getz |
| FG-4699 | ID |  | 29 | 31-Jul-2001 | f | ? | Idaho | Bear Lake | USA | Montpelier | Kari Getz |
| FG-4838 | ID |  | 29 | 30-Aug-2001 | m | ? | Idaho | Power | USA | American Falls | Kari Getz |
| RAB-5394 | ID |  | 29 | 23-Jul-2002 | m | a | Idaho | Bannock | USA | Pocatello | Kari Getz |
| RAB-5436 | ID |  | 29 | 2-Aug-2002 | m | a | Idaho | Bannock | USA | Pocatello | Kari Getz |
| RAB-5462 | ID | KM363908 | 32 | 9-Aug-2002 | m | a | Idaho | Bannock | USA | Pocatello | Kari Getz |
| RAB-5480 | ID |  | 13 | 16-Aug-2002 | m |  | Idaho | Kootenai | USA | Hayden | Kari Getz |
| RAB-5504 | ID |  | 29 | 22-Aug-2002 | m | a | Idaho | Bonneville | USA | Idaho Falls | Kari Getz |
| RAB-5530 | ID |  | 29 | 29-Aug-2002 | m |  | Idaho | Bannock | USA | Pocatello | Kari Getz |
| RAB-5561 | ID |  | 29 | 10-Sep-2002 | f | a | Idaho | Madison | USA | Rexburg | Kari Getz |
| RAB-5628 | ID | KM363909 | 33 | 1-Jun-2002 | f | a | Idaho | Bannock | USA | Pocatello | Kari Getz |
| FN-97-04 | BC-N | KM363910 | 34 | 27-Jun-1997 | f | a | British Columbia |  | Canada | Fort Nelson | Steve McNalley |
| FN-97-25 | BC-N |  | 34 | 23-Jul-1997 | m | a | British Columbia |  | Canada | Fort Nelson | Steve McNalley |
| FN-97-27 | BC-N | KM363911 | 35 | 28-Jul-1997 | f | a | British Columbia |  | Canada | Fort Nelson | Steve McNalley |
| FN-97-36 | BC-N | KM363912 | 36 | 1-Aug-1997 | m | a | British Columbia |  | Canada | Fort Nelson | Steve McNalley |
| FN-97-40 | BC-N |  | 34 | 8-Aug-1997 | m | a | British Columbia |  | Canada | Fort Nelson | Steve McNalley |
| FN-97-44 | BC-N | KM363913 | 37 | 8-Aug-1997 | f | j | British Columbia |  | Canada | Fort Nelson | Steve McNalley |
| FN-98-10 | BC-N |  | 34 | 2-Jul-1998 | f | a | British Columbia |  | Canada | Fort Nelson | Maarten Vonhof |
| FN-98-11 | BC-N |  | 36 | 2-Jul-1998 | f | a | British Columbia |  | Canada | Fort Nelson | Maarten Vonhof |
| FN-98-17 | BC-N | KM363914 | 38 | 7-Jul-1998 | m | a | British Columbia |  | Canada | Fort Nelson | Maarten Vonhof |
| FN-98-19 | BC-N | KM363915 | 39 | 8-Jul-1998 | m | a | British Columbia |  | Canada | Fort Nelson | Maarten Vonhof |
| FN-98-21 | BC-N | KM363916 | 40 | 8-Jul-1998 | m | a | British Columbia |  | Canada | Fort Nelson | Maarten Vonhof |
| FN-98-23 | BC-N |  | 35 | 8-Jul-1998 | m | a | British Columbia |  | Canada | Fort Nelson | Maarten Vonhof |
| FN-98-24 | BC-N |  | 34 | 8-Jul-1998 | m | a | British Columbia |  | Canada | Fort Nelson | Maarten Vonhof |
| FN-98-29 | BC-N |  | 35 | 10-Jul-1998 | m | a | British Columbia |  | Canada | Fort Nelson | Maarten Vonhof |
| FN-98-34 | BC-N |  | 34 | 18-Jul-1998 | m | a | British Columbia |  | Canada | Fort Nelson | Maarten Vonhof |
| ALS-81 | AB-N |  | 34 | 5-Aug-2000 | f | a | Alberta |  | Canada | Lesser Slave Lake | David Hobson |
| ALS-82 | AB-N |  | 35 | 5-Aug-2000 | f | a | Alberta |  | Canada | Lesser Slave Lake | David Hobson |
| ALS-83 | AB-N |  | 34 | 5-Aug-2000 | f | a | Alberta |  | Canada | Lesser Slave Lake | David Hobson |
| ALS-84 | AB-N |  | 36 | 5-Aug-2000 | m | j | Alberta |  | Canada | Lesser Slave Lake | David Hobson |
| ALS-86 | AB-N |  | 34 | 5-Aug-2000 | f | j | Alberta |  | Canada | Lesser Slave Lake | David Hobson |
| ALS-87 | AB-N | KM363917 | 41 | 5-Aug-2000 | f | j | Alberta |  | Canada | Lesser Slave Lake | David Hobson |
| ALS-88 | AB-N |  | 35 | 5-Aug-2000 | f | j | Alberta |  | Canada | Lesser Slave Lake | David Hobson |
| ALS-89 | AB-N |  | 34 | 5-Aug-2000 | f | a | Alberta |  | Canada | Lesser Slave Lake | David Hobson |
| ALS-90 | AB-N |  | 34 | 5-Aug-2000 | m | j | Alberta |  | Canada | Lesser Slave Lake | David Hobson |
| ALS-91 | AB-N |  | 34 | 6-Aug-2000 | f | a | Alberta |  | Canada | Lesser Slave Lake | David Hobson |
| ALS-94 | AB-N |  | 35 | 6-Aug-2000 | f | a | Alberta |  | Canada | Lesser Slave Lake | David Hobson |
| ALS-95 | AB-N |  | 35 | 6-Aug-2000 | f | a | Alberta |  | Canada | Lesser Slave Lake | David Hobson |
| ALS-96 | AB-N |  | 41 | 6-Aug-2000 | f | a | Alberta |  | Canada | Lesser Slave Lake | David Hobson |
| ALS-97 | AB-N |  | 41 | 6-Aug-2000 | f | a | Alberta |  | Canada | Lesser Slave Lake | David Hobson |
| ALS-99 | AB-N | KM363918 | 42 | 6-Aug-2000 | f | a | Alberta |  | Canada | Lesser Slave Lake | David Hobson |
| ALS-100 | AB-N | KM363919 | 43 | 6-Aug-2000 | f | a | Alberta |  | Canada | Lesser Slave Lake | David Hobson |
| ALS-102 | AB-N |  | 34 | 6-Aug-2000 | f | j | Alberta |  | Canada | Lesser Slave Lake | David Hobson |
| ALS-103 | AB-N |  | 34 | 6-Aug-2000 | m | a | Alberta |  | Canada | Lesser Slave Lake | David Hobson |
| ALS-104 | AB-N |  | 34 | 6-Aug-2000 | f | j | Alberta |  | Canada | Lesser Slave Lake | David Hobson |
| ALS-105 | AB-N |  | 34 | 6-Aug-2000 | f | j | Alberta |  | Canada | Lesser Slave Lake | David Hobson |
| ALS-106 | AB-N | KM363920 | 44 | 6-Aug-2000 | f | a | Alberta |  | Canada | Lesser Slave Lake | David Hobson |
| ALS-107 | AB-N | KM363921 | 45 | 6-Aug-2000 | m | a | Alberta |  | Canada | Lesser Slave Lake | David Hobson |
| ALS-108 | AB-N |  | 41 | 6-Aug-2000 | f | j | Alberta |  | Canada | Lesser Slave Lake | David Hobson |
| ALS-109 | AB-N | KM363922 | 46 | 6-Aug-2000 | f | a | Alberta |  | Canada | Lesser Slave Lake | David Hobson |
| ALS-111 | AB-N |  | 44 | 7-Aug-2000 | m | a | Alberta |  | Canada | Lesser Slave Lake | David Hobson |
| ALS-113 | AB-N |  | 34 | 7-Aug-2000 | f | a | Alberta |  | Canada | Lesser Slave Lake | David Hobson |
| ALS-114 | AB-N |  | 44 | 7-Aug-2000 | f | a | Alberta |  | Canada | Lesser Slave Lake | David Hobson |
| ALS-115 | AB-N |  | 34 | 17-Aug-2000 | f | a | Alberta |  | Canada | Lesser Slave Lake | David Hobson |
| WOS-01 | AB-S |  | 34 | 16-Aug-2001 | f | a | Alberta |  | Canada | Writing-on-Stone P.P. | Cori Lausen |
| WOS-02 | AB-S |  | 34 | 16-Aug-2001 | f | a | Alberta |  | Canada | Writing-on-Stone P.P. | Cori Lausen |
| WOS-03 | AB-S |  | 41 | 16-Aug-2001 | m | sa | Alberta |  | Canada | Writing-on-Stone P.P. | Cori Lausen |
| WOS-04 | AB-S |  | 34 | 16-Aug-2001 | m | sa | Alberta |  | Canada | Writing-on-Stone P.P. | Cori Lausen |
| WOS-05 | AB-S |  | 41 | 16-Aug-2001 | m | a | Alberta |  | Canada | Writing-on-Stone P.P. | Cori Lausen |
| WOS-06 | AB-S | KM363923 | 47 | 16-Aug-2001 | m | a | Alberta |  | Canada | Writing-on-Stone P.P. | Cori Lausen |
| WOS-07 | AB-S |  | 34 | 16-Aug-2001 | m | a | Alberta |  | Canada | Writing-on-Stone P.P. | Cori Lausen |
| WOS-08 | AB-S |  | 41 | 16-Aug-2001 | m | a | Alberta |  | Canada | Writing-on-Stone P.P. | Cori Lausen |
| WOS-09 | AB-S |  | 34 | 16-Aug-2001 | f | sa | Alberta |  | Canada | Writing-on-Stone P.P. | Cori Lausen |
| WOS-10 | AB-S |  | 34 | 16-Aug-2001 | f | a | Alberta |  | Canada | Writing-on-Stone P.P. | Cori Lausen |
| WOS-11 | AB-S |  | 41 | 16-Aug-2001 | m | a | Alberta |  | Canada | Writing-on-Stone P.P. | Cori Lausen |
| WOS-12 | AB-S | KM363924 | 48 | 16-Aug-2001 | m | a | Alberta |  | Canada | Writing-on-Stone P.P. | Cori Lausen |
| WOS-13 | AB-S |  | 41 | 16-Aug-2001 | f | a | Alberta |  | Canada | Writing-on-Stone P.P. | Cori Lausen |
| WOS-14 | AB-S |  | 34 | 16-Aug-2001 | m | a | Alberta |  | Canada | Writing-on-Stone P.P. | Cori Lausen |
| WOS-15 | AB-S |  | 48 | 16-Aug-2001 | m | a | Alberta |  | Canada | Writing-on-Stone P.P. | Cori Lausen |
| WOS-16 | AB-S |  | 34 | 16-Aug-2001 | m | a | Alberta |  | Canada | Writing-on-Stone P.P. | Cori Lausen |
| WOS-17 | AB-S |  | 34 | 16-Aug-2001 | m | a | Alberta |  | Canada | Writing-on-Stone P.P. | Cori Lausen |
| WOS-18 | AB-S |  | 34 | 16-Aug-2001 | f | sa | Alberta |  | Canada | Writing-on-Stone P.P. | Cori Lausen |
| WOS-19 | AB-S | KM363925 | 49 | 16-Aug-2001 | m | a | Alberta |  | Canada | Writing-on-Stone P.P. | Cori Lausen |
| WOS-20 | AB-S |  | 35 | 16-Aug-2001 | m | a | Alberta |  | Canada | Writing-on-Stone P.P. | Cori Lausen |
| WOS-21 | AB-S | KM363926 | 50 | 16-Aug-2001 | f | a | Alberta |  | Canada | Writing-on-Stone P.P. | Cori Lausen |
| WOS-22 | AB-S |  | 34 | 16-Aug-2001 | m | a | Alberta |  | Canada | Writing-on-Stone P.P. | Cori Lausen |
| WOS-23 | AB-S |  | 41 | 16-Aug-2001 | m | a | Alberta |  | Canada | Writing-on-Stone P.P. | Cori Lausen |
| WOS-24 | AB-S |  | 34 | 16-Aug-2001 | f | sa | Alberta |  | Canada | Writing-on-Stone P.P. | Cori Lausen |
| WOS-25 | AB-S |  | 41 | 16-Aug-2001 | m | a | Alberta |  | Canada | Writing-on-Stone P.P. | Cori Lausen |
| WOS-27 | AB-S |  | 48 | 16-Aug-2001 | m | a | Alberta |  | Canada | Writing-on-Stone P.P. | Cori Lausen |
| WOS-28 | AB-S | KM363927 | 51 | 16-Aug-2001 | m | sa | Alberta |  | Canada | Writing-on-Stone P.P. | Cori Lausen |
| WOS-32 | AB-S | KM363928 | 52 | 16-Aug-2001 | m | a | Alberta |  | Canada | Writing-on-Stone P.P. | Cori Lausen |
| WY-68 | WY |  | 53 | 21-Jun-2001 | m | a | Wyoming | Carbon | USA | Medicine Bow Mountains | Jeff Gruver |
| WY-71 | WY |  | 57 | 25-Jun-2001 | m | a | Wyoming | Carbon | USA | Medicine Bow Mountains | Jeff Gruver |
| WY-72 | WY |  | 53 | 25-Jun-2001 | m | a | Wyoming | Carbon | USA | Medicine Bow Mountains | Jeff Gruver |
| WY-75 | WY | KM363935 | 59 | 27-Jun-2001 | m | a | Wyoming | Carbon | USA | Medicine Bow Mountains | Jeff Gruver |
| WY-89 | WY |  | 53 | 2-Jul-2001 | m | a | Wyoming | Carbon | USA | Medicine Bow Mountains | Jeff Gruver |
| WY-98 | WY |  | 53 | 13-Jul-2001 | m | a | Wyoming | Carbon | USA | Medicine Bow Mountains | Jeff Gruver |
| WY-99 | WY |  | 54 | 16-Jul-2001 | m | a | Wyoming | Carbon | USA | Medicine Bow Mountains | Jeff Gruver |
| WY-100 | WY |  | 30 | 16-Jul-2001 | f | a | Wyoming | Carbon | USA | Medicine Bow Mountains | Jeff Gruver |
| WY-102 | WY | KM363929 | 53 | 23-Jul-2001 | m | j | Wyoming | Carbon | USA | Medicine Bow Mountains | Jeff Gruver |
| WY-103 | WY | KM363930 | 54 | 23-Jul-2001 | m | a | Wyoming | Carbon | USA | Medicine Bow Mountains | Jeff Gruver |
| WY-166 | WY |  | 53 | 31-Jul-2001 | m | a | Wyoming | Carbon | USA | Medicine Bow Mountains | Jeff Gruver |
| WY-168 | WY | KM363931 | 55 | 1-Aug-2001 | m | a | Wyoming | Carbon | USA | Medicine Bow Mountains | Jeff Gruver |
| WY-173 | WY | KM363932 | 56 | 2-Aug-2001 | f | a | Wyoming | Carbon | USA | Medicine Bow Mountains | Jeff Gruver |
| WY-180 | WY |  | 55 | 7-Aug-2001 | m | a | Wyoming | Carbon | USA | Medicine Bow Mountains | Jeff Gruver |
| WY-205 | WY | KM363933 | 57 | 8-Aug-2001 | m | j | Wyoming | Carbon | USA | Medicine Bow Mountains | Jeff Gruver |
| WY-208 | WY |  | 53 | 10-Aug-2001 | m | a | Wyoming | Carbon | USA | Medicine Bow Mountains | Jeff Gruver |
| WY-209 | WY |  | 54 | 10-Aug-2001 | f | a | Wyoming | Carbon | USA | Medicine Bow Mountains | Jeff Gruver |
| WY-210 | WY |  | 57 | 10-Aug-2001 | f | a | Wyoming | Carbon | USA | Medicine Bow Mountains | Jeff Gruver |
| WY-213 | WY |  | 41 | 10-Aug-2001 | f | a | Wyoming | Carbon | USA | Medicine Bow Mountains | Jeff Gruver |
| WY-214 | WY |  | 55 | 10-Aug-2001 | f | a | Wyoming | Carbon | USA | Medicine Bow Mountains | Jeff Gruver |
| WY-216 | WY |  | 53 | 10-Aug-2001 | m | j | Wyoming | Carbon | USA | Medicine Bow Mountains | Jeff Gruver |
| WY-217 | WY |  | 54 | 10-Aug-2001 | m | a | Wyoming | Carbon | USA | Medicine Bow Mountains | Jeff Gruver |
| WY-218 | WY |  | 55 | 10-Aug-2001 | m | a | Wyoming | Carbon | USA | Medicine Bow Mountains | Jeff Gruver |
| WY-219 | WY | KM363934 | 58 | 10-Aug-2001 | m | j | Wyoming | Carbon | USA | Medicine Bow Mountains | Jeff Gruver |
| WY-220 | WY |  | 54 | 10-Aug-2001 | m | a | Wyoming | Carbon | USA | Medicine Bow Mountains | Jeff Gruver |
| WY-223 | WY |  | 55 | 10-Aug-2001 | m | j | Wyoming | Carbon | USA | Medicine Bow Mountains | Jeff Gruver |
| WY-249 | WY |  | 54 | 14-Aug-2001 | m | a | Wyoming | Carbon | USA | Medicine Bow Mountains | Jeff Gruver |
| WY-251 | WY |  | 57 | 14-Aug-2001 | f | a | Wyoming | Carbon | USA | Medicine Bow Mountains | Jeff Gruver |
| WY-252 | WY |  | 54 | 14-Aug-2001 | m | a | Wyoming | Carbon | USA | Medicine Bow Mountains | Jeff Gruver |
| GR-01 | MB | KM363936 | 60 | 9-Jun-2010 | m | a | Manitoba |  | Canada | Grand Rapids | Craig Willis |
| GR-02 | MB |  | 60 | 9-Jun-2010 | m | j | Manitoba |  | Canada | Grand Rapids | Craig Willis |
| GR-04 | MB |  | 60 | 9-Jun-2010 | f | a | Manitoba |  | Canada | Grand Rapids | Craig Willis |
| GR-05 | MB |  | 60 | 9-Jun-2010 | f | a | Manitoba |  | Canada | Grand Rapids | Craig Willis |
| GR-06 | MB |  | 34 | 9-Jun-2010 | n | a | Manitoba |  | Canada | Grand Rapids | Craig Willis |
| GR-07 | MB |  | 34 | 9-Jun-2010 | f | a | Manitoba |  | Canada | Grand Rapids | Craig Willis |
| GR-08 | MB |  | 34 | 23-Jul-2011 | f | a | Manitoba |  | Canada | Grand Rapids | Craig Willis |
| GR-09 | MB |  | 60 | 23-Jul-2011 | m | a | Manitoba |  | Canada | Grand Rapids | Craig Willis |
| GR-10 | MB |  | 41 | 23-Jul-2011 | f | j | Manitoba |  | Canada | Grand Rapids | Craig Willis |
| GR-12 | MB |  | 41 | 19-Jun-2009 | m |  | Manitoba |  | Canada | Grand Rapids | Craig Willis |
| GR-13 | MB | KM363937 | 61 | 19-Jun-2009 | m |  | Manitoba |  | Canada | Grand Rapids | Craig Willis |
| SLON01 | ON-1 | KM363938 | 62 | 15-Jul-2011 | f | a | Ontario |  | Canada | Shoal Lake | Craig Willis |
| SLON02 | ON-1 | KM363939 | 63 | 15-Jul-2011 | f | a | Ontario |  | Canada | Shoal Lake | Craig Willis |
| SLON03 | ON-1 | KM363940 | 64 | 15-Jul-2011 | f | j | Ontario |  | Canada | Shoal Lake | Craig Willis |
| SLON04 | ON-1 |  | 64 | 15-Jul-2011 | f | a | Ontario |  | Canada | Shoal Lake | Craig Willis |
| SLON05 | ON-1 | KM363941 | 65 | 15-Jul-2011 | f | a | Ontario |  | Canada | Shoal Lake | Craig Willis |
| SLON06 | ON-1 | KM363942 | 66 | 15-Jul-2011 | f | a | Ontario |  | Canada | Shoal Lake | Craig Willis |
| SLON07 | ON-1 |  | 65 | 15-Jul-2011 | f | a | Ontario |  | Canada | Shoal Lake | Craig Willis |
| SLON08 | ON-1 |  | 64 | 15-Jul-2011 | f | a | Ontario |  | Canada | Shoal Lake | Craig Willis |
| SLON29 | ON-1 |  | 66 | 15-Jul-2011 | m | j | Ontario |  | Canada | Shoal Lake | Craig Willis |
| SLON30 | ON-1 | KM363943 | 67 | 15-Jul-2011 | f | a | Ontario |  | Canada | Shoal Lake | Craig Willis |
| SLON31 | ON-1 |  | 67 | 15-Jul-2011 | f | a | Ontario |  | Canada | Shoal Lake | Craig Willis |
| SLON32 | ON-1 |  | 67 | 15-Jul-2011 | f | a | Ontario |  | Canada | Shoal Lake | Craig Willis |
| SLON33 | ON-1 |  | 68 | 15-Jul-2011 | f | a | Ontario |  | Canada | Shoal Lake | Craig Willis |
| SLON34 | ON-1 | KM363944 | 68 | 15-Jul-2011 | f | a | Ontario |  | Canada | Shoal Lake | Craig Willis |
| SLON35 | ON-1 |  | 67 | 15-Jul-2011 | f | a | Ontario |  | Canada | Shoal Lake | Craig Willis |
| MN3105 | MN |  | 60 | 16-Jun-2010 | m | a | Minnesota | St Louis | USA | Soudan Underground Mine State Park | Gerda Nordquist |
| MN3106 | MN | KM363945 | 69 | 16-Jun-2010 | f | a | Minnesota | St Louis | USA | Soudan Underground Mine State Park | Gerda Nordquist |
| MN3107 | MN | KM363946 | 70 | 16-Jun-2010 | m | a | Minnesota | St Louis | USA | Soudan Underground Mine State Park | Gerda Nordquist |
| MN3108 | MN |  | 34 | 16-Jun-2010 | f | a | Minnesota | St Louis | USA | Soudan Underground Mine State Park | Gerda Nordquist |
| MN3109 | MN |  | 34 | 16-Jun-2010 | f | a | Minnesota | St Louis | USA | Soudan Underground Mine State Park | Gerda Nordquist |
| MN3110 | MN |  | 34 | 16-Jun-2010 | m | a | Minnesota | St Louis | USA | Soudan Underground Mine State Park | Gerda Nordquist |
| MN3111 | MN |  | 35 | 16-Jun-2010 | m | a | Minnesota | St Louis | USA | Soudan Underground Mine State Park | Gerda Nordquist |
| MN3112 | MN |  | 34 | 16-Jun-2010 | m | a | Minnesota | St Louis | USA | Soudan Underground Mine State Park | Gerda Nordquist |
| MN3113 | MN |  | 34 | 16-Jun-2010 | m | a | Minnesota | St Louis | USA | Soudan Underground Mine State Park | Gerda Nordquist |
| MN3114 | MN |  | 34 | 16-Jun-2010 | m | a | Minnesota | St Louis | USA | Soudan Underground Mine State Park | Gerda Nordquist |
| MN3115 | MN |  | 35 | 16-Jun-2010 | m | a | Minnesota | St Louis | USA | Soudan Underground Mine State Park | Gerda Nordquist |
| MN3116 | MN |  | 41 | 16-Jun-2010 | m | a | Minnesota | St Louis | USA | Soudan Underground Mine State Park | Gerda Nordquist |
| MN3117 | MN |  | 35 | 16-Jun-2010 | m | a | Minnesota | St Louis | USA | Soudan Underground Mine State Park | Gerda Nordquist |
| MN3118 | MN |  | 34 | 16-Jun-2010 | f | a | Minnesota | St Louis | USA | Soudan Underground Mine State Park | Gerda Nordquist |
| MN3119 | MN |  | 35 | 16-Jun-2010 | m | a | Minnesota | St Louis | USA | Soudan Underground Mine State Park | Gerda Nordquist |
| MN3120 | MN |  | 34 | 16-Jun-2010 | m | a | Minnesota | St Louis | USA | Soudan Underground Mine State Park | Gerda Nordquist |
| MN3121 | MN | KM363947 | 71 | 16-Jun-2010 | m | a | Minnesota | St Louis | USA | Soudan Underground Mine State Park | Gerda Nordquist |
| MN3122 | MN |  | 41 | 16-Jun-2010 | m | a | Minnesota | St Louis | USA | Soudan Underground Mine State Park | Gerda Nordquist |
| MN3123 | MN |  | 34 | 16-Jun-2010 | m | a | Minnesota | St Louis | USA | Soudan Underground Mine State Park | Gerda Nordquist |
| MN3124 | MN |  | 34 | 16-Jun-2010 | m | a | Minnesota | St Louis | USA | Soudan Underground Mine State Park | Gerda Nordquist |
| DLSP01 | WI-Sa | KM363948 | 72 | 17-Aug-2010 | m | a | Wisconsin | Sauk | USA | Devil's Lake State Park | David Redell |
| DLSP02 | WI-Sa |  | 41 | 17-Aug-2010 | f | j | Wisconsin | Sauk | USA | Devil's Lake State Park | David Redell |
| DLSP03 | WI-Sa |  | 41 | 17-Aug-2010 | f | j | Wisconsin | Sauk | USA | Devil's Lake State Park | David Redell |
| DLSP04 | WI-Sa |  | 41 | 17-Aug-2010 | m | a | Wisconsin | Sauk | USA | Devil's Lake State Park | David Redell |
| DLSP05 | WI-Sa | KM363949 | 73 | 17-Aug-2010 | f | j | Wisconsin | Sauk | USA | Devil's Lake State Park | David Redell |
| DLSP06 | WI-Sa |  | 41 | 17-Aug-2010 | f | a | Wisconsin | Sauk | USA | Devil's Lake State Park | David Redell |
| DLSP07 | WI-Sa |  | 73 | 17-Aug-2010 | f | j | Wisconsin | Sauk | USA | Devil's Lake State Park | David Redell |
| DLSP08 | WI-Sa |  | 35 | 17-Aug-2010 | f | a | Wisconsin | Sauk | USA | Devil's Lake State Park | David Redell |
| DLSP09 | WI-Sa | KM363950 | 74 | 17-Aug-2010 | f | j | Wisconsin | Sauk | USA | Devil's Lake State Park | David Redell |
| DLSP10 | WI-Sa |  | 74 | 17-Aug-2010 | f | j | Wisconsin | Sauk | USA | Devil's Lake State Park | David Redell |
| DLSP11 | WI-Sa |  | 41 | 17-Aug-2010 | m | a | Wisconsin | Sauk | USA | Devil's Lake State Park | David Redell |
| DLSP12 | WI-Sa |  | 41 | 17-Aug-2010 | f | j | Wisconsin | Sauk | USA | Devil's Lake State Park | David Redell |
| DLSP13 | WI-Sa | KM363951 | 75 | 17-Aug-2010 | f | j | Wisconsin | Sauk | USA | Devil's Lake State Park | David Redell |
| DLSP14 | WI-Sa |  | 74 | 17-Aug-2010 | f | a | Wisconsin | Sauk | USA | Devil's Lake State Park | David Redell |
| DLSP15 | WI-Sa |  | 41 | 17-Aug-2010 | f | j | Wisconsin | Sauk | USA | Devil's Lake State Park | David Redell |
| DLSP16 | WI-Sa |  | 34 | 17-Aug-2010 | f | a | Wisconsin | Sauk | USA | Devil's Lake State Park | David Redell |
| DLSP17 | WI-Sa |  | 74 | 17-Aug-2010 | f | a | Wisconsin | Sauk | USA | Devil's Lake State Park | David Redell |
| DLSP18 | WI-Sa |  | 74 | 17-Aug-2010 | f | a | Wisconsin | Sauk | USA | Devil's Lake State Park | David Redell |
| DLSP19 | WI-Sa |  | 75 | 17-Aug-2010 | f | j | Wisconsin | Sauk | USA | Devil's Lake State Park | David Redell |
| DLSP20 | WI-Sa |  | 74 | 17-Aug-2010 | f | a | Wisconsin | Sauk | USA | Devil's Lake State Park | David Redell |
| ENG01 | WI-Ma | KM363952 | 76 | 11-Jun-2010 | f | a | Wisconsin | Marquette | USA | Engleman farm house roost | David Redell |
| ENG02 | WI-Ma |  | 41 | 11-Jun-2010 | f | a | Wisconsin | Marquette | USA | Engleman farm house roost | David Redell |
| ENG03 | WI-Ma |  | 41 | 11-Jun-2010 | f | a | Wisconsin | Marquette | USA | Engleman farm house roost | David Redell |
| ENG04 | WI-Ma | KM363953 | 77 | 11-Jun-2010 | f | a | Wisconsin | Marquette | USA | Engleman farm house roost | David Redell |
| ENG05 | WI-Ma |  | 41 | 11-Jun-2010 | f | a | Wisconsin | Marquette | USA | Engleman farm house roost | David Redell |
| ENG06 | WI-Ma |  | 77 | 11-Jun-2010 | f | a | Wisconsin | Marquette | USA | Engleman farm house roost | David Redell |
| ENG07 | WI-Ma |  | 36 | 11-Jun-2010 | f | a | Wisconsin | Marquette | USA | Engleman farm house roost | David Redell |
| ENG08 | WI-Ma |  | 36 | 11-Jun-2010 | f | a | Wisconsin | Marquette | USA | Engleman farm house roost | David Redell |
| ENG09 | WI-Ma |  | 41 | 11-Jun-2010 | f | a | Wisconsin | Marquette | USA | Engleman farm house roost | David Redell |
| ENG10 | WI-Ma |  | 77 | 11-Jun-2010 | f | a | Wisconsin | Marquette | USA | Engleman farm house roost | David Redell |
| ENG11 | WI-Ma | KM363954 | 78 | 11-Jun-2010 | f | a | Wisconsin | Marquette | USA | Engleman farm house roost | David Redell |
| ENG12 | WI-Ma | KM363955 | 79 | 11-Jun-2010 | f | a | Wisconsin | Marquette | USA | Engleman farm house roost | David Redell |
| ENG13 | WI-Ma |  | 36 | 11-Jun-2010 | f | a | Wisconsin | Marquette | USA | Engleman farm house roost | David Redell |
| ENG14 | WI-Ma |  | 35 | 11-Jun-2010 | f | a | Wisconsin | Marquette | USA | Engleman farm house roost | David Redell |
| ENG15 | WI-Ma | KM363956 | 80 | 11-Jun-2010 | m | a | Wisconsin | Marquette | USA | Engleman farm house roost | David Redell |
| ENG16 | WI-Ma |  | 41 | 11-Jun-2010 | f | a | Wisconsin | Marquette | USA | Engleman farm house roost | David Redell |
| ENG17 | WI-Ma |  | 80 | 11-Jun-2010 | f | a | Wisconsin | Marquette | USA | Engleman farm house roost | David Redell |
| ENG18 | WI-Ma |  | 80 | 11-Jun-2010 | f | a | Wisconsin | Marquette | USA | Engleman farm house roost | David Redell |
| ENG19 | WI-Ma | KM363957 | 81 | 11-Jun-2010 | f | a | Wisconsin | Marquette | USA | Engleman farm house roost | David Redell |
| ENG20 | WI-Ma |  | 41 | 11-Jun-2010 | f | a | Wisconsin | Marquette | USA | Engleman farm house roost | David Redell |
| MV-168 | MI |  | n/a | 1-JUL-2008 | f | a | Michigan | Cass | USA | Crystal Springs United Methodist Church Camp | Maarten Vonhof |
| MV-169 | MI |  | n/a | 1-JUL-2008 | f | a | Michigan | Cass | USA | Crystal Springs United Methodist Church Camp | Maarten Vonhof |
| MV-170 | MI |  | n/a | 1-JUL-2008 | f | a | Michigan | Cass | USA | Crystal Springs United Methodist Church Camp | Maarten Vonhof |
| MV-171 | MI |  | n/a | 1-JUL-2008 | f | a | Michigan | Cass | USA | Crystal Springs United Methodist Church Camp | Maarten Vonhof |
| MV-172 | MI |  | n/a | 1-JUL-2008 | f | a | Michigan | Cass | USA | Crystal Springs United Methodist Church Camp | Maarten Vonhof |
| MV-173 | MI |  | n/a | 1-JUL-2008 | m | a | Michigan | Cass | USA | Crystal Springs United Methodist Church Camp | Maarten Vonhof |
| MV-174 | MI |  | n/a | 1-JUL-2008 | m | a | Michigan | Cass | USA | Crystal Springs United Methodist Church Camp | Maarten Vonhof |
| MV-175 | MI |  | n/a | 1-JUL-2008 | m | a | Michigan | Cass | USA | Crystal Springs United Methodist Church Camp | Maarten Vonhof |
| MV-176 | MI |  | n/a | 1-JUL-2008 | m | a | Michigan | Cass | USA | Crystal Springs United Methodist Church Camp | Maarten Vonhof |
| MV-177 | MI |  | n/a | 1-JUL-2008 | m | a | Michigan | Cass | USA | Crystal Springs United Methodist Church Camp | Maarten Vonhof |
| MV-178 | MI |  | n/a | 1-JUL-2008 | m | j | Michigan | Cass | USA | Crystal Springs United Methodist Church Camp | Maarten Vonhof |
| MV-179 | MI |  | n/a | 1-JUL-2008 | m | j | Michigan | Cass | USA | Crystal Springs United Methodist Church Camp | Maarten Vonhof |
| MV-180 | MI |  | n/a | 1-JUL-2008 | m | j | Michigan | Cass | USA | Crystal Springs United Methodist Church Camp | Maarten Vonhof |
| MV-181 | MI |  | n/a | 1-JUL-2008 | m | j | Michigan | Cass | USA | Crystal Springs United Methodist Church Camp | Maarten Vonhof |
| MV-182 | MI |  | n/a | 1-JUL-2008 | f | j | Michigan | Cass | USA | Crystal Springs United Methodist Church Camp | Maarten Vonhof |
| MV-183 | MI |  | n/a | 1-JUL-2008 | f | j | Michigan | Cass | USA | Crystal Springs United Methodist Church Camp | Maarten Vonhof |
| MV-184 | MI |  | n/a | 1-JUL-2008 | f | j | Michigan | Cass | USA | Crystal Springs United Methodist Church Camp | Maarten Vonhof |
| MV-185 | MI |  | n/a | 1-JUL-2008 | f | j | Michigan | Cass | USA | Crystal Springs United Methodist Church Camp | Maarten Vonhof |
| MV-186 | MI |  | n/a | 1-JUL-2008 | f | j | Michigan | Cass | USA | Crystal Springs United Methodist Church Camp | Maarten Vonhof |
| MV-187 | MI |  | n/a | 1-JUL-2008 | f | j | Michigan | Cass | USA | Crystal Springs United Methodist Church Camp | Maarten Vonhof |
| EB-122 | KY | KM363958 | 82 | 24-Jun-2002 | m | a | Kentucky | Rowan | USA | Bangor | Eric Britzke |
| EB-131 | KY | KM363959 | 83 | 26-Jun-2002 | f | a | Kentucky | Rowan | USA | Bangor | Eric Britzke |
| EB-132 | KY | KM363960 | 84 | 26-Jun-2002 | f | a | Kentucky | Rowan | USA | Bangor | Eric Britzke |
| EB-133 | KY |  | 83 | 26-Jun-2002 | f | a | Kentucky | Rowan | USA | Bangor | Eric Britzke |
| EB-134 | KY | KM363961 | 85 | 26-Jun-2002 | f | a | Kentucky | Rowan | USA | Bangor | Eric Britzke |
| EB-135 | KY |  | 85 | 26-Jun-2002 | f | a | Kentucky | Rowan | USA | Bangor | Eric Britzke |
| EB-136 | KY |  | 83 | 26-Jun-2002 | f | a | Kentucky | Rowan | USA | Bangor | Eric Britzke |
| EB-137 | KY |  | 84 | 26-Jun-2002 | f | a | Kentucky | Rowan | USA | Bangor | Eric Britzke |
| EB-138 | KY |  | 85 | 26-Jun-2002 | f | a | Kentucky | Rowan | USA | Bangor | Eric Britzke |
| EB-139 | KY | KM363962 | 86 | 26-Jun-2002 | f | a | Kentucky | Rowan | USA | Bangor | Eric Britzke |
| EB-140 | KY | KM363963 | 87 | 26-Jun-2002 | f | a | Kentucky | Rowan | USA | Bangor | Eric Britzke |
| EB-141 | KY |  | 83 | 26-Jun-2002 | f | a | Kentucky | Rowan | USA | Bangor | Eric Britzke |
| EB-142 | KY |  | 83 | 26-Jun-2002 | m | a | Kentucky | Rowan | USA | Bangor | Eric Britzke |
| EB-143 | KY |  | 83 | 26-Jun-2002 | f | a | Kentucky | Rowan | USA | Bangor | Eric Britzke |
| EB-144 | KY |  | 83 | 26-Jun-2002 | f | a | Kentucky | Rowan | USA | Bangor | Eric Britzke |
| EB-145 | KY |  | 87 | 26-Jun-2002 | f | a | Kentucky | Rowan | USA | Bangor | Eric Britzke |
| EB-146 | KY |  | 83 | 26-Jun-2002 | f | a | Kentucky | Rowan | USA | Bangor | Eric Britzke |
| EB-147 | KY |  | 83 | 26-Jun-2002 | f | a | Kentucky | Rowan | USA | Bangor | Eric Britzke |
| EB-148 | KY |  | 83 | 26-Jun-2002 | f | a | Kentucky | Rowan | USA | Bangor | Eric Britzke |
| EB-150 | KY | KM363964 | 88 | 26-Jun-2002 | f | a | Kentucky | Rowan | USA | Bangor | Eric Britzke |
| EB-151 | KY | KM363965 | 89 | 26-Jun-2002 | f | a | Kentucky | Rowan | USA | Bangor | Eric Britzke |
| EB-152 | KY |  | 89 | 26-Jun-2002 | f | a | Kentucky | Rowan | USA | Bangor | Eric Britzke |
| EB-155 | KY |  | 84 | 29-Jun-2002 | m | a | Kentucky | Rowan | USA | Bangor | Eric Britzke |
| EB-173 | KY | KM363966 | 90 | 1-Aug-2002 | m | a | Kentucky | Rowan | USA | Morehead | Eric Britzke |
| EB-191 | KY | KM363967 | 91 | 12-Aug-2002 | m | a | Kentucky | Rowan | USA | Morehead | Eric Britzke |
| EB-60 | KY | KM363968 | 92 | 4-Jun-2002 | m | a | Kentucky | Rowan | USA | Bangor | Eric Britzke |
| EB-94 | KY |  | 83 | 21-Jun-2002 | m | a | Kentucky | Rowan | USA | Bangor | Eric Britzke |
| EKPC-04 | KY |  | 35 | 9-Aug-2002 | f | a | Kentucky | Rowan | USA | Morehead | Mark Gumbert |
| EKPC-19 | KY |  | 76 | 10-Aug-2002 | f | a | Kentucky | Rowan | USA | Morehead | Mark Gumbert |
| Rush-02 | OH | KM363969 | 93 | 9-Jul-2006 | m | j | Ohio | Fairfield | USA | Rushville | Lee Johnson |
| Rush-03 | OH |  | 35 | 9-Jul-2006 | m | j | Ohio | Fairfield | USA | Rushville | Lee Johnson |
| Rush-04 | OH | KM363970 | 94 | 9-Jul-2006 | m | j | Ohio | Fairfield | USA | Rushville | Lee Johnson |
| Rush-06 | OH |  | 35 | 9-Jul-2006 | f | j | Ohio | Fairfield | USA | Rushville | Lee Johnson |
| Rush-07 | OH | KM363971 | 95 | 9-Jul-2006 | f | j | Ohio | Fairfield | USA | Rushville | Lee Johnson |
| Rush-08 | OH | KM363972 | 96 | 9-Jul-2006 | f | j | Ohio | Fairfield | USA | Rushville | Lee Johnson |
| Rush-09 | OH |  | 35 | 9-Jul-2006 | f | j | Ohio | Fairfield | USA | Rushville | Lee Johnson |
| Rush-10 | OH |  | 35 | 9-Jul-2006 | m | j | Ohio | Fairfield | USA | Rushville | Lee Johnson |
| Rush-11 | OH |  | 93 | 9-Jul-2006 | f | j | Ohio | Fairfield | USA | Rushville | Lee Johnson |
| Rush-12 | OH | KM363973 | 97 | 9-Jul-2006 | m | j | Ohio | Fairfield | USA | Rushville | Lee Johnson |
| Rush-13 | OH | KM363974 | 98 | 9-Jul-2006 | f | j | Ohio | Fairfield | USA | Rushville | Lee Johnson |
| Rush-14 | OH |  | 35 | 9-Jul-2006 | f | j | Ohio | Fairfield | USA | Rushville | Lee Johnson |
| Rush-15 | OH |  | 96 | 9-Jul-2006 | f | j | Ohio | Fairfield | USA | Rushville | Lee Johnson |
| Rush-16 | OH |  | 93 | 9-Jul-2006 | f | j | Ohio | Fairfield | USA | Rushville | Lee Johnson |
| Rush-17 | OH |  | 35 | 9-Jul-2006 | f | j | Ohio | Fairfield | USA | Rushville | Lee Johnson |
| Rush-18 | OH |  | 93 | 9-Jul-2006 | f | j | Ohio | Fairfield | USA | Rushville | Lee Johnson |
| Rush-20 | OH | KM363975 | 99 | 9-Jul-2006 | f | j | Ohio | Fairfield | USA | Rushville | Lee Johnson |
| Rush-24 | OH |  | 93 | 9-Jul-2006 | f | a | Ohio | Fairfield | USA | Rushville | Lee Johnson |
| Rush-27 | OH | KM363976 | 100 | 9-Jul-2006 | f | a | Ohio | Fairfield | USA | Rushville | Lee Johnson |
| Rush-28 | OH |  | 95 | 9-Jul-2006 | f | a | Ohio | Fairfield | USA | Rushville | Lee Johnson |
| Rush-29 | OH |  | 94 | 9-Jul-2006 | f | a | Ohio | Fairfield | USA | Rushville | Lee Johnson |
| Rush-30 | OH |  | 41 | 9-Jul-2006 | f | a | Ohio | Fairfield | USA | Rushville | Lee Johnson |
| Rush-31 | OH |  | 100 | 9-Jul-2006 | f | a | Ohio | Fairfield | USA | Rushville | Lee Johnson |
| Rush-32 | OH |  | 94 | 9-Jul-2006 | f | a | Ohio | Fairfield | USA | Rushville | Lee Johnson |
| Rush-33 | OH |  | 35 | 9-Jul-2006 | f | a | Ohio | Fairfield | USA | Rushville | Lee Johnson |
| Rush-35 | OH |  | 93 | 9-Jul-2006 | f | a | Ohio | Fairfield | USA | Rushville | Lee Johnson |
| Rush-36 | OH |  | 41 | 9-Jul-2006 | f | a | Ohio | Fairfield | USA | Rushville | Lee Johnson |
| Rush-37 | OH |  | 35 | 9-Jul-2006 | f | a | Ohio | Fairfield | USA | Rushville | Lee Johnson |
| Rush-38 | OH |  | 74 | 9-Jul-2006 | f | a | Ohio | Fairfield | USA | Rushville | Lee Johnson |
| Rush-40 | OH |  | 94 | 9-Jul-2006 | f | a | Ohio | Fairfield | USA | Rushville | Lee Johnson |
| Rush-42 | OH |  | 74 | 9-Jul-2006 | f | a | Ohio | Fairfield | USA | Rushville | Lee Johnson |
| SMNP-113 | TN |  | 36 | 19-Jun-2002 | f | a | Tennessee | Blount | USA | Great Smoky Mountains National Park | GSMNP |
| SMNP-115 | TN |  | 36 | 19-Jun-2002 | f | a | Tennessee | Blount | USA | Great Smoky Mountains National Park | GSMNP |
| SMNP-118 | TN |  | 74 | 19-Jun-2002 | f | a | Tennessee | Blount | USA | Great Smoky Mountains National Park | GSMNP |
| SMNP-122 | TN |  | 36 | 20-Jun-2002 | m | a | Tennessee | Blount | USA | Great Smoky Mountains National Park | GSMNP |
| SMNP-31 | TN |  | 36 | 19-Jun-2002 | f | a | Tennessee | Blount | USA | Great Smoky Mountains National Park | GSMNP |
| SMNP-37 | TN |  | 36 | 19-Jun-2002 | f | j | Tennessee | Blount | USA | Great Smoky Mountains National Park | GSMNP |
| SMNP-38 | TN |  | 74 | 19-Jun-2002 | f | a | Tennessee | Blount | USA | Great Smoky Mountains National Park | GSMNP |
| SMNP-39 | TN |  | 36 | 19-Jun-2002 | f | a | Tennessee | Blount | USA | Great Smoky Mountains National Park | GSMNP |
| SMNP-40 | TN |  | 36 | 19-Jun-2002 | f | j | Tennessee | Blount | USA | Great Smoky Mountains National Park | GSMNP |
| SMNP-41 | TN |  | 36 | 19-Jun-2002 | f | a | Tennessee | Blount | USA | Great Smoky Mountains National Park | GSMNP |
| SMNP-42 | TN |  | 36 | 19-Jun-2002 | f | a | Tennessee | Blount | USA | Great Smoky Mountains National Park | GSMNP |
| SMNP-43 | TN |  | 36 | 19-Jun-2002 | f | a | Tennessee | Blount | USA | Great Smoky Mountains National Park | GSMNP |
| SMNP-44 | TN |  | 36 | 19-Jun-2002 | f | a | Tennessee | Blount | USA | Great Smoky Mountains National Park | GSMNP |
| SMNP-45 | TN |  | 74 | 19-Jun-2002 | f | a | Tennessee | Blount | USA | Great Smoky Mountains National Park | GSMNP |
| SMNP-46 | TN |  | 74 | 19-Jun-2002 | f | a | Tennessee | Blount | USA | Great Smoky Mountains National Park | GSMNP |
| SMNP-47 | TN |  | 36 | 19-Jun-2002 | f | j | Tennessee | Blount | USA | Great Smoky Mountains National Park | GSMNP |
| SMNP-48 | TN |  | 36 | 19-Jun-2002 | f | a | Tennessee | Blount | USA | Great Smoky Mountains National Park | GSMNP |
| SMNP-49 | TN |  | 36 | 19-Jun-2002 | f | a | Tennessee | Blount | USA | Great Smoky Mountains National Park | GSMNP |
| SMNP-50 | TN |  | 74 | 19-Jun-2002 | f | a | Tennessee | Blount | USA | Great Smoky Mountains National Park | GSMNP |
| SMNP-51 | TN |  | 36 | 19-Jun-2002 | f | a | Tennessee | Blount | USA | Great Smoky Mountains National Park | GSMNP |
| SMNP-52 | TN |  | 36 | 19-Jun-2002 | f | a | Tennessee | Blount | USA | Great Smoky Mountains National Park | GSMNP |
| SMNP-56 | TN |  | 36 | 17-Jun-2002 | m | a | Tennessee | Blount | USA | Great Smoky Mountains National Park | GSMNP |
| SMNP-57 | TN | KM363977 | 101 | 17-Jun-2002 | m | a | Tennessee | Blount | USA | Great Smoky Mountains National Park | GSMNP |
| SMNP-66 | TN |  | 36 | 17-Jun-2002 | m | a | Tennessee | Blount | USA | Great Smoky Mountains National Park | GSMNP |
| SMNP-67 | TN |  | 79 | 17-Jun-2002 | m | a | Tennessee | Blount | USA | Great Smoky Mountains National Park | GSMNP |
| SMNP-68 | TN |  | 36 | 17-Jun-2002 | m | a | Tennessee | Blount | USA | Great Smoky Mountains National Park | GSMNP |
| SMNP-71 | TN |  | 36 | 18-Jun-2002 | m | a | Tennessee | Blount | USA | Great Smoky Mountains National Park | GSMNP |
| SMNP-72 | TN |  | 36 | 18-Jun-2002 | m | a | Tennessee | Blount | USA | Great Smoky Mountains National Park | GSMNP |
| SMNP-75 | TN |  | 101 | 17-Jun-2002 | m | a | Tennessee | Blount | USA | Great Smoky Mountains National Park | GSMNP |
| SMNP-76 | TN |  | 101 | 17-Jun-2002 | m | a | Tennessee | Blount | USA | Great Smoky Mountains National Park | GSMNP |
| SMNP-77 | TN |  | 36 | 17-Jun-2002 | m | a | Tennessee | Blount | USA | Great Smoky Mountains National Park | GSMNP |
| SMNP-99 | TN |  | 36 | 17-Jun-2002 | m | a | Tennessee | Blount | USA | Great Smoky Mountains National Park | GSMNP |
| GV-01 | WV |  | 79 | 23-Aug-2010 | f | a | West Virginia | Raleigh | USA | Grandview bat house | Craig Stihler |
| GV-02 | WV | KM363978 | 102 | 23-Aug-2010 | f | a | West Virginia | Raleigh | USA | Grandview bat house | Craig Stihler |
| GV-03 | WV |  | 35 | 23-Aug-2010 | f | a | West Virginia | Raleigh | USA | Grandview bat house | Craig Stihler |
| GV-04 | WV |  | 79 | 23-Aug-2010 | f | a | West Virginia | Raleigh | USA | Grandview bat house | Craig Stihler |
| GV-05 | WV | KM363979 | 103 | 23-Aug-2010 | f | a | West Virginia | Raleigh | USA | Grandview bat house | Craig Stihler |
| GV-06 | WV |  | 102 | 23-Aug-2010 | f | a | West Virginia | Raleigh | USA | Grandview bat house | Craig Stihler |
| GV-07 | WV | KM363980 | 104 | 23-Aug-2010 | f | a | West Virginia | Raleigh | USA | Grandview bat house | Craig Stihler |
| GV-08 | WV |  | 102 | 23-Aug-2010 | f | a | West Virginia | Raleigh | USA | Grandview bat house | Craig Stihler |
| GV-09 | WV |  | 36 | 23-Aug-2010 | f | a | West Virginia | Raleigh | USA | Grandview bat house | Craig Stihler |
| GV-10 | WV |  | 102 | 23-Aug-2010 | f | a | West Virginia | Raleigh | USA | Grandview bat house | Craig Stihler |
| GV-11 | WV |  | 36 | 23-Aug-2010 | f | a | West Virginia | Raleigh | USA | Grandview bat house | Craig Stihler |
| GV-12 | WV |  | 36 | 23-Aug-2010 | f | a | West Virginia | Raleigh | USA | Grandview bat house | Craig Stihler |
| GV-13 | WV |  | 36 | 23-Aug-2010 | f | a | West Virginia | Raleigh | USA | Grandview bat house | Craig Stihler |
| GV-14 | WV |  | 36 | 23-Aug-2010 | f | a | West Virginia | Raleigh | USA | Grandview bat house | Craig Stihler |
| GV-15 | WV |  | 102 | 23-Aug-2010 | f | a | West Virginia | Raleigh | USA | Grandview bat house | Craig Stihler |
| GV-16 | WV |  | 36 | 23-Aug-2010 | f | a | West Virginia | Raleigh | USA | Grandview bat house | Craig Stihler |
| GV-17 | WV | KM363981 | 105 | 23-Aug-2010 | f | a | West Virginia | Raleigh | USA | Grandview bat house | Craig Stihler |
| GV-18 | WV |  | 102 | 23-Aug-2010 | f | a | West Virginia | Raleigh | USA | Grandview bat house | Craig Stihler |
| GV-19 | WV |  | 36 | 23-Aug-2010 | f | a | West Virginia | Raleigh | USA | Grandview bat house | Craig Stihler |
| GV-20 | WV |  | 36 | 23-Aug-2010 | f | a | West Virginia | Raleigh | USA | Grandview bat house | Craig Stihler |
| PB083 | ON-2 |  | 34 | 1-May-2007 |  |  | Ontario |  | Canada | Port Rowan | Elizabeth Clare |
| PB086 | ON-2 | KM363982 | 106 | 1-May-2007 |  |  | Ontario |  | Canada | Port Rowan | Elizabeth Clare |
| PB091 | ON-2 |  | 36 | 1-May-2007 |  |  | Ontario |  | Canada | Port Rowan | Elizabeth Clare |
| PB092 | ON-2 | KM363983 | 107 | 1-May-2007 |  |  | Ontario |  | Canada | Port Rowan | Elizabeth Clare |
| PB097 | ON-2 |  | 79 | 1-May-2007 |  |  | Ontario |  | Canada | Port Rowan | Elizabeth Clare |
| PB099 | ON-2 |  | 94 | 1-May-2007 |  |  | Ontario |  | Canada | Port Rowan | Elizabeth Clare |
| PB102 | ON-2 |  | 93 | 1-May-2007 |  |  | Ontario |  | Canada | Port Rowan | Elizabeth Clare |
| PB104 | ON-2 |  | 36 | 1-May-2007 |  |  | Ontario |  | Canada | Port Rowan | Elizabeth Clare |
| PB110 | ON-2 |  | 36 | 1-May-2007 |  |  | Ontario |  | Canada | Port Rowan | Elizabeth Clare |
| PB112 | ON-2 |  | 36 | 1-May-2007 |  |  | Ontario |  | Canada | Port Rowan | Elizabeth Clare |
| PB116 | ON-2 | KM363984 | 108 | 1-May-2007 |  |  | Ontario |  | Canada | Port Rowan | Elizabeth Clare |
| PB118 | ON-2 |  | 36 | 1-May-2007 |  |  | Ontario |  | Canada | Port Rowan | Elizabeth Clare |
| PB122 | ON-2 |  | 36 | 1-May-2007 |  |  | Ontario |  | Canada | Port Rowan | Elizabeth Clare |
| PB124 | ON-2 |  | 35 | 1-May-2007 |  |  | Ontario |  | Canada | Port Rowan | Elizabeth Clare |
| PB126 | ON-2 |  | 36 | 1-May-2007 |  |  | Ontario |  | Canada | Port Rowan | Elizabeth Clare |
| PB130 | ON-2 | KM363985 | 109 | 1-May-2007 |  |  | Ontario |  | Canada | Port Rowan | Elizabeth Clare |
| PB132 | ON-2 |  | 36 | 1-May-2007 |  |  | Ontario |  | Canada | Port Rowan | Elizabeth Clare |
| PB134 | ON-2 |  | 94 | 1-May-2007 |  |  | Ontario |  | Canada | Port Rowan | Elizabeth Clare |
| PB138 | ON-2 |  | 36 | 1-May-2007 |  |  | Ontario |  | Canada | Port Rowan | Elizabeth Clare |
| PB140 | ON-2 |  | 36 | 1-May-2007 |  |  | Ontario |  | Canada | Port Rowan | Elizabeth Clare |
| PB142 | ON-2 |  | 109 | 1-May-2007 |  |  | Ontario |  | Canada | Port Rowan | Elizabeth Clare |
| PB144 | ON-2 |  | 35 | 1-May-2007 |  |  | Ontario |  | Canada | Port Rowan | Elizabeth Clare |
| PB146 | ON-2 |  | 34 | 1-May-2007 |  |  | Ontario |  | Canada | Port Rowan | Elizabeth Clare |
| PB148 | ON-2 |  | 74 | 1-May-2007 |  |  | Ontario |  | Canada | Port Rowan | Elizabeth Clare |
| PB150 | ON-2 |  | 36 | 1-May-2007 |  |  | Ontario |  | Canada | Port Rowan | Elizabeth Clare |
| PB152 | ON-2 |  | 35 | 1-May-2007 |  |  | Ontario |  | Canada | Port Rowan | Elizabeth Clare |
| PB154 | ON-2 | KM363986 | 110 | 1-May-2007 |  |  | Ontario |  | Canada | Port Rowan | Elizabeth Clare |
| PB156 | ON-2 |  | 36 | 1-May-2007 |  |  | Ontario |  | Canada | Port Rowan | Elizabeth Clare |
| PB159 | ON-2 |  | 36 | 1-May-2007 |  |  | Ontario |  | Canada | Port Rowan | Elizabeth Clare |
| PB160 | ON-2 |  | 34 | 1-May-2007 |  |  | Ontario |  | Canada | Port Rowan | Elizabeth Clare |
| CCC-02 | PA | KM363987 | 111 | 30-May-2006 | f | a | Pennsylvania | Blair | USA | Canoe Creek State Park | Cal Butchkoscki |
| CCC-04 | PA |  | 36 | 30-May-2006 | f | a | Pennsylvania | Blair | USA | Canoe Creek State Park | Cal Butchkoscki |
| CCC-05 | PA |  | 36 | 30-May-2006 | f | a | Pennsylvania | Blair | USA | Canoe Creek State Park | Cal Butchkoscki |
| CCC-06 | PA |  | 36 | 30-May-2006 | f | a | Pennsylvania | Blair | USA | Canoe Creek State Park | Cal Butchkoscki |
| CCC-07 | PA | KM363988 | 112 | 30-May-2006 | f | a | Pennsylvania | Blair | USA | Canoe Creek State Park | Cal Butchkoscki |
| CCC-08 | PA |  | 110 | 30-May-2006 | f | a | Pennsylvania | Blair | USA | Canoe Creek State Park | Cal Butchkoscki |
| CCC-09 | PA |  | 93 | 30-May-2006 | f | a | Pennsylvania | Blair | USA | Canoe Creek State Park | Cal Butchkoscki |
| CCC-10 | PA |  | 36 | 30-May-2006 | f | a | Pennsylvania | Blair | USA | Canoe Creek State Park | Cal Butchkoscki |
| CCC-11 | PA | KM363989 | 113 | 30-May-2006 | f | a | Pennsylvania | Blair | USA | Canoe Creek State Park | Cal Butchkoscki |
| CCC-12 | PA |  | 113 | 30-May-2006 | f | a | Pennsylvania | Blair | USA | Canoe Creek State Park | Cal Butchkoscki |
| CCC-13 | PA |  | 113 | 30-May-2006 | f | a | Pennsylvania | Blair | USA | Canoe Creek State Park | Cal Butchkoscki |
| CCC-14 | PA | KM363990 | 114 | 30-May-2006 | f | a | Pennsylvania | Blair | USA | Canoe Creek State Park | Cal Butchkoscki |
| CCC-15 | PA | KM363991 | 115 | 30-May-2006 | f | a | Pennsylvania | Blair | USA | Canoe Creek State Park | Cal Butchkoscki |
| CCC-16 | PA | KM363992 | 116 | 30-May-2006 | f | a | Pennsylvania | Blair | USA | Canoe Creek State Park | Cal Butchkoscki |
| CCC-17 | PA |  | 110 | 30-May-2006 | f | a | Pennsylvania | Blair | USA | Canoe Creek State Park | Cal Butchkoscki |
| CCC-18 | PA |  | 36 | 30-May-2006 | f | a | Pennsylvania | Blair | USA | Canoe Creek State Park | Cal Butchkoscki |
| CCC-19 | PA |  | 36 | 30-May-2006 | f | a | Pennsylvania | Blair | USA | Canoe Creek State Park | Cal Butchkoscki |
| CCC-20 | PA | KM363993 | 117 | 30-May-2006 | f | a | Pennsylvania | Blair | USA | Canoe Creek State Park | Cal Butchkoscki |
| CCC-21 | PA |  | 79 | 30-May-2006 | f | a | Pennsylvania | Blair | USA | Canoe Creek State Park | Cal Butchkoscki |
| CCC-22 | PA |  | 36 | 30-May-2006 | f | a | Pennsylvania | Blair | USA | Canoe Creek State Park | Cal Butchkoscki |
| CCC-25 | PA | KM363994 | 118 | 30-May-2006 | f | a | Pennsylvania | Blair | USA | Canoe Creek State Park | Cal Butchkoscki |
| JJ-03 | MD | KM363995 | 119 | 17-Jun-2004 | m | a | Maryland | Washington | USA | Antietam National Battlefield | Josh Johnson |
| JJ-61 | MD |  | 116 | 15-Jul-2004 | m | a | Maryland | Washington | USA | Chesapeake and Ohio Canal National Historical Park | Josh Johnson |
| JJ-85 | MD |  | 36 | 9-Aug-2004 | f | j | Maryland | Washington | USA | Chesapeake and Ohio Canal National Historical Park | Josh Johnson |
| JJ-86 | MD | KM363996 | 120 | 9-Aug-2004 | f | a | Maryland | Washington | USA | Chesapeake and Ohio Canal National Historical Park | Josh Johnson |
| JJ-87 | MD |  | 120 | 9-Aug-2004 | f | a | Maryland | Washington | USA | Chesapeake and Ohio Canal National Historical Park | Josh Johnson |
| JJ-88 | MD |  | 36 | 9-Aug-2004 | f | a | Maryland | Washington | USA | Chesapeake and Ohio Canal National Historical Park | Josh Johnson |
| JJ-89 | MD | KM363997 | 121 | 9-Aug-2004 | m | a | Maryland | Washington | USA | Chesapeake and Ohio Canal National Historical Park | Josh Johnson |
| JJ-90 | MD | KM363998 | 122 | 9-Aug-2004 | f | a | Maryland | Washington | USA | Chesapeake and Ohio Canal National Historical Park | Josh Johnson |
| JJ-91 | MD | KM363999 | 123 | 9-Aug-2004 | f | a | Maryland | Washington | USA | Chesapeake and Ohio Canal National Historical Park | Josh Johnson |
| JJ-92 | MD |  | 102 | 9-Aug-2004 | f | a | Maryland | Washington | USA | Chesapeake and Ohio Canal National Historical Park | Josh Johnson |
| JJ-93 | MD |  | 120 | 9-Aug-2004 | f | a | Maryland | Washington | USA | Chesapeake and Ohio Canal National Historical Park | Josh Johnson |
| JJ-94 | MD |  | 116 | 9-Aug-2004 | m | j | Maryland | Washington | USA | Chesapeake and Ohio Canal National Historical Park | Josh Johnson |
| JJ-95 | MD |  | 122 | 9-Aug-2004 | m | j | Maryland | Washington | USA | Chesapeake and Ohio Canal National Historical Park | Josh Johnson |
| JJ-96 | MD |  | 36 | 9-Aug-2004 | m | a | Maryland | Washington | USA | Chesapeake and Ohio Canal National Historical Park | Josh Johnson |
| JJ-97 | MD |  | 96 | 9-Aug-2004 | m | a | Maryland | Washington | USA | Chesapeake and Ohio Canal National Historical Park | Josh Johnson |
| JJ-98 | MD | KM364000 | 124 | 9-Aug-2004 | m | a | Maryland | Washington | USA | Chesapeake and Ohio Canal National Historical Park | Josh Johnson |
| JJ-110 | MD |  | 36 | 9-Aug-2004 | m | a | Maryland | Washington | USA | Western Maryland | Josh Johnson |
| JJ-111 | MD |  | 120 | 9-Aug-2004 | m | a | Maryland | Washington | USA | Western Maryland | Josh Johnson |
| JJ-113 | MD |  | 36 | 10-Aug-2004 | f | j | Maryland | Washington | USA | Chesapeake and Ohio Canal National Historical Park | Josh Johnson |
| JJ-114 | MD |  | 74 | 11-Aug-2004 | m | a | Maryland | Washington | USA | Chesapeake and Ohio Canal National Historical Park | Josh Johnson |
| JJ-137 | MD |  | 116 | 19-Aug-2004 | f | a | Maryland | Washington | USA | Antietam National Battlefield | Josh Johnson |
| JJ-138 | MD |  | 36 | 19-Aug-2004 | m | a | Maryland | Washington | USA | Antietam National Battlefield | Josh Johnson |
| JJ-139 | MD | KM364001 | 125 | 19-Aug-2004 | f | a | Maryland | Washington | USA | Antietam National Battlefield | Josh Johnson |
| JJ-140 | MD | KM364002 | 126 | 19-Aug-2004 | f | a | Maryland | Washington | USA | Antietam National Battlefield | Josh Johnson |
| JJ-144 | MD |  | 120 | 19-Aug-2004 | f | a | Maryland | Washington | USA | Antietam National Battlefield | Josh Johnson |
| JJ-146 | MD |  | 36 | 19-Aug-2004 | f | a | Maryland | Washington | USA | Antietam National Battlefield | Josh Johnson |
| JJ-150 | MD |  | 36 | 19-Aug-2004 | m | a | Maryland | Washington | USA | Antietam National Battlefield | Josh Johnson |
| JJ-151 | MD |  | 120 | 19-Aug-2004 | f | j | Maryland | Washington | USA | Antietam National Battlefield | Josh Johnson |
| JJ-152 | MD | KM364003 | 127 | 19-Aug-2004 | f | a | Maryland | Washington | USA | Antietam National Battlefield | Josh Johnson |
| JJ-153 | MD |  | 127 | 19-Aug-2004 | f | a | Maryland | Washington | USA | Antietam National Battlefield | Josh Johnson |
| JJ-154 | MD |  | 122 | 19-Aug-2004 | f | a | Maryland | Washington | USA | Antietam National Battlefield | Josh Johnson |
| JJ-155 | MD | KM364004 | 128 | 19-Aug-2004 | m | a | Maryland | Washington | USA | Antietam National Battlefield | Josh Johnson |
| JJ-156 | MD |  | 126 | 19-Aug-2004 | f | a | Maryland | Washington | USA | Antietam National Battlefield | Josh Johnson |
| JJ-159 | MD | KM364005 | 129 | 19-Aug-2004 | f | j | Maryland | Washington | USA | Antietam National Battlefield | Josh Johnson |
| NY4901 | NY |  | 36 | 11-May-2010 | f | a | New York | Jefferson | USA | Fort Drum Bat House | Chris Dobony |
| NY4912 | NY |  | 36 | 2-Jun-2010 | f | a | New York | Jefferson | USA | Fort Drum Bat House | Chris Dobony |
| NY4913 | NY |  | 34 | 2-Jun-2010 | f | a | New York | Jefferson | USA | Fort Drum Bat House | Chris Dobony |
| NY4914 | NY |  | 36 | 2-Jun-2010 | f | a | New York | Jefferson | USA | Fort Drum Bat House | Chris Dobony |
| NY4915 | NY |  | 110 | 2-Jun-2010 | f | a | New York | Jefferson | USA | Fort Drum Bat House | Chris Dobony |
| NY4916 | NY | KM364006 | 130 | 2-Jun-2010 | f | a | New York | Jefferson | USA | Fort Drum Bat House | Chris Dobony |
| NY4917 | NY |  | 93 | 2-Jun-2010 | f | a | New York | Jefferson | USA | Fort Drum Bat House | Chris Dobony |
| NY4918 | NY |  | 36 | 2-Jun-2010 | f | a | New York | Jefferson | USA | Fort Drum Bat House | Chris Dobony |
| NY4919 | NY | KM364007 | 131 | 2-Jun-2010 | f | a | New York | Jefferson | USA | Fort Drum Bat House | Chris Dobony |
| NY4970 | NY | KM364008 | 132 | 11-May-2010 | f | a | New York | Jefferson | USA | Fort Drum Bat House | Chris Dobony |
| NY4973 | NY |  | 110 | 11-May-2010 | f | a | New York | Jefferson | USA | Fort Drum Bat House | Chris Dobony |
| NY4975 | NY |  | 117 | 11-May-2010 | f | a | New York | Jefferson | USA | Fort Drum Bat House | Chris Dobony |
| NY4976 | NY | KM364009 | 133 | 11-May-2010 | f | a | New York | Jefferson | USA | Fort Drum Bat House | Chris Dobony |
| NY4977 | NY | KM364010 | 134 | 11-May-2010 | f | a | New York | Jefferson | USA | Fort Drum Bat House | Chris Dobony |
| NY4978 | NY | KM364011 | 135 | 11-May-2010 | f | a | New York | Jefferson | USA | Fort Drum Bat House | Chris Dobony |
| NY4980 | NY | KM364012 | 136 | 11-May-2010 | f | a | New York | Jefferson | USA | Fort Drum Bat House | Chris Dobony |
| NY4982 | NY |  | 110 | 11-May-2010 | f | a | New York | Jefferson | USA | Fort Drum Bat House | Chris Dobony |
| NY4984 | NY | KM364013 | 137 | 11-May-2010 | f | a | New York | Jefferson | USA | Fort Drum Bat House | Chris Dobony |
| NY4991 | NY |  | 34 | 2-Jun-2010 | f | a | New York | Jefferson | USA | Fort Drum Bat House | Chris Dobony |
| NY4994 | NY |  | 36 | 11-May-2010 | f | a | New York | Jefferson | USA | Fort Drum Bat House | Chris Dobony |
| PT-01 | NJ-Mo | KM364014 | 138 | 4-May-2006 | f | a | New Jersey | Morris | USA | Picatinny Arsenal | John Van de Venter |
| PT-02 | NJ-Mo |  | 138 | 4-May-2006 | f | a | New Jersey | Morris | USA | Picatinny Arsenal | John Van de Venter |
| PT-03 | NJ-Mo |  | 120 | 4-May-2006 | m | a | New Jersey | Morris | USA | Picatinny Arsenal | John Van de Venter |
| PT-04 | NJ-Mo |  | 120 | 4-May-2006 | f | a | New Jersey | Morris | USA | Picatinny Arsenal | John Van de Venter |
| PT-05 | NJ-Mo |  | 120 | 4-May-2006 | f | a | New Jersey | Morris | USA | Picatinny Arsenal | John Van de Venter |
| PT-06 | NJ-Mo | KM364015 | 139 | 4-May-2006 | f | a | New Jersey | Morris | USA | Picatinny Arsenal | John Van de Venter |
| PT-07 | NJ-Mo |  | 110 | 4-May-2006 | f | a | New Jersey | Morris | USA | Picatinny Arsenal | John Van de Venter |
| PT-09 | NJ-Mo |  | 41 | 4-May-2006 | f | a | New Jersey | Morris | USA | Picatinny Arsenal | John Van de Venter |
| PT-10 | NJ-Mo |  | 110 | 4-May-2006 | f | a | New Jersey | Morris | USA | Picatinny Arsenal | John Van de Venter |
| PT-11 | NJ-Mo |  | 110 | 4-May-2006 | f | a | New Jersey | Morris | USA | Picatinny Arsenal | John Van de Venter |
| PT-12 | NJ-Mo |  | 110 | 4-May-2006 | f | a | New Jersey | Morris | USA | Picatinny Arsenal | John Van de Venter |
| PT-13 | NJ-Mo |  | 79 | 4-May-2006 | f | a | New Jersey | Morris | USA | Picatinny Arsenal | John Van de Venter |
| PT-14 | NJ-Mo |  | 36 | 4-May-2006 | f | a | New Jersey | Morris | USA | Picatinny Arsenal | John Van de Venter |
| PT-15 | NJ-Mo |  | 110 | 4-May-2006 | m | a | New Jersey | Morris | USA | Picatinny Arsenal | John Van de Venter |
| PT-16 | NJ-Mo | KM364016 | 140 | 4-May-2006 | f | a | New Jersey | Morris | USA | Picatinny Arsenal | John Van de Venter |
| PT-17 | NJ-Mo |  | 41 | 4-May-2006 | f | a | New Jersey | Morris | USA | Picatinny Arsenal | John Van de Venter |
| PT-18 | NJ-Mo |  | 36 | 4-May-2006 | f | a | New Jersey | Morris | USA | Picatinny Arsenal | John Van de Venter |
| PT-19 | NJ-Mo |  | 79 | 2-Jun-2006 | f | a | New Jersey | Morris | USA | Picatinny Arsenal | John Van de Venter |
| PT-21 | NJ-Mo |  | 111 | 2-Jun-2006 | f | a | New Jersey | Morris | USA | Picatinny Arsenal | John Van de Venter |
| PT-22 | NJ-Mo |  | 110 | 2-Jun-2006 | f | a | New Jersey | Morris | USA | Picatinny Arsenal | John Van de Venter |
| PT-23 | NJ-Mo |  | 36 | 2-Jun-2006 | f | a | New Jersey | Morris | USA | Picatinny Arsenal | John Van de Venter |
| PT-25 | NJ-Mo | KM364017 | 141 | 2-Jun-2006 | f | a | New Jersey | Morris | USA | Picatinny Arsenal | John Van de Venter |
| PT-26 | NJ-Mo |  | 41 | 2-Jun-2006 | f | a | New Jersey | Morris | USA | Picatinny Arsenal | John Van de Venter |
| PT-29 | NJ-Mo |  | 41 | 2-Jun-2006 | f | a | New Jersey | Morris | USA | Picatinny Arsenal | John Van de Venter |
| PT-30 | NJ-Mo | KM364018 | 142 | 2-Jun-2006 | f | a | New Jersey | Morris | USA | Picatinny Arsenal | John Van de Venter |
| PT-31 | NJ-Mo |  | 79 | 2-Jun-2006 | f | a | New Jersey | Morris | USA | Picatinny Arsenal | John Van de Venter |
| PT-38 | NJ-Mo |  | 116 | 2-Jun-2006 | f | a | New Jersey | Morris | USA | Picatinny Arsenal | John Van de Venter |
| PT-45 | NJ-Mo |  | 79 | 2-Jun-2006 | f | a | New Jersey | Morris | USA | Picatinny Arsenal | John Van de Venter |
| PT-46 | NJ-Mo |  | 110 | 2-Jun-2006 | f | a | New Jersey | Morris | USA | Picatinny Arsenal | John Van de Venter |
| PT-49 | NJ-Mo |  | 41 | 2-Jun-2006 | f | a | New Jersey | Morris | USA | Picatinny Arsenal | John Van de Venter |
| NJSAL01 | NJ-Sa |  | 110 | 3-Aug-2010 | f | j | New Jersey | Salem | USA | Supawna Meadows National Wildlife Refuge | Mick Valent |
| NJSAL02 | NJ-Sa |  | 116 | 3-Aug-2010 | f | a | New Jersey | Salem | USA | Supawna Meadows National Wildlife Refuge | Mick Valent |
| NJSAL03 | NJ-Sa |  | 36 | 3-Aug-2010 | f | a | New Jersey | Salem | USA | Supawna Meadows National Wildlife Refuge | Mick Valent |
| NJSAL04 | NJ-Sa | KM364019 | 143 | 3-Aug-2010 | f | a | New Jersey | Salem | USA | Supawna Meadows National Wildlife Refuge | Mick Valent |
| NJSAL05 | NJ-Sa |  | 110 | 3-Aug-2010 | f | a | New Jersey | Salem | USA | Supawna Meadows National Wildlife Refuge | Mick Valent |
| NJSAL06 | NJ-Sa |  | 36 | 3-Aug-2010 | f | a | New Jersey | Salem | USA | Supawna Meadows National Wildlife Refuge | Mick Valent |
| NJSAL07 | NJ-Sa |  | 36 | 3-Aug-2010 | m | a | New Jersey | Salem | USA | Supawna Meadows National Wildlife Refuge | Mick Valent |
| NJSAL08 | NJ-Sa |  | 116 | 3-Aug-2010 | f | j | New Jersey | Salem | USA | Supawna Meadows National Wildlife Refuge | Mick Valent |
| NJSAL09 | NJ-Sa |  | 110 | 3-Aug-2010 | f | j | New Jersey | Salem | USA | Supawna Meadows National Wildlife Refuge | Mick Valent |
| NJSAL10 | NJ-Sa |  | 36 | 3-Aug-2010 | f | j | New Jersey | Salem | USA | Supawna Meadows National Wildlife Refuge | Mick Valent |
| NJSAL11 | NJ-Sa |  | 102 | 3-Aug-2010 | f | j | New Jersey | Salem | USA | Supawna Meadows National Wildlife Refuge | Mick Valent |
| NJSAL12 | NJ-Sa |  | 36 | 3-Aug-2010 | f | a | New Jersey | Salem | USA | Supawna Meadows National Wildlife Refuge | Mick Valent |
| NJSAL13 | NJ-Sa | KM364020 | 144 | 3-Aug-2010 | f | a | New Jersey | Salem | USA | Supawna Meadows National Wildlife Refuge | Mick Valent |
| NJSAL14 | NJ-Sa |  | 113 | 3-Aug-2010 | f | a | New Jersey | Salem | USA | Supawna Meadows National Wildlife Refuge | Mick Valent |
| NJSAL15 | NJ-Sa |  | 110 | 3-Aug-2010 | f | a | New Jersey | Salem | USA | Supawna Meadows National Wildlife Refuge | Mick Valent |
| NJSAL16 | NJ-Sa |  | 102 | 3-Aug-2010 | f | j | New Jersey | Salem | USA | Supawna Meadows National Wildlife Refuge | Mick Valent |
| NJSAL17 | NJ-Sa |  | 36 | 3-Aug-2010 | f | j | New Jersey | Salem | USA | Supawna Meadows National Wildlife Refuge | Mick Valent |
| NJSAL18 | NJ-Sa |  | 36 | 3-Aug-2010 | f | j | New Jersey | Salem | USA | Supawna Meadows National Wildlife Refuge | Mick Valent |
| NJSAL19 | NJ-Sa |  | 102 | 3-Aug-2010 | f | j | New Jersey | Salem | USA | Supawna Meadows National Wildlife Refuge | Mick Valent |
| NJSAL20 | NJ-Sa |  | 110 | 3-Aug-2010 | f | j | New Jersey | Salem | USA | Supawna Meadows National Wildlife Refuge | Mick Valent |
| MD-02 | QB |  | 36 | 12-Jul-2003 | f | a | Quebec |  | Canada | de Lotbinière | Michel Delorme |
| MD-09 | QB |  | 110 | 12-Jul-2003 | f | a | Quebec |  | Canada | de Lotbinière | Michel Delorme |
| MD-19 | QB |  | 36 | 12-Jul-2003 | f | a | Quebec |  | Canada | de Lotbinière | Michel Delorme |
| MD-21 | QB |  | 141 | 12-Jul-2003 | f | a | Quebec |  | Canada | de Lotbinière | Michel Delorme |
| MD-22 | QB |  | 110 | 24-Jul-2003 | f | j | Quebec |  | Canada | St-Vallier | Michel Delorme |
| MD-23 | QB |  | 141 | 24-Jul-2003 | m | j | Quebec |  | Canada | St-Vallier | Michel Delorme |
| MD-24 | QB |  | 36 | 24-Jul-2003 | f | j | Quebec |  | Canada | St-Vallier | Michel Delorme |
| MD-26 | QB |  | 141 | 24-Jul-2003 | m | j | Quebec |  | Canada | St-Vallier | Michel Delorme |
| MD-27 | QB |  | 141 | 24-Jul-2003 | f | j | Quebec |  | Canada | St-Vallier | Michel Delorme |
| MD-29 | QB |  | 110 | 24-Jul-2003 | f | j | Quebec |  | Canada | St-Vallier | Michel Delorme |
| MD-32 | QB |  | 141 | 24-Jul-2003 | f | j | Quebec |  | Canada | St-Vallier | Michel Delorme |
| MD-54 | QB | KM364021 | 145 | 24-Jul-2003 | f | j | Quebec |  | Canada | St-Vallier | Michel Delorme |
| MD-57 | QB | KM364022 | 146 | 24-Jul-2003 | m | j | Quebec |  | Canada | St-Vallier | Michel Delorme |
| MD-62 | QB |  | 145 | 24-Jul-2003 | f | a | Quebec |  | Canada | St-Vallier | Michel Delorme |
| MD-64 | QB | KM364023 | 147 | 24-Jul-2003 | f | j | Quebec |  | Canada | St-Vallier | Michel Delorme |
| MD-68 | QB | KM364024 | 148 | 24-Jul-2003 | f | a | Quebec |  | Canada | St-Vallier | Michel Delorme |
