# Supplementary material for: Range-Wide Genetic Analysis of Little Brown Bat (Myotis lucifugus) Populations: Estimating the Risk of Spread of White-Nose Syndrome
Source: PLoS One. 2015 Jul 8;10(7):e0128713. doi: 10.1371/journal.pone.0128713 (PMC4495924; doi:10.1371/journal.pone.0128713)
Supplement: S2 Table — (DOCX) [file pone.0128713.s004.docx]

Table S2. Locus information for 11 microsatellites used to amplify *M. lucifugus*. Listed are load (combined PCR amplifications loaded on an ABI 3130 sequencer), PCR (multiplex panel and single-locus amplifications), annealing temperature (T_a_), whether or not an extension is required (‘yes’ requires a final 30 min extension at 72 °C, ‘no’ skips this step), the number of cycles (we used a two-step PCR: the basic conditions started with 3 cycles, followed by 33, 36, or 39 cycles [see main text]), locus (including forward and reverse primer sequence and fluorophore), volume in PCR reaction (using GE Healthcare Illustra Pure-Taq Ready-to-Go Beads, we added 2 μl template, the listed volume of each primer, and water to a total volume of 25 μl), and the citation for the locus.

| Load | PCR | T_a_ (°C) | Extension | PCR Cycles | Locus | Sequence | Volume (μl) | Citation |
| --- | --- | --- | --- | --- | --- | --- | --- | --- |
| 1 | 1 | 60 | yes | 33 | MS3D02 | PET-CTA AGA CCC TTT CCA GCT CTC A | 2.5 | Trujillo & Amelon 2009 |
|  |  |  |  |  |  | GAT ACC ATC ACT CTT TCC CCT G |  |  |
|  |  |  |  |  | IBat CA5 | VIC-CTC TGC CTC TTC ATG CTG CT | 1 | Oyler-McCance et al. 2011 |
|  |  |  |  |  |  | GGA GGC AAC CAA TTG ATG TT |  |  |
| 1 | 2 | 60 | yes | 33 | IBat CA47 | VIC-ACA AGT GCC TGT GGC TTC TT | 1 | Oyler-McCance et al. 2011 |
|  |  |  |  |  |  | GTA GGC GGC TTC ATC CTA CA |  |  |
|  |  |  |  |  | MM-E24 | FAM-GCA GGT TCA ATC CCT GAC C | 1 | Castella & Ruedi 2000 |
|  |  |  |  |  |  | AAA GCC AGA CTC CAA ATT CTG |  |  |
| 2 | 3 | 54 | no | 33 | Cora_F11_C04 | VIC-AAG CTC AGA GAC TGC TCC TTC | 1 | Piaggio et al. 2009 |
|  |  |  |  |  |  | ATC CAT TAT GTT TGC TGA TGT TC |  |  |
| 2 | 4 | 60 | no | 36 | IBat M23 | NED-ATC CTG GGT TTT GGG TTC AT | 1.8 | Oyler-McCance et al. 2011 |
|  |  |  |  |  |  | TCA TGT AAA TTT CAA AAA CAG CAA A |  |  |
|  |  |  |  |  | IBat CA43 | PET-TGC AGT CAT CTC AGC CTG TC | 1.5 | Oyler-McCance et al. 2011 |
|  |  |  |  |  |  | TTG GTG AGA GGC TCT GCT TT |  |  |
|  |  |  |  |  | MS3F05 | VIC-GTT TTG GTC TTT TGC TTT GTG G | 0.5 | Trujillo & Amelon 2009 |
|  |  |  |  |  |  | ACG ATT AGG CAG AGG TTG TGA T |  |  |
| 3 | 5 | 60 | no | 33 | Coto_G02F_H10R | VIC-AGA GTG CTT TTA TGG GCA AAT | 2.5 | Piaggio et al. 2009 |
|  |  |  |  |  |  | TGC TTG TAG TTC CCT TTC CTT |  |  |
|  |  |  |  |  | MM-G9 | NED-AGG GGA CAT ACA AGA ATC AAC C | 0.6 | Castella & Ruedi 2000 |
|  |  |  |  |  |  | TAA TTT CTC CAC TGA ACT CCC C |  |  |
| 3 | 6 | 60 | yes | 39 | IBat CA11 | PET-AGA ACC CAG TGC CCT TCT TT | 1 | Oyler-McCance et al. 2011 |
|  |  |  |  |  |  | ATG AGA GGG GGA TGT GAC AG |  |  |

Castella V, Ruedi M (2000) Characterization of highly variable microsatellite loci in the bat *Myotis myotis* (Chiroptera: Vespertilionidae). Molecular Ecology 9: 1000-1002.

Oyler-McCance SJ, Fike JA (2011) Characterization of small microsatellite loci isolated in endangered Indiana bat (*Myotis sodalis*) for use in non-invasive sampling. Conservation Genetics Resources 3: 243-245.

Piaggio AJ, Figueroa JA, Perkins SL (2009) Development and characterization of 15 polymorphic microsatellite loci isolated from Rafinesque's big-eared bat, *Corynorhinus rafinesquii*. Molecular Ecology Resources 9: 1191-1193.

Piaggio AJ, Miller KEG, Matocq MD, Perkins SL (2009) Eight polymorphic microsatellite loci developed and characterized from Townsend's big-eared bat, *Corynorhinus townsendii*. Molecular Ecology Resources 9: 258-260.

Trujillo RG, Amelon SK (2009) Development of microsatellite markers in *Myotis sodalis* and cross-species amplification in *M. grisescens*, *M. leibii*, *M. lucifugus*, and *M. septentrionalis*. Conservation Genetics 10: 1965-1968.
