# Supplementary material for: Range-Wide Genetic Analysis of Little Brown Bat (Myotis lucifugus) Populations: Estimating the Risk of Spread of White-Nose Syndrome
Source: PLoS One. 2015 Jul 8;10(7):e0128713. doi: 10.1371/journal.pone.0128713 (PMC4495924; doi:10.1371/journal.pone.0128713)
Supplement: S3 Table — (DOCX) [file pone.0128713.s005.docx]

Table S3. List of specimens (all in the genus *Myotis*), year of collection, sampling localities, and Genbank accession numbers for COI sequences used in phylogenetic analysis. Voucher ID’s are provided for museum tissues.

| ID | Taxon | Year | Country | Province/State | Voucher^1^ | Genbank | Reference | Source |
| --- | --- | --- | --- | --- | --- | --- | --- | --- |
| NMNH-568964 | Myotis austroriparius | 2003 | USA | Georgia | NMNH-568964 | GU723203 | Streicker et al. 2010 | National Museum of Natural History |
| ASK5450 | Myotis californicus | 1999 | USA | Texas | ASNHC-11511 | GU723208 | Streicker et al. 2010 | Angelo State Natural History Collection |
| DJ10 | Myotis californicus | 2001 | USA | California |  | GU723207 | Streicker et al. 2010 | Dave Johnston |
| DR03 | Myotis californicus | 2002 | Canada | British Columbia |  | KM382071 | This study | Daniella Rambaldini |
| NE25 | Myotis californicus | 2001 | USA | Nevada |  | GU723209 | Streicker et al. 2010 | Mike O'Farrell |
| TA7884 | Myotis californicus | 2002 | Mexico | Baja California Sur | CIBN-7884 | KM382072 | This study | Centro de Investigaciones Biológicas del Noroeste S.C. |
| TA8072 | Myotis californicus | 2003 | Mexico | Sonora | CIBN-8072 | KM382073 | This study | Centro de Investigaciones Biológicas del Noroeste S.C. |
| TA8080 | Myotis californicus | 2002 | Mexico | Baja California Sur | CIBN-8077 | KM382074 | This study | Centro de Investigaciones Biológicas del Noroeste S.C. |
| TA8612 | Myotis californicus | 2003 | Mexico | Baja California Sur | CIBN-8080 | KM382075 | This study | Centro de Investigaciones Biológicas del Noroeste S.C. |
| TA8614 | Myotis californicus | 2003 | Mexico | Baja California Sur | CIBN-8612 | KM382076 | This study | Centro de Investigaciones Biológicas del Noroeste S.C. |
| ASK5170 | Myotis ciliolabrum | 2001 | USA | Arizona | CIBN-8614 | GU723210 | Streicker et al. 2010 | Angelo State Natural History Collection |
| ASK6044 | Myotis ciliolabrum | 2002 | USA | Texas | ASNHC12236 | KM382077 | This study | Angelo State Natural History Collection |
| BI15 | Myotis ciliolabrum | 2000 | Canada | Alberta |  | KM382078 | This study | Cori Lausen |
| FG4888 | Myotis ciliolabrum | 2001 | USA | Idaho |  | KM382079 | This study | Kari Getz |
| KT28 | Myotis ciliolabrum | 2002 | USA | New Mexico |  | GU723212 | Streicker et al. 2010 | Kate Thibault |
| NE14 | Myotis ciliolabrum | 2001 | USA | Nevada |  | KM382080 | This study | Mike O'Farrell |
| RB5863 | Myotis ciliolabrum | 2004 | USA | Nebraska |  | KM382081 | This study | Russ Benedict |
| RB5880 | Myotis ciliolabrum | 2004 | USA | Nebraska |  | KM382082 | This study | Russ Benedict |
| CO03 | Myotis evotis | 2001 | USA | Colorado |  | KM382083 | This study | Kirk Navo |
| DJ03 | Myotis evotis | 2001 | USA | California |  | KM382084 | This study | Dave Johnston |
| FG4578 | Myotis evotis | 2001 | USA | Idaho |  | KM382085 | This study | Kari Getz |
| MK01 | Myotis evotis | 2002 | Canada | British Columbia |  | KM382086 | This study | Mandy Kellner |
| MVZ201321 | Myotis evotis | 2003 | USA | California | MVZ-201321 | GU723213 | Streicker et al. 2010 | Museum of Vertebrate Zoology |
| MVZ206955 | Myotis evotis | 2002 | USA | California | MVZ-206955 | KM382087 | This study | Museum of Vertebrate Zoology |
| NE01 | Myotis evotis | 2001 | USA | Nevada |  | KM382088 | This study | Mike O'Farrell |
| NE51 | Myotis evotis | 2001 | USA | Nevada |  | GU723214 | Streicker et al. 2010 | Mike O'Farrell |
| POV9783 | Myotis evotis | 1997 | Canada | British Columbia |  | KM382089 | This study | Maarten Vonhof |
| TA4884 | Myotis evotis | 1999 | Mexico | Baja California Sur | CIBN-4884 | KM382090 | This study | Centro de Investigaciones Biológicas del Noroeste S.C. |
| TA8610 | Myotis evotis | 2003 | Mexico | Baja California Sur | CIBN-8610 | KM382091 | This study | Centro de Investigaciones Biológicas del Noroeste S.C. |
| UAM66651 | Myotis evotis | 1999 | USA | New Mexico | UAM-66651 | KM382092 | This study | University of Alaska Museum |
| UH39 | Myotis evotis | 2002 | USA | Utah |  | KM382093 | This study | Kate Grandison |
| UH43 | Myotis evotis | 2002 | USA | Utah |  | KM382094 | This study | Kate Grandison |
| UAM23338 | Myotis keenii | 1993 | USA | Alaska | UAM-23338 | GU723218 | Streicker et al. 2010 | University of Alaska Museum |
| AR39 | Myotis leibii | 2004 | USA | Arkansas |  | GU723220 | Streicker et al. 2010 | David Saugey |
| CS02 | Myotis leibii | 2002 | USA | West Virginia |  | GU723219 | Streicker et al. 2010 | Craig Stihler |
| CS181 | Myotis leibii | 2006 | USA | West Virginia |  | KM382095 | This study | Craig Stihler |
| EB308 | Myotis leibii | 2004 | USA | North Carolina |  | KM382096 | This study | Eric Britzke |
| EB311 | Myotis leibii | 2004 | USA | North Carolina |  | KM382097 | This study | Eric Britzke |
| MG71 | Myotis leibii | 2006 | USA | Kentucky |  | KM382098 | This study | Mark Gumbert |
| CM82047 | Myotis septentrionalis | 1986 | USA | West Virginia | CM-82047 | GU723226 | Streicker et al. 2010 | Carnegie Museum of Natural History |
| CS11 | Myotis septentrionalis | 2002 | USA | West Virginia |  | KM382099 | This study | Craig Stihler |
| EB95 | Myotis septentrionalis | 2002 | USA | Kentucky |  | KM382100 | This study | Eric Britzke |
| FN9745 | Myotis septentrionalis | 1997 | Canada | British Columbia |  | KM382101 | This study | Steve McNalley |
| KAL01 | Myotis septentrionalis | 2007 | USA | North Carolina |  | KM382102 | This study | Matina Kalcounis-Ruppell |
| KAL06 | Myotis septentrionalis | 2007 | USA | North Carolina |  | KM382103 | This study | Matina Kalcounis-Ruppell |
| RB5610 | Myotis septentrionalis | 2004 | USA | Nebraska |  | GU723227 | Streicker et al. 2010 | Russ Benedict |
| MYSO01 | Myotis sodalis | 1997 | USA | Kentucky |  | GU723229 | Streicker et al. 2010 | Bob Currie |
| MYSO191 | Myotis sodalis | 1998 | USA | Missouri |  | KM382104 | This study | Rick Clawson |
| MYSO192 | Myotis sodalis | 1998 | USA | Missouri |  | KM382105 | This study | Rick Clawson |
| MYSO193 | Myotis sodalis | 1998 | USA | Missouri |  | KM382106 | This study | Rick Clawson |
| MYSO270 | Myotis sodalis | 1999 | USA | Indiana |  | KM382107 | This study | Bob Currie |
| MYSO282 | Myotis sodalis | 1999 | USA | Indiana |  | KM382108 | This study | Bob Currie |
| MYSO313 | Myotis sodalis | 1999 | USA | Tennessee |  | KM382109 | This study | Bob Currie |
| MYSO315 | Myotis sodalis | 1999 | USA | Tennessee |  | KM382110 | This study | Bob Currie |
| MYSO329 | Myotis sodalis | 1999 | USA | Virginia |  | KM382111 | This study | Bob Currie |
| MYSO357 | Myotis sodalis | 1999 | USA | West Virginia |  | KM382112 | This study | Craig Stihler |
| MYSO378 | Myotis sodalis | 1999 | USA | West Virginia |  | KM382113 | This study | Craig Stihler |
| MYSO612 | Myotis sodalis | 2000 | USA | Tennessee |  | GU723230 | Streicker et al. 2010 | Mick Harvey |
| GP1 | Myotis sodalis | 2003 | USA | New York |  | KM382114 | This study | Al Hicks |
| H5 | Myotis sodalis | 2003 | USA | New York |  | KM382115 | This study | Al Hicks |
| JO02 | Myotis sodalis | 2007 | USA | North Carolina |  | KM382116 | This study | Joy O'Keefe |
| ASK6889 | Myotis thysanodes | 2004 | USA | Texas | ASNHC-12975 | GU723232 | Streicker et al. 2010 | Angelo State Natural History Collection |
| CO08 | Myotis thysanodes | 2001 | USA | Colorado |  | GU723234 | Streicker et al. 2010 | Kirk Navo |
| DR09 | Myotis thysanodes | 2002 | Canada | British Columbia |  | GU723233 | Streicker et al. 2010 | Daniella Rambaldini |
| KT12 | Myotis thysanodes | 2002 | USA | New Mexico |  | KM382117 | This study | Kate Thibault |
| UH02 | Myotis thysanodes | 2002 | USA | Utah |  | KM382118 | This study | Kate Grandison |
| ASK6043 | Myotis volans | 2002 | USA | Texas | ASNHC-12237 | KM382119 | This study | Angelo State Natural History Collection |
| CL15 | Myotis volans | 2003 | USA | Montana |  | KM382120 | This study | Cori Lausen |
| CL17 | Myotis volans | 2003 | Canada | Alberta |  | KM382121 | This study | Cori Lausen |
| CO02 | Myotis volans | 2001 | USA | Colorado |  | KM382122 | This study | Kirk Navo |
| DJ02 | Myotis volans | 2001 | USA | California |  | KM382123 | This study | Dave Johnston |
| DP100 | Myotis volans | 2002 | USA | California |  | KM382124 | This study | Dixie Pierson |
| DP101 | Myotis volans | 2002 | USA | California |  | KM382125 | This study | Dixie Pierson |
| FN9730 | Myotis volans | 1997 | Canada | British Columbia |  | KM382126 | This study | Steve McNalley |
| FN9836 | Myotis volans | 1998 | Canada | British Columbia |  | KM382127 | This study | Maarten Vonhof |
| JW02 | Myotis volans | 2001 | USA | Idaho |  | GU723241 | Streicker et al. 2010 | Jo Wenger |
| JZ146 | Myotis volans | 2001 | USA | Oregon |  | KM382128 | This study | Jan Zinck |
| JZ152 | Myotis volans | 2001 | USA | Oregon |  | KM382129 | This study | Jan Zinck |
| JZ153 | Myotis volans | 2001 | USA | Oregon |  | KM382130 | This study | Jan Zinck |
| JZ159 | Myotis volans | 2001 | USA | Oregon |  | KM382131 | This study | Jan Zinck |
| MVZ198299 | Myotis volans | 2000 | USA | California | MVZ-198299 | GU723239 | Streicker et al. 2010 | Museum of Vertebrate Zoology |
| NC27 | Myotis volans | 2002 | USA | Washington |  | KM382132 | This study | Roger Christophersen |
| NE05 | Myotis volans | 2001 | USA | Nevada |  | KM382133 | This study | Mike O'Farrell |
| NE13 | Myotis volans | 2001 | USA | Nevada |  | KM382134 | This study | Mike O'Farrell |
| NE18 | Myotis volans | 2001 | USA | Nevada |  | KM382135 | This study | Mike O'Farrell |
| NE41 | Myotis volans | 2001 | USA | Nevada |  | KM382136 | This study | Mike O'Farrell |
| PC19 | Myotis volans | 2002 | USA | New Mexico |  | KM382137 | This study | Paul Cryan |
| POV9708 | Myotis volans | 1997 | Canada | British Columbia |  | KM382138 | This study | Maarten Vonhof |
| POV9788 | Myotis volans | 1997 | Canada | British Columbia |  | KM382139 | This study | Maarten Vonhof |
| RAB5315 | Myotis volans | 2002 | USA | Idaho |  | KM382140 | This study | Kari Getz |
| RAF02 | Myotis volans | 2002 | Mexico | Distrito Federal |  | KM382141 | This study | Rafael Avila-Flores |
| TK78915 | Myotis volans | 1998 | USA | Texas | TK78915 | KM382142 | This study | Museum, Texas Tech University |
| TK78980 | Myotis volans | 1998 | USA | Texas | TK78980 | KM382143 | This study | Museum, Texas Tech University |
| UAM19757 | Myotis volans | 1991 | USA | Alaska | UAM-19757 | KM382144 | This study | University of Alaska Museum |
| WY127 | Myotis volans | 2001 | USA | Wyoming |  | KM382145 | This study | Jeff Gruver |
| WY145 | Myotis volans | 2001 | USA | Wyoming |  | KM382146 | This study | Jeff Gruver |
| WY176 | Myotis volans | 2001 | USA | Wyoming |  | GU723238 | Streicker et al. 2010 | Jeff Gruver |
| WY232 | Myotis volans | 2001 | USA | Wyoming |  | KM382147 | This study | Jeff Gruver |

^1^ ASNHC = Angelo State Natural History Collection, CIBN = Centro de Investigaciones Biológicas del Noroeste S.C., CM = Carnegie Museum of Natural History, MVZ = Museum of Vertebrate Zoology at Berkeley, NMNH = National Museum of Natural History, TK = Museum of Texas Tech University, UAM = University of Alaska Museum of the North
