# Supplementary material for: Range-Wide Genetic Analysis of Little Brown Bat (Myotis lucifugus) Populations: Estimating the Risk of Spread of White-Nose Syndrome
Source: PLoS One. 2015 Jul 8;10(7):e0128713. doi: 10.1371/journal.pone.0128713 (PMC4495924; doi:10.1371/journal.pone.0128713)
Supplement: S4 Table — (DOCX) [file pone.0128713.s006.docx]

Table S4. Diversity of microsatellite loci, including observed (*H*_O_) and expected heterozygosity (*H*_E_), number of alleles (*N*_A_), allelic richness (*AR*) and the inbreeding coefficient (*F*_IS_).

| Locus | *H*_O_ | *H*_E_ | *N*_A_ | *AR* | *F*_IS_ |
| --- | --- | --- | --- | --- | --- |
| Ca5 | 0.871 | 0.882 | 32 | 13.77 | 0.011 |
| Ca11 | 0.884 | 0.862 | 16 | 9.73 | -0.026 |
| Ca43 | 0.885 | 0.919 | 32 | 13.48 | 0.036 |
| Ca47 | 0.917 | 0.91 | 18 | 12.59 | -0.007 |
| M23 | 0.866 | 0.911 | 26 | 12.75 | 0.049 |
| MS3D02 | 0.874 | 0.915 | 26 | 13.43 | 0.045 |
| MS3F05 | 0.894 | 0.895 | 25 | 12.56 | 0.001 |
| F11_C04 | 0.803 | 0.858 | 27 | 13.08 | 0.064 |
| MM-G9 | 0.919 | 0.926 | 28 | 14.04 | 0.007 |
| Overall | 0.879 | 0.897 | 25.6 | 12.82 | 0.02 |
