# Supplementary material for: Range-Wide Genetic Analysis of Little Brown Bat (Myotis lucifugus) Populations: Estimating the Risk of Spread of White-Nose Syndrome
Source: PLoS One. 2015 Jul 8;10(7):e0128713. doi: 10.1371/journal.pone.0128713 (PMC4495924; doi:10.1371/journal.pone.0128713)
Supplement: S5 Table — (DOCX) [file pone.0128713.s007.docx]

**Table S5.** Pairwise Φ_ST_ values among populations based on mtDNA COI sequences. The majority of pairwise comparisons were significant (*P*< 0.05). Non-significant pairwise comparisons are denoted with brackets.

|  | AK | CA-Ma | CA-Mo | CA-Sh | CA-Si | WA | BC-S | ID | BC-N | AB-N | AB-S | WY | MB | ON-1 |
| --- | --- | --- | --- | --- | --- | --- | --- | --- | --- | --- | --- | --- | --- | --- |
| AK | − |  |  |  |  |  |  |  |  |  |  |  |  |  |
| CA-Ma | 0.937 | − |  |  |  |  |  |  |  |  |  |  |  |  |
| CA-Mo | 0.955 | 0.858 | − |  |  |  |  |  |  |  |  |  |  |  |
| CA-Sh | 0.517 | 0.939 | 0.961 | − |  |  |  |  |  |  |  |  |  |  |
| CA-Si | 0.748 | 0.925 | 0.944 | 0.654 | − |  |  |  |  |  |  |  |  |  |
| WA | 0.696 | 0.493 | 0.504 | 0.632 | 0.722 | − |  |  |  |  |  |  |  |  |
| BC-S | 0.778 | 0.632 | 0.638 | 0.743 | 0.792 | 0.157 | − |  |  |  |  |  |  |  |
| ID | 0.903 | 0.626 | 0.771 | 0.892 | 0.904 | 0.619 | 0.706 | − |  |  |  |  |  |  |
| BC-N | 0.880 | 0.824 | 0.864 | 0.855 | 0.882 | 0.628 | 0.673 | 0.848 | − |  |  |  |  |  |
| AB-N | 0.915 | 0.884 | 0.906 | 0.905 | 0.915 | 0.710 | 0.730 | 0.884 | (0.022) | − |  |  |  |  |
| AB-S | 0.731 | 0.613 | 0.669 | 0.688 | 0.750 | 0.488 | 0.537 | 0.690 | (0.058) | 0.069 | − |  |  |  |
| WY | 0.764 | 0.565 | 0.639 | 0.727 | 0.780 | 0.268 | 0.232 | 0.604 | 0.685 | 0.739 | 0.549 | − |  |  |
| MB | 0.936 | 0.908 | 0.935 | 0.928 | 0.930 | 0.645 | 0.686 | 0.892 | 0.119 | 0.115 | 0.065 | 0.693 | − |  |
| ON-1 | 0.925 | 0.895 | 0.919 | 0.913 | 0.923 | 0.716 | 0.745 | 0.897 | 0.492 | 0.656 | 0.419 | 0.747 | 0.633 | − |
| MN | 0.921 | 0.889 | 0.914 | 0.911 | 0.919 | 0.684 | 0.711 | 0.885 | (0.023) | (-0.014) | (0.061) | 0.720 | (0.067) | 0.636 |
| WI-Ma | 0.909 | 0.871 | 0.899 | 0.895 | 0.908 | 0.676 | 0.705 | 0.874 | 0.067 | 0.049 | 0.074 | 0.713 | (0.066) | 0.622 |
| WI-Sa | 0.925 | 0.895 | 0.918 | 0.915 | 0.922 | 0.692 | 0.716 | 0.890 | 0.125 | 0.122 | 0.103 | 0.725 | 0.104 | 0.653 |
| KY | 0.917 | 0.894 | 0.908 | 0.906 | 0.918 | 0.712 | 0.668 | 0.906 | 0.763 | 0.810 | 0.585 | 0.703 | 0.791 | 0.828 |
| OH | 0.896 | 0.857 | 0.882 | 0.883 | 0.899 | 0.705 | 0.725 | 0.868 | 0.082 | 0.069 | 0.105 | 0.735 | (0.046) | 0.613 |
| TN | 0.971 | 0.969 | 0.977 | 0.974 | 0.965 | 0.780 | 0.781 | 0.940 | 0.266 | 0.324 | 0.203 | 0.792 | 0.591 | 0.821 |
| WV | 0.898 | 0.858 | 0.887 | 0.882 | 0.899 | 0.659 | 0.689 | 0.868 | 0.154 | 0.210 | 0.115 | 0.704 | 0.294 | 0.646 |
| ON-2 | 0.935 | 0.916 | 0.932 | 0.930 | 0.933 | 0.736 | 0.749 | 0.905 | 0.095 | 0.108 | 0.125 | 0.760 | 0.296 | 0.721 |
| PA | 0.896 | 0.856 | 0.886 | 0.880 | 0.897 | 0.670 | 0.702 | 0.865 | 0.097 | 0.175 | 0.127 | 0.713 | 0.273 | 0.623 |
| MD | 0.919 | 0.894 | 0.908 | 0.911 | 0.920 | 0.733 | 0.747 | 0.892 | 0.228 | 0.273 | 0.189 | 0.759 | 0.386 | 0.714 |
| NY | 0.931 | 0.908 | 0.929 | 0.923 | 0.927 | 0.692 | 0.716 | 0.896 | 0.120 | 0.157 | 0.114 | 0.728 | 0.314 | 0.699 |
| NJ-Mo | 0.920 | 0.891 | 0.910 | 0.911 | 0.920 | 0.718 | 0.734 | 0.888 | 0.155 | 0.166 | 0.132 | 0.746 | 0.261 | 0.693 |
| NJ-Sa | 0.909 | 0.876 | 0.903 | 0.896 | 0.909 | 0.679 | 0.709 | 0.878 | 0.141 | 0.229 | 0.150 | 0.721 | 0.337 | 0.653 |
| QB | 0.864 | 0.807 | 0.845 | 0.836 | 0.868 | 0.567 | 0.590 | 0.841 | 0.229 | 0.314 | 0.099 | 0.623 | 0.329 | 0.605 |

|  | MN | WI-Ma | WI-Sa | KY | OH | TN | WV | ON-2 | PA | MD | NY | NJ-Mo | NJ-Sa |
| --- | --- | --- | --- | --- | --- | --- | --- | --- | --- | --- | --- | --- | --- |
| AK |  |  |  |  |  |  |  |  |  |  |  |  |  |
| CA-Ma |  |  |  |  |  |  |  |  |  |  |  |  |  |
| CA-Mo |  |  |  |  |  |  |  |  |  |  |  |  |  |
| CA-Sh |  |  |  |  |  |  |  |  |  |  |  |  |  |
| CA-Si |  |  |  |  |  |  |  |  |  |  |  |  |  |
| WA |  |  |  |  |  |  |  |  |  |  |  |  |  |
| BC-S |  |  |  |  |  |  |  |  |  |  |  |  |  |
| ID |  |  |  |  |  |  |  |  |  |  |  |  |  |
| BC-N |  |  |  |  |  |  |  |  |  |  |  |  |  |
| AB-N |  |  |  |  |  |  |  |  |  |  |  |  |  |
| AB-S |  |  |  |  |  |  |  |  |  |  |  |  |  |
| WY |  |  |  |  |  |  |  |  |  |  |  |  |  |
| MB |  |  |  |  |  |  |  |  |  |  |  |  |  |
| ON-1 |  |  |  |  |  |  |  |  |  |  |  |  |  |
| MN | − |  |  |  |  |  |  |  |  |  |  |  |  |
| WI-Ma | 0.041 | − |  |  |  |  |  |  |  |  |  |  |  |
| WI-Sa | 0.100 | (0.049) | − |  |  |  |  |  |  |  |  |  |  |
| KY | 0.801 | 0.789 | 0.802 | − |  |  |  |  |  |  |  |  |  |
| OH | (0.025) | (0.034) | 0.060 | 0.795 | − |  |  |  |  |  |  |  |  |
| TN | 0.408 | 0.258 | 0.363 | 0.864 | 0.294 | − |  |  |  |  |  |  |  |
| WV | 0.234 | 0.151 | 0.220 | 0.768 | 0.203 | 0.153 | − |  |  |  |  |  |  |
| ON-2 | 0.153 | 0.086 | 0.170 | 0.829 | 0.123 | 0.065 | 0.094 | − |  |  |  |  |  |
| PA | 0.205 | 0.125 | 0.204 | 0.786 | 0.177 | 0.068 | (0.030) | (0.037) | − |  |  |  |  |
| MD | 0.306 | 0.220 | 0.288 | 0.818 | 0.255 | 0.207 | 0.112 | 0.153 | 0.097 | − |  |  |  |
| NY | 0.203 | 0.123 | 0.224 | 0.809 | 0.165 | 0.142 | (0.041) | (0.027) | (0.002) | 0.130 | − |  |  |
| NJ-Mo | 0.194 | 0.092 | 0.165 | 0.815 | 0.151 | 0.153 | 0.065 | 0.061 | (0.032) | 0.082 | (0.039) | − |  |
| NJ-Sa | 0.265 | 0.175 | 0.253 | 0.796 | 0.226 | 0.138 | (0.009) | 0.090 | (-0.019) | 0.098 | (0.018) | 0.063 | − |
| QB | 0.318 | 0.260 | 0.313 | 0.656 | 0.309 | 0.343 | 0.145 | 0.272 | 0.177 | 0.262 | 0.210 | 0.238 | 0.193 |
