# Supplementary material for: Range-Wide Genetic Analysis of Little Brown Bat (Myotis lucifugus) Populations: Estimating the Risk of Spread of White-Nose Syndrome
Source: PLoS One. 2015 Jul 8;10(7):e0128713. doi: 10.1371/journal.pone.0128713 (PMC4495924; doi:10.1371/journal.pone.0128713)
Supplement: S6 Table — (DOCX) [file pone.0128713.s008.docx]

**Table S6.** Pairwise *F*_ST_ (lower diagonal) and Jost’s *D* (upper diagonal) based on nucDNA microsatellites. Significant *F*_ST_ values based on 10,000 permutations (*P* < 0.0002 following Bonferroni correction) are denoted with an *.

|  | AK | CA-Si | WA | BC-S | ID | BC-N | AB-N | AB-S | WY | MN | WI-Ma | WI-Sa | MI | KY |
| --- | --- | --- | --- | --- | --- | --- | --- | --- | --- | --- | --- | --- | --- | --- |
| AK | − | 0.419 | 0.326 | 0.383 | 0.429 | 0.432 | 0.453 | 0.376 | 0.462 | 0.567 | 0.507 | 0.454 | 0.545 | 0.509 |
| CA-Si | 0.06* | − | 0.023 | 0.037 | 0.046 | 0.151 | 0.095 | 0.073 | 0.112 | 0.279 | 0.199 | 0.174 | 0.284 | 0.216 |
| WA | 0.049* | 0.002 | − | 0.019 | 0.070 | 0.136 | 0.081 | 0.038 | 0.182 | 0.334 | 0.178 | 0.189 | 0.304 | 0.217 |
| BC-S | 0.058* | 0.004 | 0.002 | − | 0.100 | 0.152 | 0.123 | 0.065 | 0.105 | 0.310 | 0.215 | 0.254 | 0.281 | 0.252 |
| ID | 0.065* | 0.004 | 0.007 | 0.011* | − | 0.224 | 0.156 | 0.122 | 0.079 | 0.348 | 0.260 | 0.278 | 0.391 | 0.288 |
| BC-N | 0.064* | 0.013* | 0.012* | 0.015* | 0.022* | − | -0.033 | 0.042 | 0.125 | 0.044 | 0.03 | -0.025 | 0.088 | 0.011 |
| AB-N | 0.064* | 0.008* | 0.008* | 0.012* | 0.015* | -0.003 | − | 0.016 | 0.110 | 0.091 | 0.024 | 0.051 | 0.094 | 0.021 |
| AB-S | 0.054* | 0.006 | 0.004 | 0.006 | 0.012* | 0.004 | 0.001 | − | 0.127 | 0.099 | 0.029 | 0.068 | 0.092 | 0.078 |
| WY | 0.069* | 0.011* | 0.018* | 0.011* | 0.008* | 0.012* | 0.011* | 0.012* | − | 0.213 | 0.197 | 0.221 | 0.276 | 0.219 |
| MN | 0.088* | 0.028* | 0.034* | 0.034* | 0.037* | 0.005 | 0.009* | 0.010* | 0.023* | − | -0.009 | 0.109 | -0.004 | -0.013 |
| WI-Ma | 0.079* | 0.020* | 0.018* | 0.024* | 0.030* | 0.003 | 0.002 | 0.003 | 0.021* | -0.001 | − | 0.024 | 0.039 | -0.017 |
| WI-Sa | 0.069* | 0.016* | 0.018* | 0.026* | 0.027* | -0.002 | 0.005 | 0.007 | 0.023* | 0.012* | 0.003 | − | 0.113 | 0.045 |
| MI | 0.089* | 0.030* | 0.034* | 0.033* | 0.045* | 0.010 | 0.011* | 0.01* | 0.032* | -0.001 | 0.005 | 0.013 | − | 0.008 |
| KY | 0.076* | 0.021* | 0.022* | 0.027* | 0.030* | 0.001 | 0.002 | 0.008 | 0.023* | -0.002 | -0.002 | 0.005 | 0.001 | − |
| OH | 0.086* | 0.026* | 0.030* | 0.037* | 0.036* | 0.007 | 0.009* | 0.01* | 0.026* | -0.002 | -0.005 | 0.006 | 0.005 | -0.001 |
| TN | 0.076* | 0.020* | 0.022* | 0.027* | 0.030* | -0.002 | 0.003 | 0.009* | 0.019* | 0.000 | -0.001 | 0.003 | 0.004 | -0.001 |
| PA | 0.076* | 0.025* | 0.026* | 0.026* | 0.029* | 0.004 | 0.002 | 0.004 | 0.019* | -0.002 | -0.004 | 0.007 | 0.003 | 0.000 |
| MD | 0.079* | 0.025* | 0.026* | 0.028* | 0.036* | -0.003 | 0.007 | 0.007* | 0.024* | 0.001 | 0.000 | -0.001 | 0.008 | 0.001 |
| NY | 0.083* | 0.017* | 0.019* | 0.024* | 0.033* | -0.001 | -0.001 | 0.007 | 0.02* | -0.001 | -0.003 | -0.001 | 0.003 | -0.002 |
| NJ-Mo | 0.081* | 0.027* | 0.031* | 0.033* | 0.036* | 0.000 | 0.007 | 0.008* | 0.024* | -0.003 | -0.002 | 0.006 | 0.003 | 0.001 |
| QB | 0.078* | 0.025* | 0.023* | 0.031* | 0.032* | 0.000 | 0.004 | 0.007* | 0.023* | 0.000 | -0.004 | 0.000 | 0.006 | -0.002 |

|  | OH | TN | PA | MD | NY | NJ-Mo | QB |
| --- | --- | --- | --- | --- | --- | --- | --- |
| AK | 0.530 | 0.520 | 0.488 | 0.518 | 0.540 | 0.524 | 0.501 |
| CA-Si | 0.244 | 0.211 | 0.253 | 0.248 | 0.178 | 0.264 | 0.245 |
| WA | 0.272 | 0.222 | 0.251 | 0.249 | 0.191 | 0.299 | 0.214 |
| BC-S | 0.317 | 0.264 | 0.236 | 0.262 | 0.228 | 0.297 | 0.282 |
| ID | 0.306 | 0.297 | 0.266 | 0.337 | 0.315 | 0.335 | 0.286 |
| BC-N | 0.057 | -0.013 | 0.041 | -0.029 | -0.014 | 0.001 | 0.000 |
| AB-N | 0.080 | 0.026 | 0.016 | 0.063 | -0.005 | 0.063 | 0.036 |
| AB-S | 0.088 | 0.092 | 0.036 | 0.073 | 0.074 | 0.080 | 0.063 |
| WY | 0.219 | 0.183 | 0.173 | 0.218 | 0.188 | 0.215 | 0.201 |
| MN | -0.018 | 0.006 | -0.021 | 0.011 | -0.007 | -0.024 | 0.005 |
| WI-Ma | -0.037 | -0.007 | -0.031 | -0.001 | -0.027 | -0.017 | -0.031 |
| WI-Sa | 0.049 | 0.031 | 0.059 | -0.011 | -0.005 | 0.057 | -0.001 |
| MI | 0.039 | 0.032 | 0.023 | 0.061 | 0.024 | 0.02 | 0.05 |
| KY | -0.012 | -0.014 | 0.004 | 0.011 | -0.022 | 0.008 | -0.016 |
| OH | − | 0.033 | 0.019 | 0.039 | -0.004 | 0.024 | 0.007 |
| TN | 0.004 | − | -0.004 | -0.01 | -0.014 | 0.017 | -0.017 |
| PA | 0.002 | -0.001 | − | 0.019 | 0.035 | -0.021 | -0.016 |
| MD | 0.005 | -0.001 | 0.002 | − | -0.014 | 0.013 | -0.012 |
| NY | 0.000 | -0.002 | 0.004 | -0.002 | − | 0.024 | -0.028 |
| NJ-Mo | 0.003 | 0.002 | -0.003 | 0.002 | 0.003 | − | 0.010 |
| QB | 0.001 | -0.002 | -0.002 | -0.001 | -0.003 | 0.001 | − |
